# Aphid Transmission of *Potyvirus*: The Largest Plant-Infecting RNA Virus Genus

**DOI:** 10.3390/v12070773

**Published:** 2020-07-17

**Authors:** Kiran R. Gadhave, Saurabh Gautam, David A. Rasmussen, Rajagopalbabu Srinivasan

**Affiliations:** 1 Department of Plant Pathology and Microbiology, University of California, Riverside, CA 92521, USA; 2 Department of Entomology and Plant Pathology, North Carolina State University, Raleigh, NC 27606, USA; drasmus@ncsu.edu; 3 Department of Entomology, University of Georgia, 1109 Experiment Street, Griffin, GA 30223, USA; sg37721@uga.edu (S.G.); babusri@uga.edu (R.S.)

**Keywords:** potyviruses, vector–virus interactions, aphid transmission, plant viruses, insect vectors

## Abstract

Potyviruses are the largest group of plant infecting RNA viruses that cause significant losses in a wide range of crops across the globe. The majority of viruses in the genus *Potyvirus* are transmitted by aphids in a non-persistent, non-circulative manner and have been extensively studied vis-à-vis their structure, taxonomy, evolution, diagnosis, transmission, and molecular interactions with hosts. This comprehensive review exclusively discusses potyviruses and their transmission by aphid vectors, specifically in the light of several virus, aphid and plant factors, and how their interplay influences potyviral binding in aphids, aphid behavior and fitness, host plant biochemistry, virus epidemics, and transmission bottlenecks. We present the heatmap of the global distribution of potyvirus species, variation in the potyviral coat protein gene, and top aphid vectors of potyviruses. Lastly, we examine how the fundamental understanding of these multi-partite interactions through multi-omics approaches is already contributing to, and can have future implications for, devising effective and sustainable management strategies against aphid-transmitted potyviruses to global agriculture.

## 1. Introduction

A vast majority of plant viruses rely on insect vectors for their plant-to-plant spread. Aphids are arguably the most successful vectors of plants viruses, including potyviruses, due to an array of generic and specific features they possess [1]. The generic features include (i) the precise delivery of virus particles (virions) through a needle-like stylet into a host cell, (ii) parthenogenetic mode of reproduction efficiently producing abundant progeny within a short span of time, (iii) diverse modes of feeding allowing access to host plants across several families and (iv) unique adaptations such as overwintering egg stage facilitating survival in adverse conditions and wing formation allowing aphids and viruses to disseminate over long distances [2,3]. The specific features of aphids that enable the transmission of certain plant viruses over the others are a result of co-evolution of aphids and viruses over thousands of years [4,5]. For instance, aphid-transmitted non-persistent viruses have unique binding sites and strategies, transmission mechanisms, and context specific effects on aphid biology, virus epidemics depending on specific virus and aphid species involved [2]. Aphids transmit viruses from several families including *Potyviridae*, which is the largest plant infecting RNA virus family [6]. 

**Figure 1:**
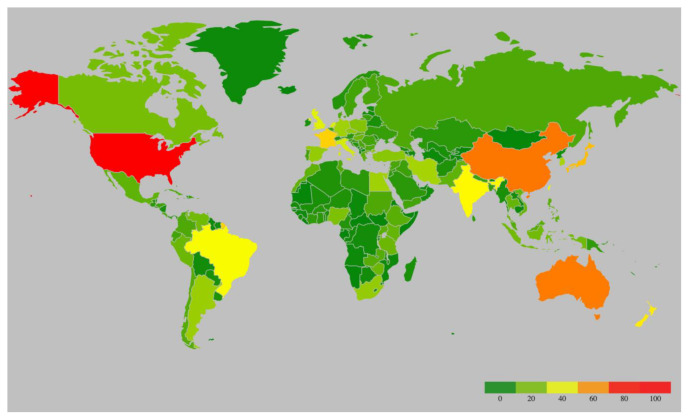
Heatmap of global distribution of potyviruses based on the scientific studies from Supplementary Table S1. Potyviruses have a cosmopolitan presence with the highest number of species recorded* in the United States (81), followed by China (63), Australia (62), France (49), New Zealand (45), India (43), and Brazil (41). *Recorded from exhaustive web search. The reporting and discovery of potyvirus species are not equivalent across the globe. Furthermore, species numbers are rapidly increasing with ongoing deep sequencing discovery.

Potyvirids, the members of virus family *Potyviridae*, are single-stranded, positive-sense RNA viruses [7,8]. A range of vectors transmits potyvirids through different transmission modes. For instance, the viruses from genera *Potyvirus* and *Macluravirus* are transmitted by aphids in a non-persistent manner; members of the genera *Rymovirus*, *Poacevirus* and *Tritimovirus* are transmitted by eriophyid mites in a semi-persistent manner; *Bymovirus* genus members are transmitted by a group of single-celled eukaryotes named Cercozoa; *Ipomovirus* genus members are transmitted by whiteflies, whereas the vectors of single-species genera *Brambyvirus*, *Bevemovirus* and *Roymovirus* are unknown [6]. Except for *Bymovirus*, all viruses within *Potyviridae* family have monopartite genomes [6]. With 176 described species, *Potyvirus* is the largest and the most extensively studied genus in *Potyviridae* family, mostly because of its economic importance [9]. Potyviruses have flexible and filamentous virions, 700–900 nm in length and 11–15 nm in width [10]. The majority of potyviruses have a narrow host range, but a few infect plant species in up to 38 host families causing significant losses in a wide range of crops [11]. They have a cosmopolitan distribution with the highest number of species recorded in the U.S., China, and Australia (Figure 1, Supplementary Table S1). Most potyviruses are estimated to be transmitted by over 200 species of aphids in a non-persistent, non-circulative manner as well as by mechanical inoculation [6]. 

This review presents an exclusive overview of aphid transmission of the *Potyvirus* genus including concepts and mechanisms underpinning transmission, virus-mediated effects on plant biochemistry and aphid behavior and biology, phylogenetic analyses of viral proteins involved in aphid transmission, transmission bottlenecks, and the interplay of an array of these factors on virus epidemics and aphid and virus management in agriculture. 

## 2. Non-Persistent Transmission

**Figure 2:**
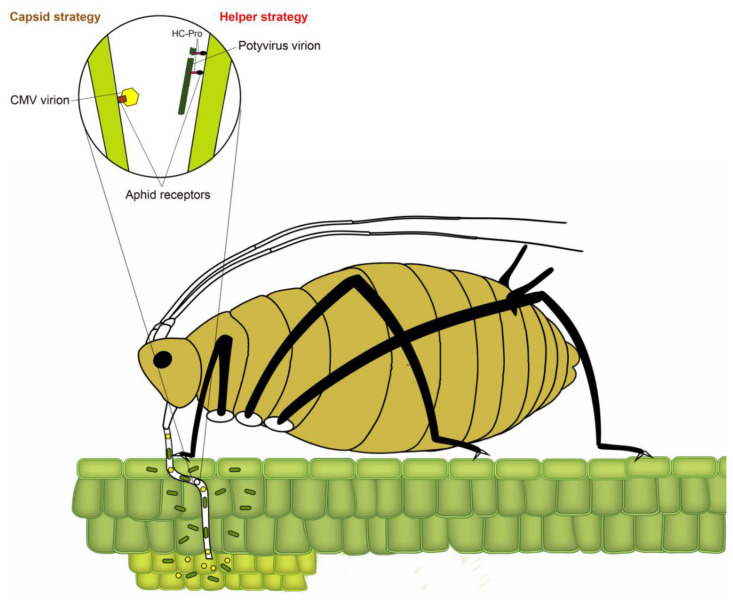
A model depicting two strategies of binding of non-persistent viruses to aphid receptors. The circle represents an enlarged view of the tip of the aphid stylet, showing virion binding through capsid and helper strategies. In capsid strategy (the left side of circle), amino acids in the coat protein of nonpersistent stylet-borne viruses such as Cucumber mosaic cucumovirus (CMV) bind to aphid receptors (e.g., Stylin-01 receptor of *Acyrthosiphon pisum* and *Myzus persicae*). In helper strategy (right side of the circle), helper component proteinase (HC-Pro) acts as a “molecular bridge” between coat protein of non-persistent potyviruses and aphid receptors, modified from Ng and Falk [13] and Whitfield et al. [20].

Transmission of plant viruses by insect vectors is categorized into four types: non-persistent; semi-persistent; persistent circulative and persistent propagative based on the virus localization in the vector, the time required by the vector for virus acquisition, retention and transmission and association of virus with various internal organs of the vector [4,12,13,14]. In non-persistent (stylet-borne) transmission, the vector requires seconds to few minutes to acquire virus, whereas, in semi-persistent (foregut-borne) transmission, the vector requires minutes to several hours for acquisition. Not all semi-persistent viruses are foregut-borne; a few such as *Carlavirus* and *Caulimovirus* are retained in the stylet tips [15]. Persistent transmission can be circulative or propagative depending on whether virus replicates inside insect vector. The vector requires several hours to a few days for acquisition of persistently transmitted viruses. Once acquired, a vector typically remains viruliferous for lifetime. Following the acquisition of non-persistently transmitted potyviruses, the aphid remains viruliferous only for a few feeding probes [16]. The non-persistent mode of virus transmission has been most widely studied in the *Potyvirus* type species *Potato virus* Y, with over 20 reported species of aphids capable of transmitting potato virus Y (PVY), including the ones incapable of colonizing potato [17,18]. In non-persistently and semi-persistently transmitted viruses, the binding of virions to aphid stylet or foregut has been conventionally described using two strategies (Figure 2) [19]. In the capsid strategy, coat protein (CP) directly interacts with binding sites (receptors) in the aphid stylet, whereas, in the helper strategy, additional non-structural protein (HC-Pro (helper component proteinase)) facilitates the binding by creating a reversible “molecular bridge” between CP and aphid receptors. 

## 3. Binding: Viral Proteins, Sites, and Aphid Factors

The potyviral RNA molecule of ~10 kb is polyadenylated and contains a single open reading frame (ORF) encoding a large polyprotein (340–368 kDa), which is eventually cleaved by viral encoded proteases into 10 functional proteins: P1 (protein 1 protease), HC-Pro; P3 (protein 3), 6K1 and 6K2 (six kilodalton peptides), CI (cytoplasmic inclusion), NIa-Pro (nuclear inclusion A protease), NIb (nuclear inclusion B/RNA-dependent RNA polymerase), CP (coat protein) [21]. A short pretty interesting potyvirus ORF (PIPO) is embedded within the P3-encoding region in a different reading frame than polyprotein [9]. The non-structural VPg (viral protein genome-linked) is covalently attached to the 5′-terminal [21]. Unlike capsid binding strategy, as seen in cucumoviruses such as cucumber mosaic virus (CMV), where CP alone binds to aphid stylet to facilitate aphid-mediated transmission [19], potyviruses require more intimate interactions between HC-Pro and CP for a successful binding with an aphid stylet [22] (Figure 2). The three partially overlapping regions of HC-Pro facilitate multiple functions including interactions between virions, plants and aphids, potyviral amplification, gene silencing suppression, systemic movement within plant, symptom development, and protease activity [23,24,25,26,27]. As one of the model strategies of non-persistence transmission, the role of HC-Pro in binding to aphid stylet has been described in detail in multiple comprehensive reviews [2,13,15,22]. Therefore, these topics have been covered in this review only in the context of aphid transmission of potyviruses.

**Figure 3:**
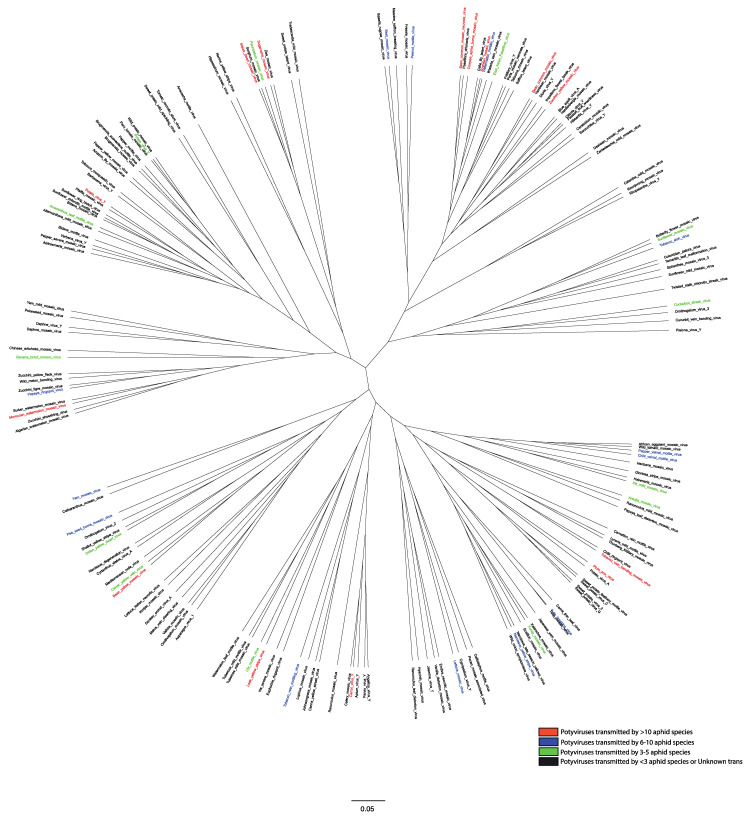
Phylogenetic analysis based on the coat protein (CP) sequences of 176 potyviruses. The genomic sequence for each exemplar potyvirus species was downloaded through NCBI GenBank. Accession numbers are provided in Supplementary Table S1. Nucleotide sequences were aligned using MAFFT v7.127b [37] and then subalignments for the CP coding regions were then manually refined by deleting highly gapped and ambiguously aligned sites. Phylogenetic trees for CP gene were then reconstructed in BEAST v 2.5.2 [38]. Sequences were assumed to evolve under a Hasegawa–Kishono–Yano (HKY) substitution model with gamma rate heterogeneity across sites. A strict molecular clock with a fixed substitution rate of 1.0 per unit time was assumed such that the resulting phylogenies have branch lengths given in units of substitutions per site. BEAST was run for 10 million Markov chain Monte Carlo (MCMC) iterations, sampling a tree from the posterior distribution every 10,000 iterations. The posterior distribution of trees was summarized as a Maximum Clade Credibility (MCC) tree with mean node heights. The resulting MCC tree for CP was then visualized in FigTree and uncertainty in the tree topology was assessed using the posterior support for each split (clade) in the tree. The potyvirus species in the tree was color-coded on the basis of number of aphid vectors from published scientific studies (Supplementary Table S1).

Genome-wide variation analysis revealed that potyviruses contain fixed hypervariable areas in key parts of the genome such as P1, HC-Pro, P3, VPg, NIb, CP, NIb-CP junction, and NIa protease [28]. These areas provide mutational robustness to potyviruses and appear to be potentially involved in host adaptation and some (CP and HC-Pro) in aphid transmission. Phylogenetic analysis on the CP sequences of 176 potyviruses revealed distinct grouping (Figure 3). Previous studies have shown that HC-Pro acts as a “molecular bridge” between virions and aphid stylets, which enables virion retention, direct interaction between CP and HC-Pro, and subsequent inoculations [22,29,30,31]. Mutations in either HC-Pro or CP of potyviruses have been shown to affect their transmissibility by aphids [32,33,34]. A highly conserved DAG motif in CP directly interacts with a PTK motif or its functionally equivalent motif/s located in the C-terminus of HC-Pro [34,35] to facilitate binding of HC-Pro to the coat protein of virions. Additionally, KITC motif and its functionally equivalent motifs in the N-terminus of the HC-Pro appear to be critical for virus retention in the stylets of aphid [33,35]. Both of these interactions between multiple amino acid motifs have been reported to be essential for the potyvirus transmission by aphids [30,34,36]. The members of the *Potyviridae* genera: *Rymovirus*, *Poacevirus* and *Tritimovirus* lacking all three amino acids motifs are not transmitted by aphids [6]. Instead, they are transmitted by eriophyid mites in a semi-persistent manner. In rose yellow mosaic virus (RoYMV), the type species of the monotypic genus *Roymovirus*, DAG motif of the CP is not found, and the potyvirus HC-Pro motifs PTK and KITC are substituted by C-2x-C motif at the N-terminus suggesting a possible eriophyid mite transmission [6]. In bellflower veinal mottle virus (BVMV), a member of the *Bevemovirus* genus, the DAG equivalent DTG motif is found near its CP N-terminus, but HC-Pro lacks PTK and KITC motifs essential for aphid transmission [6]. The vectors of both *Roymovirus* and *Bevemovirus* genera members remain unknown. 

**Figure 4:**
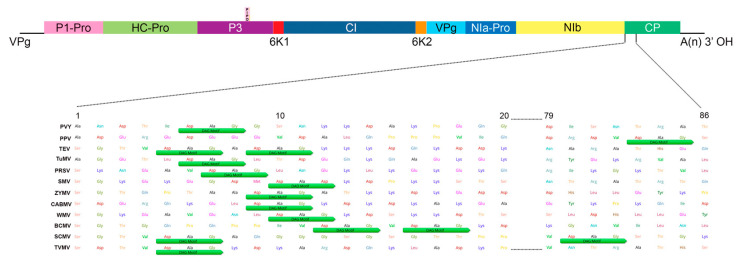
Variation in the position, adjacent amino acids, and number of DAG and DAG equivalent motifs in N-terminus of coat protein of 12 widely studied potyvirus species with regard to aphid transmission. DAG motif or its variants along with adjacent sequences are highly conserved in the *Potyvirus* genus and play important roles in molecular interactions with HC-Pro and aphid stylet receptors. The coat protein sequences were taken from the exemplar isolates of potyviruses specified on the International Committee on Taxonomy of Viruses (ICTV) website: potato virus Y (PVY) (U09509), plum pox virus (PPV) (AJ243957), tobacco etch virus (TEV) (M11458), turnip mosaic virus (TuMV) (AF169561), papaya ring spot virus (PRSV) (X67673), soybean mosaic virus (SMV) (D00507), zucchini yellow mosaic virus (ZYMV) (AF127929), cowpea aphid-borne mosaic virus (CABMV) (AF348210), watermelon mosaic virus (WMV) (AY437609), bean common mosaic virus (BCMV) (AJ312437), sugarcane mosaic virus (SCMV) (AJ297628), and the tobacco vein mottling virus (TVMV) (X04083).

Figure 4 shows the variability in N-terminus of coat protein of 12 widely studied potyviruses in the context to aphid transmission. The position of DAG motif and the composition of adjacent amino acids in N-terminus vary between potyviruses. Changes in both of these factors influence potyvirus transmissibility by aphids [31]. For instance, the tobacco etch virus (TEV) has two consecutive DAG motifs in its N-terminus at 5^th^ and 9^th^ amino acid positions separated by a single ala. The mutation in the first motif abolished aphid transmissibility, whereas that in the second motif did not [36]. However, the substitution of a single amino acid Ala with Val preceding second DAG motif restored transmissibility in a TEV mutant, which was made non-transmissible by an altered first DAG motif. Thus, in addition to the DAG motif itself, the amino acid preceding each motif appear to have a strong effect on aphid transmissibility of TEV. On the contrary, the addition of a second DAG motif at the 9^th^ position, which is naturally absent in tobacco vein mottling virus (TVMV) CP N-terminus and change in preceding amino acid (Val to Ala) in the single DAG motif, had little or no effect on aphid transmissibility [36]. Furthermore, seven amino acids, preceding and succeeding DAG motif (DTVDAGK) in the N-terminus of the TVMV CP, were reported to be involved in the aphid transmissibility of a series of TVMV variants and their ability to bind HC-Pro [30]. In sugarcane mosaic virus (SCMV), two DAG motifs, one at position 5 and the other at position 80 from the N-terminus, appear to be common in 77 of the 91 accessions analyzed by Nigam et al. [28]. In several potyviruses variants of DAG motif and its counterparts in HC-Pro play crucial roles in aphid transmissibility. Genome-wide analysis of potyviruses revealed that the most frequent deviations from DAG were NAG and NVG [28]. Other variants of DAG include DTG, DAE, DAA, GAG, DAD, and DAAA [28,39,40,41]. The point-mutant clones of soybean mosaic virus (SMV), in which DAG was replaced with DAD, showed no changes in aphid transmissibility [42].

A trans-complementation property of HC-Pro allows different forms of this protein to support the aphid-transmission of potyviruses as well as few viruses from different families [19]. For instance, the PVY was reported to mediate aphid transmission of the potexvirus, potato aucuba mosaic virus (PaMV), only in PaMV-PVY co-infected plants [43]. This is because of the presence of a domain including the DAG motif in the PaMV CP, which, in the presence of PVY, facilitated aphid transmission. Furthermore, potyvirus-dependent aphid transmissibility of potato virus X (PVX), a non-aphid-borne potexvirus, was achieved by cloning of the same domain into coat protein of PVX [43]. Multiple potyviruses and co-infecting viruses show that the interactions between HC-Pro and coat protein are conserved and are governed by the presence of specific sequence domains [41,44,45]. A few other factors that determine the aphid transmissibility of potyviruses include the structural and biochemical properties of HC-Pro [36,46] and availability of HC-Pro before or at the same time as virions in order for aphids to successfully transmit the virus [47]. Apart from the non-persistent aphid transmission of potyviruses, HC-Pro appears to play a key role in the semi-persistent transmission of the wheat streak mosaic virus (WSMV), a *Tritimovirus* transmitted by an eriophyid mite [48]. Whether engineered HC-Pro and coat protein of potyviruses facilitate semi-persistent and/or persistent transmission of other plant viruses remains to be seen. 

The interactions of HC-Pro and CP from the insect vectors’ perspective have remained largely unexplored. So far, only two studies have directly characterized the aphid receptors involved the binding of HC-Pro [31,49]. For a holistic understanding of potyvirus transmission by aphids, it is critical to identify and characterize putative receptors for viruses in aphid vectors. A network of highly cross-linked chitin fibers and cuticular proteins, and the difficulties in manipulating of insect stylets pose challenges to identifying and characterizing virus receptors in aphid vectors [50]. It is widely believed that putative potyvirus receptors are located on the extreme tip of maxillary stylets where the food and salivary canals fuse to form a common duct called ‘acrostyle’ [50,51]. Acrostyle was earlier shown to harbor receptors of cauliflower mosaic virus (CaMV), a non-persistently or semi-persistently transmitted (depending on the aphid species) DNA virus from the family *Caulimoviridae* [51,52]. Two cuticular proteins Stylin-01 and Stylin-02, which are highly conserved among aphids, were found to be localized in the acrostyle of the pea aphid *Acyrthosiphon pisum* and the green peach aphid *Myzus persicae*. Of which, Stylin-01 was reported as the first cuticular proteins involved in the aphid transmission of CaMV [50]. During CaMV infection, transmission bodies composed of aphid transmission complex are formed in infected plant cells, which, upon contact with aphids, are disintegrated releasing transmissible virions free into the cell [53]. Whether these receptor proteins or transmission bodies play any role in potyvirus transmission remains unclear. Furthermore, whether acrostyle harbors more receptors and, if so, their nature, specificity in aphid species and, for plant viruses, underlying genes for virus receptors and their precise interactions with viral proteins, remain unexplored. 

## 4. Aphid Biology: Behavior and Fitness

Plant viruses interact with their hosts and vectors at cellular and molecular levels, which further modulate host phenotype and vector behavior [15,54,55]. ‘Vector Manipulation Hypothesis’ suggests that a pathogen can directly (via presence of virions in the insect body) or indirectly (via altered infected-host phenotype) influence the insect vector behavior for their rapid transmission [56]. Potyviruses rely on aphids for horizontal plant-to-plant movement. Unlike persistent-circulative viruses, which tend to have a long-term intimate relationship with their vectors, the non-persistent viruses are presumed to be transiently associated with their vectors with their interactions being short-lived [2,13,15,54,55]. 

**Figure 5:**
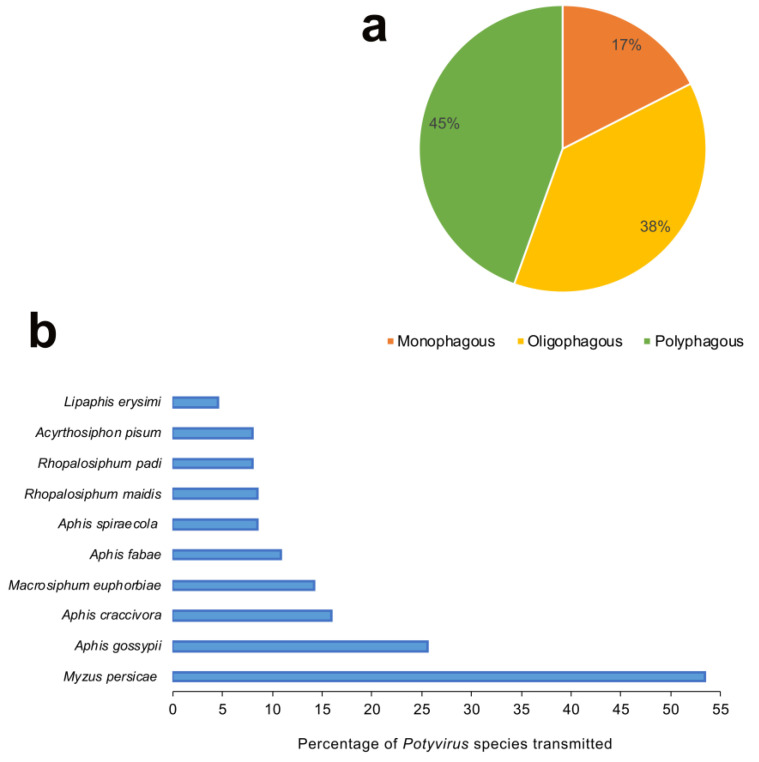
(**a**) Categorization of aphid vectors of potyviruses based on their host plant range. The majority of aphid species (45%) are polyphagous, followed by about a third (38%) oligophagous and a few (17%) monophagous by feeding nature; (**b**) top 10 aphid vectors of potyviruses based on the number of potyviruses transmitted as specified in the Supplementary Table S1. The green peach aphid *Myzus persicae* transmitted the highest percentage of total 176 potyviruses (53.4%), followed by melon aphid *Aphis gossypii* (25.6%), cowpea aphid *Aphis craccivora* (15.9%), potato aphid *Macrosiphum euphorbiae* (14.2%) and black bean aphid *Aphis fabae* (10.8%). The majority of aphid species transmitted less than three potyvirus species.

The majority of 176 potyviruses have been reported to be transmitted by less than three aphid vectors or have no known vectors (Figure 3). The feeding nature of aphids (generalist vs. specialist) is one of the key determinants of the success of aphids as a vector. The meta-analysis of 137 aphid species revealed that 45% of aphids were polyphagous (feeding on plants from multiple families), 38% were oligophagous (mostly feeding on plants from multiple genera within the same plant family) and a mere 17% were monophagous (mostly feeding on plant/s from the same genus and family) by nature (Figure 5a). We ranked the top 10 aphid vectors based on the percentage of total 176 potyviruses they transmit. With 53.4% of 176 potyviruses transmitted, *M. persicae* was by far the most efficient and/or widely studied vector of potyviruses (Figure 5b). It is most likely due to the highly polyphagous nature, more widespread studies, high economic importance, and cosmopolitan distribution of *M. persicae*. The remaining aphid vector species include *Aphis gossypii* (25.6%), *Aphis craccivora* (15.9%), *Macrosiphum euphorbiae* (14.2%), *Aphis fabae* (10.8%), *Aphis spiraecola* (8.5%), *Rhopalosiphum maidis* (8.5%), *Rhopalosiphum padi* (8%), *A. pisum* (8%), and *Lipaphis erysimi* (4.5%). The 7 of top 10 aphid vectors, except *R. maidis*, *R. padi* and *L. erysimi*, were polyphagous in nature. Thus, it is plausible that the increased access to wide range of host plants and by extension potyviruses would lead to aphids transmitting multiple potyviruses with varying efficiencies and employing various strategies for virus spread. It is plausible that the majority of aphid species remain unexplored and unreported in terms of their potyvirus transmission ability. Particularly, non-colonizing aphid species that may land and leave the crop immediately after probing, thus primarily acting as transient vectors. In addition, since a vast majority of aphid species, in general, are not polyphagous [57], it is more likely that they just move potyviruses from one host plant to another in the process of exploratory probing when searching for the primary host plants.

The complexity of vector–virus interactions suggests long-term co-evolution of plant viruses with vectors and host species [58]. Different aphid species transmit different potyviruses with varying transmission efficiencies. This is most likely due to a number of ecological and evolutionary factors shaping pathosystem-specific outcomes on virus transmission and spread. Non-persistent viruses are not known to be as intimately associated with their vectors as persistent ones [59]. However, the shared host plants and prevalence of different potyvirus strains and the variation in aphid transmission efficiency primarily hint at the possibility of a co-evolution of aphid vectors and potyviruses they transmit over a significant period of time. The degree of specialism of aphid also appears to determine the transmission efficiency of aphid vectors in a context specific manner. For instance, the generalist *M. persicae* was reported to consistently transmit PVY to pepper (*Capsicum annuum*) and plum pox virus (PPV) to *Nicotiana benthamiana* with higher efficiency than the specialist mealy plum aphid *Hyalopterus pruni* [60]. On the contrary, Adachi et al. [61] showed that the relationship between turnip mosaic virus (TuMV) and its specialist aphid vector *L. erysimi* was more mutualistic than its generalist aphid vector *M. persicae*, regardless of the phylogenetic differences in TuMV strains. Furthermore, Gadhave et al. [62] reported that papaya ring spot virus (PRSV) offers selective fitness benefits to its vector *A. gossypii* over the non-vector whitefly *Bemisia tabaci* despite both insects sharing the same feeding guild. Potyviruses tend to discriminate between their multiple vectors, in that they offer selective benefits to some vectors over the others [61,63] and to vectors over non-vector insects [62]. 

Transmission efficiency and populations of aphid vectors variably contribute to the success of virus transmission and spread. Most efficient aphid species may not always contribute the most to the virus epidemics. For instance, several studies have reported relatively less efficient aphid vectors: *A. pisum*, *A. fabae*, *R. padi*, *Aphis nasturtii*, *A. gossypii*, *M. euphorbiae*, *L. erysimi* and *Brachycaudus helichrysi* have been more important to PVY epidemiology than the most efficient vector of PVY: *M. persicae* [18,64,65,66,67,68]. The less efficient vectors appear to make up for the efficiency with their high populations, early plant colonization, and the ability to rapidly develop winged forms. There seems to be strain-specific variations in the ability of aphids to transmit multiple potyvirus strains. For instance, *M. persicae* was reported to simultaneously transmit multiple strains of PVY: PVY^O^, PVY^NTN^, and PVY^N:O^, with varying transmission efficiencies of certain strains and combinations [69,70,71]. The observed strain-specificity was thought to be responsible for the increased incidence of PVY^NTN^ and other necrotic strains of PVY over non-necrotic ones [71].

The aphid transmission efficiency of a few potyviruses also seems to be dependent on the colonizing behavior of vector species [72,73,74,75]. Aphid species that are non-colonizers and engage in rapid intracellular puncturing after landing on the host plants appear to be better transmitters for potyviruses than colonizers [60,75,76,77,78]. For instance, Raccah et al. [47] reported that non-colonizers such as *Aphis citricola* and *Aphis* spp. contribute more to the total transmission of CMV and PVY to pepper crops than colonizers such as *M. euphorbiae* and *M. persicae*. In some instances, colonizing aphids such as *M. persicae*, *M. euphorbiae*, and *A. nasturtii* reported to be better transmitters of PVY than three casual visitors to potato: *Myzus cerasi*, *R. padi*, and *Sitobion avenae* [79]. However, both colonizing and non-colonizing aphid species reported to be important to overall transmission and spread of certain potyviruses. For instance, cowpea aphid-borne mosaic potyvirus (CAMV) transmission appeared to be dependent on multiple colonizing as well as non-colonizing aphid species [80], although colonizing aphid species reported to be more efficient at the secondary spread of the virus.

To date, the effects of non-persistent viruses on their vector behavior and fitness have been studied only in a few select pathosystems (Figure 3). The electrical penetration graph (EPG) reads the electrical waveforms associated with aphid feeding and behavior. These include: non-probing (np), intracellular stylet puncture (pd), intercellular stylet pathway (C), salivation into phloem sieve elements (E1), and passive phloem sap uptake from phloem sieve elements (E2) [81,82,83]. Since potyvirus infection is not limited to any particular plant tissue [84], potyviruses appear to be most frequently acquired and inoculated during intracellular stylet puncture (pd) phase [81,85]. Previous EPG studies showed that aphids acquire non-persistent viruses within 3–5 s of stylet penetration into the epidermal cells [85,86,87] and lose their ability to transmit them within minutes of their removal from infected plants [2]. Thus, aphid probing appears to be one of the factors contributing to the variability in potyvirus transmission between different aphid species [88]. For instance, Boquel et al. [63], using EPG, demonstrated that PVY differentially modulates the feeding behavior of its two vectors: the potato aphid *M. euphorbiae* and *M. persicae*, which differ in their biology and PVY transmission ability. *M. persicae*, being a sedentary species but an excellent vector of PVY, showed reduced non-probing duration, increased phloem sap ingestion, and increased arrestment on PVY-infected plants. In contrast, *M. euphorbiae*, being a mobile species but not a very good vector of PVY, showed delayed stylet insertion, reduced activity in the phloem vessels, and an enhanced non-probing duration. 

The role of host plant molecules in mediating non-persistent virus-vector interactions has been relatively well-studied [89,90]. Such interactions are influenced by altered host cues for the vector and/or enhanced host plant nutritive quality. Olfactory host cues are typically modulated through altered plant volatile organic compounds (VOCs) leading to the movement of vectors towards (immigration) or away from (emigration) virus-infected plants. Studies on vector preference manipulation by potyviruses have reported result from across the spectrum, which appear to be context specific. For example, Eigenbrode et al. [91] reported that PVY increased the arrestment of *M. persicae*, with significantly lesser emigration from the headspace of PVY-infected plants compared to the non-infected ones. Furthermore, Eckel [92] reported that tobacco infected with TEV attracted more aphid compared to non-infected plants. In contrast, Boquel et al. [63] reported the reduced *M. euphorbiae* preference to PVY-infected potato compared to non-infected plants. The results from dual vector-single virus and from mixed-virus-single vector pathosystems also show context specificity in aphid transmission. For example, two aphid vectors of SMV showed contrasting behaviors, in that *M. persicae* showed no preference to either SMV-infected or non-infected soybean plants, whereas the corn leaf aphid *R. maidis* showed increased arrestment on non-infected plants [93]. In the mixed plant infections, potyviruses appear to interact with each other vis-à-vis vector for an efficient transmission. For example, two potyviruses: watermelon mosaic virus (WMV) and zucchini yellow mosaic virus (ZYMV) co-infecting squash manipulated their common aphid vector *A. gossypii* (melon aphid) differently than in single infections [94]. Despite less accumulation of WMV in squash than ZYMV because of antagonistic interactions, WMV benefited from co-infection due to its increased transmission by *A. gossypii*. 

In addition to VOCs, potyviruses are reported to modify the vector fitness by making plants more or less nutritious [95,96,97]. Multiple studies have reported the increased fitness of aphid vector on plants infected by potyviruses. For instance, *A. gossypii* showed increased longevity and higher fecundity on ZYMV-infected squash [98] and the mustard aphid *L. erysimi* showed higher population growth on TuMV-infected plants than the non-infected ones [61]. Similarly, *M. persicae* showed higher growth, reproduction, and survival on PVY-infected potato [96] and TuMV-infected *N. benthamiana* plants than on non-infected plants [99,100]. Potyvirus-mediated host-vector interactions are partly governed by altered host-plant gene expression profiles involved in the biosynthesis of olfactory and gustatory cues [97,99,100,101]. For instance, PVY-infected tomatoes trigger salicylate induction resulting in the increased abundance and fecundity of the aphid *M. euphorbiae* [102]. Bak et al. [101] reported that ethylene signaling by PVY triggered aphid attraction to PVY-infected plants and virus spread. The gene expression system influenced by phytohormone are wide, overlapping, and complex. Therefore, careful elucidation of gene expression profiles in host plant is required to fully understand and identity the genes responsible for potyvirus-led modulation of host and vector interactions. 

So far, only a few studies have dissected the direct roles of viral proteins in manipulating aphid biology. For instance, the expression of PVY HC-Pro in transgenic *N. benthamiana* was reported to enhance the growth of vector *M. persicae* by suppressing jasmonic acid (JA)-regulated signaling, which is considered critical to induce plant resistance against herbivory [97]. TuMV NIa-Pro protein was reported to alter the physiology of *N. benthamiana* by suppressing callose deposition and by increasing the abundance of free amino acids leading to increased *M. persicae* arrestment and reproduction on TuMV infected plants [99]. Furthermore, potyviruses: TuMV and PVY have been reported to respond to the presence of their vectors and promote insect vector performance and transmission only when needed. This response was mediated via localization of NIa-Pro to vacuoles only when aphids are present [103]. 

## 5. Virus Epidemics

Aphid attraction, arrestment, and dispersal from potyvirus infected plants are very crucial for virus spread. Aphids use an array of visual, volatile, and gustatory cues to find potyvirus infected plants. Non-persistent (NP) viruses such as potyviruses are presumed to be transiently associated with their vectors, especially since their transmission requires very short acquisition and transmission times. The best case scenario for potyvirus spread, therefore, is thought to be a rapid attraction of aphid vectors by a potyvirus infected plant, quick acquisition of virus by aphids and rapid spread of the potyvirus to non-infected plants [58,104,105]. For instance, a non-persistent *Cucumovirus* CMV has been reported to induce specific biochemical changes in a plant host that modify the alighting, settling, and probing behaviors, and fitness of its vectors *A. gossypii* and *M. persicae* [106,107]. The biochemical changes in host plants include reduced host-plant quality for aphids causing rapid vector dispersal, reduced carbohydrates and amino acids in leaf tissue and phloem, and changes in plant stress hormones. However, a few previous studies show that this is not always the case; in fact, the opposite is true in certain pathosystems. For instance, potyviruses PVY, TuMV, ZYMV, WMV, and PRSV reported to have context-specific effects on aphid behavior and fitness depending on the aphid species, infection status, and host plants [62,63,79,94,99,100]. Contrary to the popular belief, PRSV and TuMV, in particular, appear to increase the fitness of their vectors *A. gossypii* and *M. persicae*, respectively. Certain potyviruses could use the increased fitness of their vectors to facilitate quick vector population build up, rapid wing development and subsequently increased virus spread. Therefore, establishing large number of inoculum foci over greater distances than the rapid and fewer inoculum foci due to quick vector dispersal. More studies on different pathosystems involving potyviruses would be helpful to draw generic patterns on how potyviruses manipulate their vectors to encourage virus spread.

A winged (alate) form of aphids are important than wingless (apterate) forms for the spread of potyviruses in the field [108,109]. Winged aphids are responsible for establishing inoculum foci and secondary spread thereafter in the farmscapes [109]. Winged forms are typically produced in search of new hosts because of food source depletion and overcrowding [110]. On the contrary, wingless forms are produced when conditions are favorable throughout summer, without significant movement between plants, rendering them insignificant vectors of potyviruses [109,110]. For instance, field studies on PVY revealed a good correlation between the number of winged aphids and spread of potato virus Y^O^ [67,68]. Furthermore, the dispersal distance analysis suggests that PPV-infected aphids preferentially spread PPV beyond 90 m, i.e., away from infected trees, rather than to neighboring trees—thus subsequently encouraging the secondary spread of PPV over large orchard landscapes [111]. Field surveys in Japan showed a peak of PPV-viruliferous winged aphids occur in fall, when a catch in aphid traps are smaller compared to spring and summer [112]. This could be attributed to the overall increase in the number of winged aphids feeding on PPV-infected prunes and/or the enhanced movement of viruliferous aphids over the non-viruliferous ones in the fall. Since virus spread is the function of number of vector visits per plant per day [113,114], PPV mediated enhanced movement of viruliferous aphids may be a key factor in driving the virus spread. 

## 6. Transmission Bottlenecks

Transmission bottlenecks for viruses occur during the initiation of a new infection when only a few virions are transmitted from one infected host to another [115]. A very few studies have assessed the transmission bottlenecks for plant viruses transmitted non-persistently or otherwise by insect vectors. Being non-persistently transmitted, virions of potyviruses are not translocated to the haemolymph and are expelled out with saliva during feeding [81,116,117]. Depending upon experiment design and pathosystems involved, different studies have reported a wide range of viral RNA targets or virions inoculated by aphids during virus transmission by aphids. For instance, individual *M. persicae* aphids, when allowed a 10 min acquisition access period, were reported to acquire between 15 to 20,760 TEV or TVMV particles when successfully infecting new plants [118]. On the contrary, Moury et al. [119] reported this number to be very low (average 0.5–3.2 virus particles) when estimating the size of bottlenecks during aphid transmission of two PVY variants. The huge discrepancy in these two studies was thought to be due to differences in methods followed and due to the overestimation of the number of transmitted virions in the former study including the ones that were ingested and remained unviable. A more recent study by Moreno et al. [120] took a different approach, in that they used viral RNA targets instead of virions as a measure of transmission bottlenecks. The EPG and TaqMan real time PCR (RT-PCR) revealed that *M. persicae* lose approximately half of the average number of acquired PPV RNA targets in a single probe (26,750) while infecting Japanese plum trees with PPV. More studies on potyvirus transmission bottlenecks are required to assess their effects on the fitness and virulence of potyviruses and their transmission by aphids. 

## 7. Management

### 7.1. Current Measures

Devising effective virus and vector management tools and strategies requires a deeper understanding of viruses, vectors, and plants, and their underlying component and community interactions. The key challenge of managing aphids as pests is to keep the populations of wingless forms low, whereas that of managing potyvirus spread is to prevent the formation of winged forms or to kill them before they infect healthy plants [108]. The use of pesticides is not considered an ideal strategy to mitigate non-persistent virus epidemics because of the short time aphids need to transmit potyviruses [121,122,123]. For instance, several studies reported that the use of insecticides have a low impact on the spread of PVY as aphids transmit PVY prior to being killed by insecticides [124,125,126,127]. Furthermore, a single winged aphid, with the very brief probing activity, is capable of transmitting one or more strains of potyviruses such as PPV in the field conditions [122]. Therefore, the failure of insecticides to wipe out the entire aphid population and the rapid escape of winged forms from insecticide treated plots also make insecticides a very ineffective method of aphid and virus management in the field conditions. On the contrary, the integration of several approaches has been proven to be effective strategy for potyvirus and aphid management. For instance, the use of virus-free planting material, PPV-resistant cultivars, and physical barriers, and the removal of PPV inoculum sources including overwintering hosts appear to be effective and efficient strategies for PPV management over the insecticide treatments [122]. Similarly, the use of oil spraying, straw mulching, rouging, and intercropping as an integrated strategy proved to be effective against PVY than insecticides for vector and virus management [123]. The use of barrier crops has been proved to be effective to control multiple potyviruses such as chilli vein mottle virus (CVMV), PVY, bean common mosaic virus(BCMV), bean yellow mosaic virus (BYMV), SMV, and maize dwarf mosaic virus (MDMV) in a wide range of crops [128]. Reflective mulches applied at the time of cucurbit planting have been shown to be effective in repelling aphids from plants, thereby reducing the incidence of WMV, PRSV, and ZYMV, potyviruses commonly occurring in the U.S. farmscapes [129,130]. Furthermore, the use of mineral oil individually [131,132] and in combination with other treatments such as reflective mulches [133] and crop borders [134] has been proved to be effective in the management of multiple potyviruses such as PVY and PRSV. Earlier studies report that mineral oil modifies the feeding behavior of aphids [135] and interferes with the binding of potyvirus virions to aphid stylets [132], making it one of the effective strategies for potyvirus management. 

Breeding of resistant cultivars is also considered to be one of the best strategies to manage diseases caused by aphid-transmitted potyviruses. For non-virus plant pathogens, natural resistance is predominantly inherited by monogenic dominant characters [136,137]. However, for plant viruses, including and especially potyviruses, natural recessive resistance appears to be more common, and conferred to plants by a mutation in a recessive gene that codes for a host of factors critical for viral replication [138]. Eukaryotic translation initiation factor (eIF) 4E and eIF4G and their isoforms are the most commonly used recessive resistance genes. eIF4Es-mediated resistance against potyviruses such as PVY and lettuce mosaic virus (LMV) has been exploited in several resistant crop cultivars of pepper, lettuce, and tomato [139,140,141]. Several transgenic cultivars of select agricultural crops have been developed over the years—using multiple strategies—in an effort to tackle a number of economically important potyviruses. Initial attempts to achieve PVY resistance in potato were based on the ectopic expression of multiple viral proteins such as CP, NIa, Nib, and P1 [142,143,144,145]. The CP-mediated resistance (CPMR) has been extensively used against multiple potyviruses with mixed success [142,146,147,148,149,150,151,152]. For instance, transgenic cultivars expressing PVX and PVY CP reported to offer variable degree of resistance against mechanical and aphid transmission of PVY [147,148]. The expression of the PRSV CP gene in tobacco offered protection against infection by a broad spectrum of potyviruses such as TEV, PVY, and pepper mottle virus (PeMV) [153]. Transgenic potato Bt6 expressing PVY CP gene provided resistance to primary and secondary infections by PVY when transmitted by aphids [142]. In Hawaii, transgenic papaya cultivars “SunUp” and “Rainbow” carrying the CP of mild PRSV strain HA 5-1 saved the commercial papaya industry, when other methods of PRSV control failed [154]. Similarly, transgenic plum clone C5 (cv. HoneySweet) demonstrated the high level of resistance to PPV infection by graft inoculation or natural infection through aphid vectors [155]. Overall, the resistance achieved via ectopic expression appear to be variable from mild to strong [147,148], partial [145] or strain-specific [143,146], with the varying degree of success depending on the pathosystem. To date, transgenic cultivars have been the most promising approach of managing potyvirus infection and aphid transmission in a very few crops. However, the breakdown of viral resistance remains a challenge as potyviruses have a high rate of viral mutation and recombination [156,157].

### 7.2. Future Directions

The multi-omics approaches such as RNA interference (RNAi) and CRISPR gene editing are being used to develop efficient, eco-friendly, and sustainable virus and vector management strategies. RNAi plays an important role in plant defense against plant pathogens by degrading target RNA molecules at transcriptional (Transcriptional Gene Silencing, TGS) or at post-transcriptional levels (Post-Transcriptional Gene Silencing, PTGS) [158,159]. In an effort to manage potyviruses and consequently their aphid transmission, multiple RNAi-inducing constructs have been developed including PPV CP [160], SMV P3 [161], sorghum mosaic virus (SrMV) CP [162], and cowpea aphid-borne mosaic virus (CABMV) CP [152]. A recent study by Worrall et al. [163] reported that topical application of dsRNAs targeting either NIb or CP protected *N. benthamiana* and cowpea plants from aphid-mediated transmission of BCMV. This study hints at the possibility of using RNAi for crop protection. However, the key issues of variability, incompleteness of knockdown and off-target effects need to be addressed for the successful application of RNAi in field conditions [164]. 

CRISPR gene editing has tremendous potential in engineering resistance against plant viruses. This technology can be used to develop tools that can genetically manipulate any and all components of insect transmission cycle: viruses, insect vectors, and plants to achieve plant genetic improvement and protection within a short span of time. A recently developed CRISPR/Cas13a tool [165] is already showing a promise in conferring resistance against potyviruses. For instance, Aman et al. [166] successfully developed CRISPR/Cas13a constructs expressing HC-Pro and green-fluorescent protein (GFP) guide-RNAs (gRNAs) and showed the efficient and specific virus interference, triggering TuMV resistance in *N. benthamiana*. This study demonstrates the dual benefit of using CRISPR/Cas13a system: conferring resistance to RNA plant viruses by specifically targeting any RNA transcript and, most importantly, blocking the aphid transmission of potyviruses by successful knocking down of HC-Pro. Zhan et al. [167] recently developed transgenic potato lines expressing Cas13a/sgRNA (small guide RNA) constructs, which suppressed PVY accumulation and disease symptoms. Other successful attempts involve engineering transgene-free genetic resistance to potyviruses: TuMV [168] and clover yellow vein virus (ClYVV) [169] in *Arabidopsis* and cassava brown streak virus (CBSV) in cassava [170], using CRISPR-based technologies specifically targeting eIF4E, a eukaryotic translation initiation factor. The development of CRISPR engineered crops with increased resistance to viral diseases would ultimately be effective to suppress the primary and secondary spread of potyviruses via aphids. 

The CRISPR-Cas9 system is also proving to be very efficient in editing genomes of numerous insects through microinjection of eggs with guide RNAs targeting genes of interest. Since aphids have a complex life cycle with dual modes of reproduction, the genome editing of aphids is very challenging [171]. A successful attempt has recently been made to tailor CRISPR-Cas9 procedure to the pea aphid *A. pisum*, which is involved in the microinjection of fertilized eggs with CRISPR-Cas9 components specifically targeting *Stylin-01*, a cuticular protein gene [171]. However, alternate approaches to aphid gene editing need to be explored and a fine-tuning of the current procedure is required as it is 7-months long and tedious. Since Stylin-01 plays a key role in the aphid transmission of CaMV, a non-persistent caulimovirus [50], whether its editing would affect CaMV and by extension potyviral transmission via aphids remains to be seen. The development of sustainable management solutions from novel approaches such as RNAi and CRISPR gene editing will be challenging, especially because of the field adoptability, efficacy and efficiency constraints, and regulatory, containment, and bioethical issues. 

## 8. Conclusions

Potyviruses are the largest group of plant-infecting RNA viruses that, together with their aphid vectors, cause substantial agricultural crop losses throughout the world. Both potyviruses and their vectors share common features such as plant hosts, agricultural niches, and worldwide distribution, and pose a serious threat to global food security. Unlike persistently transmitted viruses, vector–virus interactions and their management have been studied only in a select virus pathosystems including PVY, TEV, SMV, WMV, ZYMV, TuMV, PPV, and PRSV, while the majority of 176 potyviral species remain sparsely studied. Potyviruses exercise pathosystem-specific, yet direct effects on their aphid vectors, which are often indirectly manipulated via host plants. These include the altered aphid probing, host plant cues (e.g., volatiles) and nutritional quality ultimately triggering aphid behavior and fitness. Two strategies by which potyviruses encourage their transmission and spread appears to be (a) ‘quick dispersion-low nutrition’ strategy based on rapid attraction and dispersion of aphids from less-nutritious potyvirus infected plants and (b) ‘slow dispersion-high nutrition’ strategy based on increased arrestment on highly-nutritious potyvirus infected plants facilitating rapid wing development and subsequently increased virus spread. Plant virus transmission being a multi-trophic event, an array of virus, insect, and plant factors determine the fate of aphid transmission of potyviruses. These include viral factors especially CP and HC-Pro, aphid factors such as probing, generalist vs. specialist feeding nature, colonizing vs. non-colonizing habits, and winged vs. wingless forms, and plant factors such as plant volatile, amino acid, and carbohydrate profiles that directly influence aphid biology. Over the years, diverse approaches such as conventional breeding, cultural practices, agro-chemicals, and transgenic cultivars have been used to manage potyviruses and their aphid vectors. However, the dual damage by virus and aphids, high rate of virus mutation and recombination, and lack of robust control measures, especially against viruses, pose major challenges to their management in field settings. The use of integrated virus and vector management strategies appear to be a sustainable and effective approach to tackling some of these challenges. Multi-omics approaches such as RNAi and CRISPR-based gene editing techniques are enabling the fundamental understanding of the mechanisms plant viruses and aphids employ to maintain their intimate and evolutionary relationship. This understanding will open new avenues for future crop improvement and protection strategies. 

## Supplementary Materials

The following are available online at https://www.mdpi.com/1999-4915/12/7/773/s1, **Table S1:** Geographical distribution and aphid vectors of potyviruses.
