# Supplementary material for: Aphid Transmission of Potyvirus: The Largest Plant-Infecting RNA Virus Genus
Source: Viruses. 2020 Jul 17;12(7):773. doi: 10.3390/v12070773 (PMC7411817; doi:10.3390/v12070773)
Supplement: Supplementary file 1 [file viruses-12-00773-s001.pdf]

viruses

MDPI

Supplementary

Aphid Transmission of *Potyvirus*: The Largest Plant-Infecting RNA Virus Genus

Kiran R. Gadhave [1,2,*,†], Saurabh Gautam [3,†], David A. Rasmussen [2] and Rajagopalbabu Srinivasan [3]

[1] Department of Plant Pathology and Microbiology, University of California, Riverside, CA 92521, USA
[2] Department of Entomology and Plant Pathology, North Carolina State University, Raleigh, NC 27606, USA; drasmus@ncsu.edu
[3] Department of Entomology, University of Georgia, 1109 Experiment Street, Griffin, GA 30223, USA; babusri@uga.edu
* Correspondence: kiran.tnau@gmail.com.
† Authors contributed equally.

Received: 13 May 2020; Accepted: 15 July 2020; Published: date

Abstract: Potyviruses are the largest group of plant infecting RNA viruses that cause significant losses in a wide range of crops across the globe. The majority of viruses in the genus *Potyvirus* are transmitted by aphids in a non-persistent, non-circulative manner and have been extensively studied vis-à-vis their structure, taxonomy, evolution, diagnosis, transmission and molecular interactions with hosts. This comprehensive review exclusively discusses potyviruses and their transmission by aphid vectors, specifically in the light of several virus, aphid and plant factors, and how their interplay influences potyviral binding in aphids, aphid behavior and fitness, host plant biochemistry, virus epidemics, and transmission bottlenecks. We present the heatmap of the global distribution of potyvirus species, variation in the potyviral coat protein gene, and top aphid vectors of potyviruses. Lastly, we examine how the fundamental understanding of these multi-partite interactions through multi-omics approaches is already contributing to, and can have future implications for, devising effective and sustainable management strategies against aphid-transmitted potyviruses to global agriculture.

Keywords: potyviruses; vector-virus interactions; aphid transmission; plant viruses; insect vectors

Supplementary Table S1. Geographical distribution and aphid vectors of potyviruses.

Species	Accession number	Country	References	Aphid species	References
African eggplant mosaic virus	MF997470	Tanzania	Tanzania (Unpublished, Genbank Acc. No. NC_043537)	Unknown	
Algerian watermelon mosaic virus	EU410442	Algeria	Yakoubi, S.; Lecoq, H.; Desbiez, C. Algerian watermelon mosaic virus (AWMV): A new potyvirus species in the PRSV cluster. *Virus Genes* **2008**, *37*, 103–109, doi:10.1007/s11262-008-0237-x.	Unknown	
Alstroemeria mosaic virus	AB158522	Taiwan, Netherlands, United Kingdom (UK), Mexico, New Zealand, Japan	Wang, C.Y.; Chang, Y.C. First identification of alstroemeria mosaic virus in Taiwan. *Plant Pathol*. **2006**, *55*, 566, doi:10.1111/j.1365-3059.2006.01425.x.<br>Van Zaayen, A.; De Blank, C.M.; Bouwen, I. Differentiation between two potyviruses in alstroemeria. *Eur. J. Plant Pathol.* **1994**, *100*, 85–90, doi:10.1007/BF01871969.<br>Phillips, S.; Brunt, A.A. Four viruses of alstroemeria in Britain. *Acta. Hortic.* **1986**, *177*, 227–234, doi:10.17660/ActaHortic.1986.177.30.<br>Gutiérrez-Estrada, A.; Zavaleta-Mejía, E.; Gaytán-Acuña, E.A.; Herrera-Guadarrama, A.J. Virus associated with alstroemeria in México. *Rev. Mex. Fitopatol.* **2000** ,*17*, 97–103.<br>Pearson, M.N.; Cohen, D.; Cowell, S.; Blouin, A.; Lebas, B.S.M.; Shiller, J.B.; Clover, G.R.G. A survey of viruses of flower bulbs in New Zealand. **2009**, *38*, 305–309, doi:10.1071/AP09006.<br>Fuji S.I.; Mochizuki, N.; Fujinaga, M.; Ikeda, M.; Shinoda, K.; Uematsu, S.; Furuya, H.; Naito, H.; Fukumoto, F. Incidence of viruses in alstroemeria plants cultivated in Japan and characterization of broad bean wilt virus-2, cucumber mosaic virus and youcai mosaic virus. *J. Gen. Plant Pathol.* **2007**, *73*, 216–221, doi:10.1007/s10327-007-0009-9.	*Myzus persicae*, *Neotoxoptera formosana*	Yasuda, S.; Saka, K.; Natsuaki, K.T. Characterization and serodiagnosis of alstroemeria mosaic potyvirus. *Jpn. J. Trop. Agric.* **1998**, *42*, 85–93, doi: 10.11248/jsta1957.42.85.
Alternanthera mild mosaic virus	EF442668	Brazil	Almeida, A.M.R.; Fukushigue, C.Y.; Sartori, F.; Binneck, E.; Marin, S.R.R.; Inoue-nagata, A.K.; Chagas, C.M.; Souto, E.R.; Mituti, T. Natural infection of *Alternanthera tenella* (Amaranthaceae) by a new potyvirus. *Arch. Virol.* **2007**, *152*, 2095-2099, doi:10.1007/s00705-007-1036-8.	*M. persicae*	Almeida, A.M.R.; Fukushigue, C.Y.; Sartori, F.; Binneck, E.; Marin, S.R.R.; Inoue-nagata, A.K.; Chagas, C.M.; Souto, E.R.; Mituti, T. Natural infection of *Alternanthera tenella* (Amaranthaceae) by a new potyvirus. *Arch. Virol.* **2007**, *152*, 2095-2099.
Amaranthus leaf mottle virus	AJ580095	Italy, Spain, Morocco	Segundo, E.; Lesemann, D.E.; Martín, G.; Carmona, M.; Ruiz, L.; Cuadrado, I.M.; Velasco, L.; Janssen, D. Amaranthus leaf mottle virus: 3'-end RNA sequence proves classification as distinct virus and reveals affinities within the genus Potyvirus. *Eur. J. Plant Pathol.* **2007**, *117*, 81–87, doi:10.1007/s10658-006-9064-x.<br>Lisa, V.; Lecoq, H. Zucchini yellow mosaic virus. CMI/AAB Descriptions of Plant Viruses, 1984, no. 282. Kew, Surrey.<br>Casetta, A.; D'Agostino, G.; Conti, M. Isolamento di ''amaranthus leaf mottle virus'' (ALMV) da Cirsium arvense. *Informatore Fitopatologico*, **1986**, *36*, 43–86, ISSN 0020-0735.	*Myzus persicae*, *Aphis gossypii*, *Macrosiphum*, *euphorbiae*	Segundo, E.; Lesemann, D.E.; Martín, G.; Carmona, M.; Ruiz, L.; Cuadrado, I.M.; Velasco, L.; Janssen, D. Amaranthus leaf mottle virus: 3'-end RNA sequence proves classification as distinct virus and reveals affinities within the genus Potyvirus. *Eur. J. Plant Pathol.* **2007**, *117*, 81-87, doi 10.1007/s10658-006-9064-x.

					Lovisolo, O.; Lisa, V. Characterization of a virus isolated from *Amaranthus deflexus*, serologically related to bean yellow mosaic virus. *Agricul. Sci.* **1976**, *39*, 553-559. Lovisolo, O.; Lisa, V. Studies on amaranthus leaf mottle virus (ALMV) in the Mediterranean region. *Phytopath. Mediterranea.* **1979**, *18*, 89-93
Amazon lily mosaic virus	AB158523	Japan, Taiwan	Terami, F.; Honda, Y.; Fukumoto, F. Amazon lily mosaic virus, a new potyvirus infecting Amazon lily (*Eucharis grandiflora*). *Ann. Phytopathol. Soc. Jpn*. **1995,** 6, 1-6, doi:10.3186/jjphytopath.61.1. Hu, W.C.; Chang, Y.C. A new mosaic disease of Amazon lily in Taiwan. *Plant Pathol*. **2004,** *53*, 240	*Myzus persicae*	Terami, F.; Honda, Y.; Fukumoto, F. Amazon lily mosaic virus, a new potyvirus infecting Amazon lily (*Eucharis grandiflora*). *Ann. Phytopathol. Soc. Jpn.* **1995**, *61*, 1-6, doi:10.3186/jjphytopath.61.1.
Angelica virus Y	EF488741	USA	Robertson, N.L. Identification and characterization of a new virus in the genus Potyvirus from wild populations of *Angelica lucida* L. and *A-genuflexa* Nutt., family Apiaceae. *Arch. Virol.* **2007**, *152*, 1603-1611, doi:10.1007/s00705-007-1003-4.	Unknown	
Apium virus Y	HM363516	USA, Australia, New Zealand	Tian, T.; Liu, H.Y.; Koike, S.T. First report of apium virus Y on cilantro, celery, and parsley in California. *Plant Dis.* **2008**, *92*, 1254, doi:10.1094/PDIS-92-8-1254B. Moran, J.; Van Rijswijk, B.; Traicevski, V.; Kitajima, E.W.; Mackenzie, A.M.; Gibbs, A.J. Potyviruses, novel and known, in cultivated and wild species of the family Apiaceae in Australia. *Arch. Virol.* **2002**, *147*,1855-1867, doi:10.1007/s00705-002-0865-8. Tang, J.; Clover, G.R.; Alexander, B.J.R. First Report of apium virus Y in celery in New Zealand. *Plant Dis.* **2007**, *91*, 1682, doi:10.1094/PDIS-91-12-1682C. Baker, C.A.; Rosskopf, E.N.; Irey, M.S.; Jones, L.; Adkins, S. Bidens mottle virus and apium virus Y identified in *Ammi majus* in Florida. *Plant Dis.* **2008**, *92*, 975, doi:10.1094/PDIS-92-6-0975A. Eastwell, K.C.; Glass, J.R.; Seymour, L.M.; Druffel, K.J. First report of infection of poison hemlock and celery by apium virus Y in Washington State. *Plant Dis.* **2008**, *92*, 1710, doi:10.1094/PDIS-92-12-1710C.	*Myzus persicae*	Xu, D.; Liu, H.Y.; Koike, S.T.; Li, F.; Li, R. Biological characterization and complete genomic sequence of apium virus Y infecting celery. *Virus Res.* **2011**, *155*, 76-82, doi: 10.1016/j.virusres.2010.09.002.
Araujia mosaic virus	EF710625	New Zealand, Argentina	Elliott, M.S.; Massey, B.; Cui, X.; Hiebert, E.; Charudattan, R.; Waipara, N.; Hayes, L. Supplemental host range of araujia mosaic virus, a potential biological control agent of moth plant in New Zealand. *Australas. Plant Pathol.* **2009**, *38*, 603-607, doi:10.1071/ap09046. Charudattan, R.; Zettler F.W.; Cordo, H.A.; Christie, R.G. Partial characterization of a potyvirus infecting the milkweed vine, *Morrenia odorata*. *Phytopathology*, **1980**, *70*, 909-913, doi:10.1094/Phyto-70-909.	*Aphis nerii*, *Aphis spiraecola*, *Myzus persicae*	Charudattan, R.; Zettler, F.W.; Cordo, H.A.; Christie, R.G. Partial characterization of a potyvirus infecting the milkweed vine, *Morrenia odorata*. *Phytopathology* **1980**, *70*, 909–913 doi: 10.1094/Phyto-70-909
Arracacha mottle virus	DQ925486	Brazil, Peru	Orilio, A.F.; Lucinda, N.; Dusi A.N.; Nagata, T.; Inoue-Nagata, A.K. Complete genome sequence of arracacha mottle virus. *Arch. Virol.* **2013**, *158*, 291-295, doi:10.1007/s00705-012-1473-x. Adams, I.P.; Boonham, N.; Jones, R.A.C. Full-genome sequencing of a virus from a 33-year-old sample demonstrates that arracacha mottle virus is synonymous	*Aphis gossypii*, *Myzus persicae*	Brunt, A.A.; Crabtree, K.; Gibbs, A.J. Viruses of tropical plants. Descriptions and lists from the VIDE database.; CAB International: Wallingford, UK, 1990; ISBN : 0851986633.

			with arracacha virus Y. *Microbiol. Resour. Announce*. **2018**, *7*, e01393- 18, doi:10.1128/MRA.01393-18.		
Asparagus virus 1	KJ830760	China, Germany, USA, Japan, Italy	Li, M.J.; Ke, S.Y.; Lin, C.; Mao, Z.C.; Liu, Z.J.; Anane, F.; Zhao, M.F.; Wen, G.S. First Report of asparagus virus 1 on asparagus (*Asparagus officinalis*) in China. *Plant Dis.* **2017**, *101*, 844, doi:10.1094/PDIS-07-16-1075-PDN. Blockus, S.; Lesker, T.; Maiss, E. Complete genome sequences of two biologically distinct isolates of asparagus virus 1. *Arch. Virol.* **2015**, *160*, 569-572, doi:10.1007/s00705-014-2227-8. Mink, G.L.; Uyeda, I. Three mechanically-transmissible viruses isolated from asparagus in Washington. *Plant Dis. Rep*. **1977**, *61*, 398-401. Fujisawa, I.; Goto, T.; Tsuchizaki, T.; Iizuka, N. Host range and some properties of asparagus virus I isolated from *Asparagus officinalis* in Japan. *Ann. Phytopathol. Soc. Jap*. **1983,** 49, 299-307, doi:10.3186/jjphytopath.49.299. Bertaccini, A.; Marani, F.; Martini, L.; Ventura, A.M. Le virosi dell'asparago nell'Italia settentrionale epidemiologia e possibilità di prevenzione. *Atti. Gior. Fitopal*. **1982,** 27-33.	*Myzus persicae, Aphis craccivora*	Howell, W.E.; Mink, G.I. Properties of asparagus virus 1 isolated from Washington State asparagus. *Plant Dis.* **1985**, *69*, 1044-1046, doi: 10.1094/PD-69-1044. Fujisawa, I.; Goto, T.; Tsuchizaki, T.; Iizuka, N. Host range and some properties of asparagus virus I isolated from *Asparagus officinalis* in Japan. *Ann. Phytopathol. Soc. Jpn.* **1983**, *49*, 299-307, doi:10.3186/jjphytopath.49.299.
Banana bract mosaic virus	DQ851496	Ecuador, India, Sri Lanka, Vietnam, Western Samoa, Philippines, Costa Rica, USA, Colombia	Quito-Avila, D.F.; Ibarra, M.A.; Alvarez, R.A.; Ratti, M.F.; Espinoza, L.; Cevallos-Cevallos, J.M.; Peralta, E. First report of banana bract mosaic virus in 'cavendish' banana in Ecuador. *Plant Dis.* **2013**, *97*, 1003, doi:10.1094/PDIS-12-12-1154-PDN. Samraj, J.; Menon, M.R.; Christudas, S.P.; Satyarajan, P.K. Kokkan a new disease of banana (*Musa paradisiaca* L.). *Agric. Res. J. Keral.* **1966,** 4, 116. Rodoni, B.C.; Dale, J.; Harding, R. Characterization and expression of the coat protein-coding region of banana bract mosaic potyvirus, development of diagnostic assays and detection of the virus in banana plants from five countries in southeast Asia. *Arch. Virol.* **1999**, *144*, 1725-1737, doi:10.1007/s007050050700. Magnaye, L.; Espino, R.R.C. Banana bract mosaic, a new disease of banana. I. Symptomatology. *Philipp. Agric.* **1990**, 73, 55-59 Bateson, M.F.; Dale, J.L. Banana bract mosaic virus: characterization using potyvirus specific degenerate PCR primers. *Arch. Virol.* **1995**, *140*, 515–527, doi:10.1007/BF01718428. Wang, I.C.; Sether, D.M.; Melzer, M.J.; Borth, W.B.; Hu, J.S. First report of banana bract mosaic virus in flowering ginger in Hawaii. *Plant Dis.* **2010**, *94*, 921, doi:10.1094/PDIS-94-7-0921A.	*Aphis craccivora, Pentalonia nigronervosa, Aphis gossypii, Rhopalosiphum maidis*	Selvarajan, R.; Balasubramanian, V.; Sathiamoorthy, S. Vector transmission of banana bract mosaic and banana streak viruses in India Abstracts of XVI annual convention and international symposium on management of vector-borne viruses, ICRISAT 2006, 110. Magnaye, L.V.; Espino, R.R.C. Note: Banana bract mosaic, a new disease of banana I. symptomatology. *The Philipp. Agric.* **1990**, *73*, 55-59 Munez, A.R. Symptomatology, transmission and purification of banana bract mosaic virus (BBMV) in 'giant cavendish' banana Faculty of graduate school, University of Philippines, Los Baños **1992**, pp.1-57
Barbacena virus Y	KU685505	Brazil	Zheng, Y.; Gao, S.; Padmanabhan, C.; Li, R.; Galvez, M.; Gutierrez, D.; Fuentes, S.; Ling, K.S.; Kreuze, J.; Fei, Z. Virus detect: an automated pipeline for efficient virus discovery using deep sequencing of small RNAs. *Virology* **2017**, *500*, 130–138, doi:10.1016/j.virol.2016.10.017.	Unknown	
Basella rugose mosaic virus	DQ821938	China, Taiwan, Nigeria	Wang, J.G.; Peng, J.J.; Chen, H.R.; Chen, S.Y. First report of basella rugose mosaic virus infecting four o'clock (*Mirabilis jalapa*) in China. *Plant Dis*. **2012**, *96*, 294, doi:10.1094/PDIS-07-11-0586. Huang, C.H.; Chang, Y.C. Basella rugose mosaic virus, a new potyvirus infecting *Basella rubra*. *Plant Pathol.* **2006**, *55*, 819, doi:10.1111/j.1365-3059.2006.01477.x.	Unknown	

			Adediji, A.O.; Atiri, G.I. First report of basella rugose mosaic virus infecting *Basella alba* in Nigeria. *J. Plant Pathol.* **2020**, doi:10.1007/s42161-020-00523-4.		
Basella rugose mosaic virus	DQ851494	Vietnam	Ha, C.; Coombs, S.; Revill, P.A.; Harding, R.M.; Vu, M.; Dale, J.L. Design and application of two novel degenerate primer pairs for the detection and complete genomic characterization of potyviruses. *Arch. Virol.* **2008**, *153*, 25-36, doi:10.1007/s00705-007-1053-7.	Unknown	
Bean common mosaic necrosis virus	U19287	Korea, Uganda, Nepal, Turkey, Argentina, Australia, Belize, Brazil, Burundi, Canada, Chile, China, Colombia, Congo, Costa Rica, Cuba, Dominican Republic, Egypt, El Salvador:, Ethiopia, Guatemala, Haiti, Honduras, India, Indonesia, Iran, Japan, Jamaica, Kenya, Lebanon, Lesotho, Malaysia, Malawi, Mexica, Myanmar, Netherlands, New Zealand, Nicaragua, Nigeria, Panama, Paraguay, Peru, Philippines, Poland, Puerto Rico, Russia, Rwanda, Serbia, South Korea, Spain, Swaziland: Taiwan, Tanzania, Thailand, Turkey, United Kingdom, United States, Uganda, Vietnam, Zambia, Zimbabwe	Jang, Y.W.; Jo, Y.; Cho, W.K.; Choi, H.; Yoon, Y.N.; Lim, S.M.; Lee, Y.H.; Bae, J.Y.; Lee, B.C. First report of bean common mosaic necrosis virus infecting soybean in Korea. *Plant Dis.* **2018**, *102*, 2051, doi:10.1094/PDIS-09-17-1474-PDN.<br>Sengooba, T.N.; Spence, N.J.; Walkey, D.G.A.; Allen, D.J.; Femi Lana, A. The occurrence of bean common mosaic necrosis virus in wild and forage legumes in Uganda. *Plant Pathol.* **1997**, *46*, 95-103, doi: 10.1046/j.1365-3059.1997.d01-12.x<br>Pudashini, B.J.; Shahid, M.S.; Natsuaki, K.T. First report of bean common mosaic necrosis virus (BCMNV) infecting sweet bean in Nepal. *Plant Dis.* **2013**, 97, 290-290, doi:10.1094/PDIS-08-12-0741-PDN.<br>Deligoz, I.; Arli-Sokmen, M. Differentiation of bean common mosaic virus (BCMV) and bean common mosaic necrosis virus (BCMNV) strains infecting common bean in Samsun Province. *J. Turk. Phytopathol.*, **2008**, *37*, 1–14, doi:10.1016/bs.aivir.2015.04.002.<br>Worrall, E.A.; Wamonje, F.O.; Mukeshimana, G.; Harvey, J.J.; Carr, J.P.; Mitter, N. Bean common mosaic virus and bean common mosaic necrosis virus: relationships, biology, and prospects for control. *Adv. Virus. Res.* **2015**, *93*, 1-46, doi:10.1016/bs.aivir.2015.04.002.<br>Plantwise Knowledge Bank on bean common mosaic necrosis virus. Available online: https://www.plantwise.org/KnowledgeBank/datasheet/40617 (Accessed on 28 March 2020).	*Aphis fabae, Acyrthosiphon pisum, Aphis craccivora, Myzus persicae, Macrosiphum solanifolii, Macrosiphum pisi, Macrosiphum ambrosiae, Aphis rumicis, Aphis gossypii, Aphis medicaginis, Hyalopterus atriplicis, Rhopalosiphum pseudobrassicae*	Silbernagel, M.J.; Mink, G.I.; Zhao, R.L.; Zheng, G.Y. Phenotypic recombination between bean common mosaic and bean common mosaic necrosis potyviruses in vivo. *Arch. Virol.* **2001**, *146*, 1007-1020, doi: 10.1007/s007050170132.<br>Zaumeyer, W.; Meiners, J. Disease resistance in beans. *Annu. Rev. Phytopathol.* **1975**, *13*, 313-334, doi: 10.1146/annurev.py.13.090175.001525.
Bean common mosaic virus	AJ312437	Australia, Belize, Brazil, Burundi, Canada, Chile, China, Colombia, Congo, Costa Rica, Cuba,	Worrall, E.A.; Wamonje, F.O.; Mukeshimana, G.; Harvey, J.J.; Carr, J.P.; Mitter, N. Bean common mosaic virus and bean common mosaic necrosis virus: relationships, biology, and prospects for control. *Adv. Virus. Res.* **2015**, *93*:1- 46, doi: 10.1016/bs.aivir.2015.04.002.	*Macrosiphum solanifolii, Macrosiphum pisi, Macrosiphum ambrosiae, Myzus persicae,*	Zaumeyer, W.; Meiners, J. Disease resistance in beans. *Annu. Rev. Phytopathol.* **1975**, *13*, 313-334, doi: 10.1146/annurev.py.13.090175.001525.<br>Zettler, F.W.; Wilkinson, R.E. Effect of probing behavior and starvation of *Myzus persicae* on

		Dominican Republic, Egypt, El Salvador, Ethiopia, Guatemala, Haiti, Honduras, India, Indonesia, Iran, Japan, Jamaica, Kenya, Lebanon, Lesotho, Malaysia, Malawi, Mexica, Myanmar, Netherlands, New Zealand, Nicaragua, Nigeria, Panama, Paraguay, Peru, Philippines, Poland, Puerto Rico, Russia, Rwanda, Serbia, South Korea, Spain, Swaziland, Taiwan, Tanzania, Thailand, Turkey, United Kingdom, United States, Uganda, Vietnam, Zambia, Zimbabwe		*Aphis rumicis, Aphis gossypii, Aphis medicaginis, Hyalopterus atriplicis, Rhopalosiphum pseudobrassicae, Metopholophium dirhodum, Rhopalosiphum padi, Schizaphis graminum, Sitobion avenae, Myzus persicae, Aphis pisum, Aphis craccivora, Aphis fabae*	transmission of bean common mosaic virus. *Phytopathology*, **1966**, *56*,1079-1082 Halbert, S.E.; Mink, G.I.; Silbernagel, M.J.; Mowry, T.M. Transmission of bean common mosaic virus by cereal aphids (Homoptera: Aphididae). *Plant Dis.* **1994**, *78*, 983-985, doi: 10.1094/PD-78-0983. Silbernagel, M.J.; Mink, G.I.; Zhao, R.L.; Zheng, G.Y. Phenotypic recombination between bean common mosaic and bean common mosaic necrosis potyviruses in vivo. *Arch. Virol.* **2001**, *146*, 1007-1020, doi: 10.1007/s007050170132.
Bean yellow mosaic virus	D83749	Egypt, Ethiopia Kenya, Libya, Morocco, South Africa, Sudan, Tanzania, Tunisia, Zambia, Zimbabwe, China, Georgia, India, Indonesia Iran, Iraq, Israel, Japan, Jordan, Kazakhstan, Lebanon, Pakistan, South Korea, Syria, Taiwan, Turkey, Uzbekistan, Yemen, Belarus, Belgium Bulgaria, Croatia, Czechia, Czechoslovakia, Federal Republic of, Yugoslavia, Denmark,	Cabi datasheet on bean yellow mosaic virus. Available online: https://www.cabi.org/isc/datasheet/9433 (Accessed on 28 March 2020).	*Acyrthosiphon kondoi, Acyrthosiphon pisum, Aphis craccae, Aphis craccivora, Aphis fabae, Aphis glycines, Aphis gossypii, Aulacorthum circumflexum, Aulacorthum solani, Brachycaudus cardui, Chaetosiphon fragaefolii, Dysaphis crataegi, Hayhurstia atriplicis, Hyperomyzus lactucae, Lipaphis erysimi, Macrosiphum euphorbiae,*	Kennedy, J.S.; Day, M.F.; Eastop, V.F. A conspectus of aphids as vectors of plant viruses. Wallingford, UK: Cab International **1962.** Malone, M.T. Resistance of green beans to aphid-borne viruses in New Zealand. *In Proceedings of the Thirty-First New Zealand Weed and Pest Control Conference. Devon Motor Lodge, New Plymouth, 8th to 10th August 1978*; Hartley, M.J, ed. **1978**. New Zealand Weed and Pest Control Society Inc.: Palmerston North, New Zealand, 1978; pp. 85-88. Yahia, A.A.; Ouada, M.A.; Illoul, H.; Tair, M.I. First occurrence of bean yellow mosaic potyvirus on chickpea in Algeria. *Bull. OEPP* **1997**, *27*, 261-263, doi: 10.1111/j.1365-2338.1997.tb00645.x. Blackman, R.L.; Eastop, V.F. *Aphids on the world's crops: an identification and information*

		Finland, France, Germany, Greece, Hungary, Italy, Lithuania, Netherlands, Poland Portugal, Romania Russia, Spain, Sweden, Ukraine, United Kingdom, Canada. Dominican Republic, Jamaica, Mexico, Montserrat, United States, Australia, New Zealand, Argentina, Chile, Peru		*Macrosiphum rosae, Myzus cerasi, Myzus certus, Myzus persicae, Nearctaphis bakeri, Sitobion avenae, Megoura viciae*	*guide*, 2nd ed.; Wiley: London, UK, 2000; ISBN: 978-0-471-85191-2. Gaudchau, M. Studies on the transmission of bean yellow mosaic virus (BYMV) by aphids. *Zeitschrift fur Pflanzenkrankheiten und Pflanzenschutz* **1978**, *85*, 347-357.
Beet mosaic virus	AY206394	China, Germany, USA, New Zealand, Iran, Netherlands, UK, Kenya, Slovakia, Egypt	Xiang, H.; Han, Y.H.; Han, C.; Li, D.; Yu. J. Molecular characterization of two Chinese isolates of beet mosaic virus. *Virus Gen.* **2007**, *35*, 795-799, doi:10.1007/s11262-007-0132-x. Hasan, H. Generation of an infectious beet mosaic virus (BtMV) full- length clone based on the complete nucleotide sequence of a German isolate. Doctoral Thesis. University of Hannover, Hannover, Germany, 2004. Jones, L.K. 1931. *Mosaic disease of beets*. Washington Agricultural Experiment Station Bulletin, Washington, USA. Chamberlain, E.E. Plant virus diseases in New Zealand. *N. Z. Dep. Sci. Ind. Res. Bull.* **1954,** *108*, 255. Mohammadi, M.; Gibbs, A.J.; Hosseini, A.; Hosseini, S. An Iranian genomic sequence of beet mosaic virus provides insights into diversity and evolution of the world population. *Virus Gen.* 2018, 54, 272-279, doi:10.1007/s11262-018-1533-8. Spence, N.J.; Phiri, N.A.; Hughes, S.L.; Mwaniki, A.; Simons, S.; Oduor, G.; Chcha, D.; Kuria, A.; Ndirangu, S.; Kibata, G.N.; Marries, G.C. Economic impact of turnip mosaic virus, cauliflower mosaic virus and beet mosaic virus in three Kenyan vegetables. *Plant Pathol.* **2007**, *56*, 317-323, doi: 10.1111/j.1365-3059.2006.01498.x. Glasa, M.; Kudela, O.; Subr, Z. Molecular analysis of the 3′ terminal region of the genome of beet mosaic virus and its relation with other potyviruses. *Arch. Virol.* **2003**, *148*, 1863-1871, doi: 10.1007/s00705-003-0126-5. Haggag, W.H.; Mahmoud, Y.S.; Farag, E.M. Signaling necessities and function of polyamines/ jasmonate -dependent induced resistance in sugar beet against beet mosaicvirus (BtMV) infection. *N. Y. Sci. J.* **2003,** *3*, 95-103.	*Myzus persicae, Rhopalosiphum padi, Acyrthosiphon kondoi, Diuraphis noxia, Aphis fabae, Acyrthosiphon pisum, Aphis fabae, Macrosiphum euphorbiae, Myzus persicae, Ropalosiphum padi, Cavariella aegopodii, Aphis pomi, Acyrthosiphon primulae, Aphis armata, Aphis armoraciae, Aphis evonymi Aphis helianthin, Aphis nasturtii, Aphis sambuci, Brachycaudus prunicola, Brachycaudus tragopogonis, Cavariella archangelicae,*	Dusi, A.N.; Peters, D. Beet mosaic virus: its vector and host relationships. *J. Phytopathol.* **1999**, *147*, 293-298, doi: 10.1046/j.1439-0434.1999.147005293.x Summers, C.G.; Newton, A.S.; Kirk, M.; Temple, S.R. Transmission of beet yellows and beet mosaic viruses by non-colonizing aphid vectors. *J. Econ. Entomol.* **1990**, *83*, 2448-2451, doi: 10.1093/jee/83.6.2448. Watson, M.A.; Hull, R.; Blencowe, J.W.; Hamlyn, B.M.G. The spread of beet yellows and beet mosaic viruses in the sugar-beet root crop. I. Field observations on the virus diseases of sugar beet and their vectors *Myzus persicae* Sulz. and *Aphis fabae* Koch. *Ann. Appl. Biol.* **1951**, *38*, 743-64, doi: 10.1111/j.1744-7348.1951.tb07847.x. Edwardson, J.R.; Christie, R.G. *CRC Handbook of Viruses Infecting Legumes*, 1st ed.; CRC Press: Baco Ranton, Florida, USA, 1991; ISBN: 978-08-493-4729-0. Carter, W. *Insects in relation to plant disease, Interscience*, Wiley: New York, USA, 1962; pp. 705. Kennedy, J.S.; Day, M.F.; Eastop, V.F. A *conspectus of aphids as vectors of plant viruses,* Commonweath institute of entomology: London, UK, 1962.

				Cerosipha apigraveolens, Crytomyzus ribis, Dactynotus cichoricola, Dysaulacorthum pseudosolanii, Hayhurstia atriplicis, Lipaphis pseudobrassicae, Macrosiphum solanifolii, Megoura viciae, Sitobion avenae, Rhopalosiphum padi, Metopolophium dirhodum, Macrosiphum euphorbiae, Dysaphis apiifolia, Hyadaphis foeniculi, Hyadaphis erysimi, Hyperomyzus lactucae, Idiopterus nephrolepidia, Metopolophium primulae, Macrosiphoniella oblonga, Myzotoxoptera tulipella, Myzus ajugae, Myzus ascalonicus, Rhopalosiphonius tulipaellus,	
Bidens mosaic virus	KF649336	Brazil	Orílio, A.F.; Dusi, A.N.; Madeira, N.R.; Inoue-Nagata, A.K. First Report of Bidens mosaic virus in Arracacha (*Arracacia xanthorriza*) From Brazil. *Plant Dis.* **2016**, *101*, 262-262, doi: 10.1094/PDIS-05-16-0616-PDN	*Aphis coreopsidis*	Descriptions and Lists from the VIDE Database: Bidens mosaic potyvirus. Available online: http://biomirror.im.ac.cn/mirrors/pvo/vide/descr097.htm (Accessed on 8 July 2020).
Bidens mottle virus	AF538686	Taiwan, USA	Chen, Y.K.; Lee, J.Y. First Report of Bidens mottle virus causing mosaic and leaf deformation in garland chrysanthemum and lettuce in Taiwan. *Plant. Dis.* **2012**, *96*, 464, doi: 10.1094/PDIS-05-16-0616-PDN.	*Hyadaphis pseudobrassicae*	Sastry, K.S..; Mandal, B..; Hammond, J..; Scott, S.W.; Briddon, R.W. *Bidens* spp. (Beggarticks). In: *Encyclopedia of Plant*

			Baker, C.A.; Raid, R.N.; Scully, B.T. Natural infection of *Vicia faba* by Bidens mottle virus in Florida. *Plant Dis.* **2001**, *85*, 1290, doi: 10.1094/PDIS.2001.85.12.1290C.		*Viruses and Viroids*. Springer: New Delhi, India, 2019, ISBN: 978-81-322-3912-3.
Blue squill virus A	JQ807999	Australia	Wylie, S.J.; Li, H.; Dixon, K.W.; Richards, H.; Jones, M.G.K. Exotic and indigenous viruses infect wild populations and captive collections of temperate terrestrial orchids (*Diuris species*) in Australia. *Virus Res.* **2013**, 171, 22-32, doi: 10.1016/j.virusres.2012.10.003.	Unknown	McLeab, D.L.; Kinsey, M.G. Transmission studies of a highly virulent variant of lettuce mosaic virus. *Plant. Dis. Rep.* **1963**, *47*, 474-476.
Brugmansia mosaic virus	JX867236	USA, South Korea	Damsteegt, V.D.; Stone, A.L.; Smith, O.P.; McDaniel, L.; Sherman, D.J.; Dardick, C.; Hamond, J.; Jordan, R.; Schneider, W.L. A previously undescribed potyvirus isolated and characterized from arborescent Brugmansia. *Arch. Virol*. 2013, 158, 1235-1244, doi: 10.1007/s00705-012-1600-8. Park, C.Y.; Kim, B.S.; Nam, M.; Lee, M.A.; Baek, D.S.; Bae, Y.S.; Park, E.H.; Kim, J.S.; Choi, J.Y.; Lim, S.M.; Moon, J.S.; Lee, S.H. Characterization of brugmansia mosaic virus isolated from *Brugmansia spp*. in Korea. *Res. Plant Dis*. **2014**, *20*, 307–313, doi: 10.5423/RPD.2010.16.	*Myzus persicae*	Damsteegt, V.D.; Stone, A.L.; Smith, O.P.; McDaniel, L.; Sherman, D.J. et al. A previously undescribed potyvirus isolated and characterized from arborescent Brugmansia. *Arch. Virol.* **2013,** *158*, 1235-44.
Brugmansia suaveolens mottle virus	AB551370	Brazil	Lucinda, N.; Nagata, T.; Inoue-Nagata, A.K.; Salaroli, R.B.; Kitajima, E.W. Brugmansia suaveolens mottle virus, a novel potyvirus causing leaf mottling of *Brugmansia suaveolens* in Brazil. *Arch. Virol*. **2008**, *153*, 1971-1976, doi: 10.1007/s00705-008-0211-x.	*Myzus persicae*	Lucinda, N.; Nagata, T.; Inoue-Nagata, A.K.; Salaroli, R.B.; Kitajima, E.W. Brugmansia suaveolens mottle virus, a novel potyvirus causing leaf mottling of *Brugmansia suaveolens* in Brazil. *Arch. Virol.* **2008**, *153*, 1971-1976, doi: 10.1007/s00705-008-0211-x.
Butterfly flower mosaic virus	AM774001	China	Chen, J.; Shi, Y.H.; Li, M.Y.; Adams, M.J.; Chen, J.P. A new potyvirus from butterfly flower (*Iris japonica Thunb.*) in Zhejiang, China. *Arch. Virol.* **2008**, *153*, 567-569, doi:10.1007/s00705-007-0014-5.	Unknown	
Calanthe mild mosaic virus	AB011404	Japan, India	Gara, I.; Kondo, H.; Maeda, T.; Inouye, N.; Tamada, T. Calanthe mild mosaic virus, a new potyvirus causing mild mosaic disease of the Calanthe orchid in Japan. *J. Phytopath.* **1998**, *146*, 357-363, doi: 10.1111/j.1439-0434.1998.tb04704.x. Pant, R.P.; Kapoor, R.; Kumar, S.; Srivastava, N.; Kumar, M.; Baranwal, V.K. First report of mild mosaic in ground orchid, *Phaius tankervilleae*, in India associated with infection of calanthe mild mosaic virus. *Plant Dis.* **2017**, *101*, 1960-1960, doi: 10.1094/PDIS-06-17-0792-PDN	*Myzus persicae*	Gara, I.; Kondo, H.; Maeda, T.; Inouye, N.; Tamada, T. Calanthe mild mosaic virus, a new potyvirus causing mild mosaic disease of the Calanthe orchid in Japan. *J. Phytopath.* **1998**, *146*, 357-363, doi: 10.1111/j.1439-0434.1998.tb04704.x.
Calla lily latent virus		Taiwan	Chen, C.C.; Chang, C.A.; Tsai, H.T.; Hsu, H.T. Identification of a potyvirus causing latent infection in calla lilies. *Plant Dis.* **2004**, *88*, 1046, doi: 10.1094/PDIS.2004.88.9.1046A.	Unknown	
Callistephus mottle virus	KX013584	South Korea	Seo, E.Y.; Lim, S.; Hammond, J.; Moon, J.S.; Lim, H.S. Complete genome sequence of a novel potyvirus, callistephus mottle virus, identified in *Callistephus chinensis*. *Arch. Virol.* **2016**, *161*, 3281-3283, doi: 10.1007/s00705-016-3001-x.	Unknown	
Canna yellow streak virus	GQ421689	UK, Belgium, Netherlands, France, Israel, USA, Brazil	Monger, W.A.; Harju, V.; Skelton, A.; Seal, S.E.; Mumford, R.A. Canna yellow streak virus: A new potyvirus associated with severe streaking symptoms in canna. *Arch. Virol.* **2007**, *152*, 1527-1530, doi: 10.1007/s00705-007-0977-2. Verchot, J.; RajaKaruna, P. *Plant Viruses Infecting Cannas*. Oklahoma Coop. Ext. Srv. EPP- 7327	Unknown	

			Alexandre, M.A.V.; Duarte, L.M.L.; Chaves, A.L.R.; Ramos, A.F.; Harakava, R.; Kitajima, E.W. *Canna paniculata* as natural host of canna yellow streak virus in Brazil. *Aust. Plant. Dis. Notes* **2017**, *12*, 38, doi: 10.1007/s13314-017-0264-3.		
Carnation vein mottle virus	AB017630	China, South Korea, India	Li, M.R.; Zhu, J.; Gao, J.; Li, R.H.; Li, F. First report of carnation vein mottle virus infecting *Dianthus amurensis* in China. *Plant Dis*. **2014**, *98*, 1747, doi: 10.1094/PDIS-05-14-0453-PDN.<br>Chung, B.N.; Kim, B.D.; Choi, G.S.; Kim, J.S. First Report on carnation vein mottle virus in *Dianthus barbatus* in Korea. Plant Pathol. J. **2003**, *20*, 3, doi: 10.5423/PPJ.2004.20.3.224.<br>Raikhy, G.; Hallan, V.; Kulshrestha, S.; Sharma, M.L.; Verma, N.; Ram, R.; Zaidi, A.A. Detection of carnation ringspot and carnation vein mottle viruses in carnation cultivars in India. ISHS Acta Horticulturae 722: XI International Symposium on Virus Diseases of Ornamental Plants, Taichung, Taiwan, 31 October 2006, doi: 10.17660/ActaHortic.2006.722.31.	Unknown	
Carrot thin leaf virus	JX156434	Slovenia, USA	Mehle, N.; Kutnjak, D.; Tušek Žnidarič, M.; Ravnikar, M. First Report of apium virus Y and carrot thin leaf virus in parsley in Slovenia. *Plant Dis.* **2019**, *103*, 592, doi: 10.1094/PDIS-04-18-0690-PDN.<br>Falk, B.W.; Davis, R.M.; Piechocki, M. Identification of carrot thin leaf virus in California carrots. *Plant Dis.* **1991**, *75*, 319, doi: 10.1094/PD-75-0319C.	*Myzus persicae, Cavariella aegopodii*	Howell, W.E.; Mink, G.I. Host range, purification, and properties of a flexuous rod-shaped virus isolated from carrot. *Phytopathology* **1976**, *66*, 949-953.
Carrot virus Y	AF203537	Australia, USA	Moran, J.; Gibbs, A.; Van Rijswijk, B.; Mackenzie, A.; Gibbs, M.; Traicevski, V. Potyviruses in the cultivated and wild Apiaceae in Australia and the implications for disease control. In Proceedings of the 12th Biennial Conference of the Australasian Plant Pathological Society, Canberra, Australia, 27-30 September 1999; pp. 132.<br>Howell, W.E.; Mink, G.I. The role of weed hosts, volunteer carrots and overlapping growing seasons in the epidemiology of carrot thin leaf and carrot motley dwarf viruses in central Washington. *Plant Dis.* **1977**, *61*, 217-222.	*Myzus persicae, Aphis spiraecola, Dysaphis foeniculus, Dysaphis apiifolia, Hyadaphis foeniculi, Hyadaphis coriandri, Lipahis erysimi, Acyrthosiphon kondoi, Hysteroneura setariae, Rhopalosiphum maidis, Rhopalosiphum padi*	Jones, R.A.C.; Smith, L.J.; Gajda, B.E.; Smith, T.N.;Latham, L.J. Carrot virus Y: symptoms, losses, incidence, epidemiology and control in Australia. *Virus Res.* **2004**, *100*, 89-99, doi: 10.1016/j.virusres.2003.12.017.
Catharanthus mosaic virus	KP343681	Australia, Brazil, USA, Saudi Arabia	Koh, S.H.; Li, H.; Admiraal, R.; Jones, M.G.K.; Wylie, S.J. Catharanthus mosaic virus: a potyvirus from a gymnosperm, *Welwitschia mirabilis*. *Virus Res.* **2015**, *203*, 41-46, doi: 10.1016/j.virusres.2015.03.007.<br>Maciel, S.C.; Silva, R.F.d.; Reis, M.S.; Jadão, A.S.; Rosa, D.D.; Giampan, J.S.; Kitajima, E.W.; Rezende, J.A.M.; Camargo, L.E. Characterization of a new potyvirus causing mosaic and flower variegation in *Catharanthus roseus* in Brazil. *Sci. Agric*. **2011**, 68, 687-690, doi: 10.1590/S0103-90162011000600013.<br>Mollov, D.; Guaragna, M.A.; Lockhart, B.; Rezende, J.A.M.; Jordan, R. First report of catharanthus mosaic virus in Mandevilla in the United States. *Plant Dis.* **2014,** 99, 165, doi: 10.1094/PDIS-09-14-0913-PDN.<br>Al-Zahrany, H.M.; Elbeshehy, E.K.F.; Aldhebiani, A.Y.; Almaghrabi, O.; Al-Jaddawi, A.A.; Alzahrani, A.H. Effect of catharanthus mosaic virus (CatMV) on	*Aphis gossypii, Myzus nicotianae*	Maciel, S.C.; Silva, R.F.d.; Reis, M.S.; Jadão, A.S.; Rosa, D.D.; Giampan, J.S.; Kitajima, E.W.; Rezende, J.A.M.; Camargo, L.E. Characterization of a new potyvirus causing mosaic and flower variegation in *Catharanthus roseus* in Brazil. *Sci. Agric.* **2011**, *68*, 687-690, doi: 10.1590/S0103-90162011000600013.

			antineoplastic alkaloids from periwinkle (*Catharanthus roseus* L.) cultured in Mecca region and resistance induction by plant growth-promoting rhizobacteria. *Biotechnol. Biotechnol. Equip.* **2019,** 33, 392-1401, doi: 10.1080/13102818.2019.1669490.		
Celery mosaic virus	HQ676607	Egypt, Venezuela, Iran, Australia, UK, USA	Amal, A.A.; Zein Salwa, N.; Khatab Eman, A.H. Characterization of celery mosaic virus isolated from some apiaceae plants. *Int. J. Virol.* **2012**, *8*, 214-223, doi: 10.3923/ijv.2012.214.223. Fernández, T.; Carballo, O.; Zambrano, K.; Romano, M.; Marys, E. First Report of celery mosaic virus infecting celery in Venezuela. *Plant Dis.* **2007**, *90*, 1111, doi: 10.1094/PD-90-1111A. Khoshkhatti, N.; Habibi, M.K.; Mosahebi, G. Characterization of celery mosaic virus from celery in Tehran province Iranian. *J. Vir.* **2011,** *5*, 10-14, doi: 10.21859/isv.5.1.10. Walkey, D.G.A.; Tomlinson, J.A.; Frowd, J.A. Occurrence of western celery mosaic virus in umbelliferus crops in Britain. *Plant Dis. Rpt*. **1970**, 53, 370–371. Brunt, A.A.; Crabtree, K.; Dallwitz, M.J.; Gibss, A.J.; Watson, L.; Zurcher, E.J. Plant viruses online: Descriptions and list from the VIDE database. Version: 16 Jan. 1997. Latham, L.J.; Jones, R.A.C. Incidence of celery mosaic virus in celery crops in south-west Australia and its management using a celery-free period. *Austral. Plant Pathol.,* **2003**, 32, 527–531, doi: 10.1071/AP03058.	*Myzus persicae, Semiaphis dauci*	Amal, A.A.; Zein Salwa, N.; Khatab Eman, A.H. Characterization of celery mosaic virus isolated from some apiaceae plants. *Int. J. Virol.* **2012**, *8*, 214-223, doi: 10.3923/ijv.2012.214.223. Blackman, R.L.; eastoP, V.F. *Aphids on the world's crops: an identification and information guide*, 1st.; John Wiley & Sons: Chichester, UK, 2000; pp. 466.
Ceratobium mosaic virus	AF022442	Australia	Mackenzie, A.M.; Nolan, M.; Wei, K.J.; Clements, M.A.; Gowanlock, D.; Wallace, B.J.; Gibbs. A.J. Ceratobium mosaic potyvirus: another virus from orchids. *Arch. Virol*. **1998**, 143, 903-914, doi:10.1007/s007050050341.	*Sitobion luteum*	Mackenzie, A.M.; Nolan, M.; Wei, K.J.; Clements, M.A.; Gowanlock, D.; Wallace, B.J.; Gibbs, A.J. Ceratobium mosaic potyvirus: another virus from orchids. *Arch. Virol*. **1998**, 143, 903-914
Chilli ringspot virus	JN008909	China, Pakistan, Laos	Wang, J.H.; Zhang, S.Y.; Gong, D.; Wu, Y.P.; Zhang, Y.L.; Yu, N.T.; Liu, Z.X.; Xiong, Z. First report of chilli ringspot virus on chili pepper in China. *Plant Dis.* **2011,** 96, 462, doi: 10.1094/PDIS-11-11-0922. Ahmad, A.; Ashfaq, M.; Ahsan, M. 2017. First report of chilli ringspot virus on chilli pepper in Pakistan. *J. Plant Pathol.* **2017,** 99, 799-818, doi: 10.4454/jpp.v99i3.3977. Chittarath, K.; Rungsawang, W.; Pongsapich, P.; Kong, G.A.; Thomas, J.E.; Geering, A.D.W. First records of the potyviruses chilli ringspot virus and shallot yellow stripe virus from Laos. *Austral. Plant Dis. Notes,* **2017**, 12, 53, doi: 10.1007/s13314-017-0278-x.	Unknown	
Chilli veinal mottle virus	AJ237843	China, India, Italy, Australia, Japan, Korea Democratic People's Republic, Korea Republic, Malaysia, Pakistan, Philippines,	Zhu, F.; Che, Y.P.; Qian, K.; Zhou, Y.K.; Xu, Y.J.;. Zhao, Y.Q.; Ji, Z.L. First report of chilli veinal mottle virus infecting *Lycopersicon esculentum* in Jiangsu province in China. *Plant Dis.* **2017**, *102*, 462-462, doi: 10.1094/PDIS-07-17-1100-PDN. Banerjee, A.; Dutta, R.; Roy, S.; Ngachan, S.V. First report of chilli veinal mottle virus in Naga chilli (*Capsicum chinense*) in Meghalaya, India. *VirusDisease* **2014**, *25*, 142–143, 10.1007/s13337-013-0167-7.	*Myzus persicae, Aphis gossypii, Aphis craccivora, Aphis spiraecola, Rhopalosiphum maidis,*	Cerkauskas, R. AVRDC-The world vegetable center fact sheet, Pepper Diseases: Chilli Veinal Mottle Virus. Available online: http://203.64.245.61/web_crops/pepper/chivmv.pdf (accessed on 30 March 2020).

		Sri Lanka, Taiwan, Thailand, Vietnam, Tanzania, Papua New Guinea, Indonesia	Tiberini, A.; Manglli, A.; Ahmad, A.; Cassia, U.; Tomassoli, L. First report and molecular identification of chilli veinal mottle virus in Italy. *J. Plant Pathol.* **2017**, *99*, 523, doi: 10.4454/jpp.v99i2.3891.<br>CABI, 2015. Chilli veinal mottle virus. In: Distribution Maps of Plant Diseases, map 1173. 1st Ed. CABI, Wallingford, UK	*Toxoptera citrida, Hysteroneura setariae*	
Chinese artichoke mosaic virus	AB099711	Japan	Fuji, S.; Yamamoto, H.; Furuya, H.; Naito, H. Characterization of a new potyvirus isolated from Chinese artichoke in Japan. *Arch. Virol.* **2003**, *148*, 2249-55, doi: 10.1007/s00705-003-0169-7.	Unknown	
Clitoria virus Y	AF228515	Australia	Gibbs, A.J.; Mackenzie, A.M.; Wei, K.J.; Gibbs, M.J. The potyviruses of Australia. *Arch. Virol.* **2008**, *153*, 1411-20, doi: 10.1007/s00705-008-0134-6.	Unknown	
Clover yellow vein virus	AB011819	USA, Japan, UK, Sweden, New Zealand, Australia, Canada, Korea	Larsen, R.C.; Miklas, P.N.; Eastwell, K.C.; Grau, C.R. A strain of clover yellow vein virus that causes severe pod necrosis disease in snap bean. *Plant Dis.* **2008**, *92*, 1026–1032, doi: doi: 10.1094/PDIS-92-7-1026.<br>Sasaya, T.; Shimizu, T.; Nozu, Y.; Nishiguchi, M.; Inouye, N.; Koganezawa, H. Biological, serological, and molecular variabilities of clover yellow vein virus. *Phytopathology* **1997**, *87*, 1014-9, doi: 10.1094/PHYTO.1997.87.10.1014.<br>Hollings, M.; Nariani, T. K. Some properties of clover yellow vein, a virus from *Trifolium repens* L. *Ann. Appl. Biol.* **1965**, *56*, 99-109, doi: 10.1111/j.1744-7348.1965.tb01219.x.<br>Bos, L.; Lindsten, K.; Maat, D.Z. Similarity of clover yellow vein virus and pea necrosis virus. *Neth. J. Plant Pathol.* **1977**, *83*, 97-108, doi: 10.1007/BF01981555.<br>Bryan, G.T.; Gardner, R.C.; Forster, R.L.S. Nucleotide sequence of the coat protein gene of a strain of clover yellow vein virus from New Zealand: Conservation of stemloop structure in the 3′ region of potyviruses. *Arch. Virol.* **1992**, *124*, 133-146, doi: 10.1007/bf01314631.<br>Hewish, D.R.; Xiao, X.W.; Mishra, A.; Gough, K.H.; Shukla, D.D. Characterization and epitope analysis of monoclonal antibodies to virions of clover yellow vein and johnsongrass mosaic potyviruses. *Arch. Virol.* **1993**, *133*, 127-141, doi: 10.1007/BF01309749.<br>Pratt, M.J. Clover viruses in eastern Canada in 1967. *Can. Pl. Dis. Surv.* **1968**, *48*, 87–90.<br>Shin, J.C.; Kim, M.K.; Kwak, H.R.; Choi, H. S.; Kim, J. S.; Park, C. Y.; Cha, B. J. First Report of clover yellow vein virus on *Glycine max* in Korea. *Plant Dis.* **2014**, *98*, 1283, doi: 10.1094/PDIS-11-13-1115-PDN.	*Aphis glycines, Myzus persicae, Aphis pisum, Aulacorthum solani, Macrosiphum euphorbiae*	Larsen, R.C.; Miklas, P.N.; Eastwell, K.C.; Grau, C.R. A strain of clover yellow vein virus that causes severe pod necrosis disease in snap bean. *Plant Dis.* **2008**, *92*, 1026–1032, doi: doi: 10.1094/PDIS-92-7-1026.<br>Hollings, M.; Nariani, T. K. Some properties of clover yellow vein, a virus from *Trifolium repens* L. *Ann. Appl. Biol.* **1965**, *56*, 99-109, doi: 10.1111/j.1744-7348.1965.tb01219.x.
Cocksfoot streak virus	AF499738	Germany, UK, Lithuania, Holland, Denmark, France, USA, Russia	Schumann, K. The occurrence of the cocksfoot streak virus. *Zentralbl Bakteriol Parasitenkd Infektionskr Hyg.* **1969**, *123*, 315-318. (Article in German)<br>Smith, K.M. A virus disease of cookfoot. *Plant path.* **1952**, *I*, 8.<br>Urbanavièienė L. Cocksfoot streak virus in Lithuania. *Biologija*. **2001**, *4*, 49-5.<br>Sutic D.D.; Ford, R.E.; Tosic, M.T. Virus diseases of fruit trees. In *Handbook of plant virus diseases,* eds1; CRC Press: Cleveland, OH, USA, 1999; pp. 345–347, ISBN 978-0-8493-2302-7.	*Myzus persicae, Macrosiphum avenae, Macrosiphum euphorbia, Metopolophium dirhodum, Hyalopteroides humulus*	Torrance, L.; Jones, A.T.; Duncan, G.H. Properties of cocksfoot streak and cocksfoot cryptic, two viruses infecting cocksfoot (*Dactylis glomerata*) in Scotland. *Ann. Appl. Biol.* **1994**, *124*, 267-281, doi: 10.1111/j.1744-7348.1994.tb04133.x.<br>Đutic D.D.; Ford, R.E.; Toðic, M.T. *Handbook of Plant Virus Diseases,* 1st ed.; CRS Press: New

					York, USA, 1999; pp. 345-347, ISBN 978-0-8493-2302-7.
Colombian datura virus	IQ801448	Hungary, Poland, Germany, Netherlands, USA, Colombia, Ecuador, Bolivia, India, Australia, Canada., Italy	Salamon, P.; Palkovics, L. Occurrence of Colombian datura virus in Brugmansia hybrids, *Physalis peruviana* L. and *Solanum muricatum* Ait. in Hungary. *Acta Virol.* **2005**, *49*, 117-122.<br>Schubert, J.; Doroszewska, T.; Chrzanowska, M.; Sztangret-Wiś- niewska, J. Natural infection of tobacco by Colombian Datura virus in Poland, Germany, and Hungary. *J. Phytopathol.* **2006**, *154*, 343-348, doi: 10.1111/j.1439-0434.2006.01104.x.<br>Verhoeven, J. Th. J.; Lesemann, D.E.; Roenhorst. J.W. First report of Colombian datura potyvirus in tomato. *Eur. J. Plant Pathol.* **1996**, *102*, 895-898, doi: 10.1007/BF01877061.<br>Fry, C.R.; Zimmerman, M.T.; Scott, S.W. Occurrence of Colombian datura virus in the terrestrial orchid, *Spiranthes cernua*. *J. Phytopathol.* **2004**, *152*, 200-203, doi: 10.1111/j.1439-0434.2004.00827.x.<br>Kahn, R.P.; Bartels, R. The Colombian datura virus – a new virus in the Potato virus Y group. Phytopathology **1968**, *58*, 587-592.<br>Kahn, R.P.; Monroe, R.L. Viruses isolated from arborescent Datura species from Bolivia, Ecuador, and Colombia. *Plant Dis. Rep.* **1970**, *54*, 675-677.<br>Kumar Verma, R.; Mishra, R.; Gaur, R.K. First Report of Colombian datura virus in India. *New Dis. Reports* **2014**, *30*, 29, doi: 10.5197/j.2044-0588.2014.030.029.<br>Steele, V.; Thomas, J.E. First report of Colombian datura virus from Australia. Australas. *Plant Dis. Notes* **2009**, *4*, 108-109, doi: 10.1071/DN09045.<br>Rott, M.; Schmidt, A.M.; Joshi, V.; Masters, C.; Godkin, S.; Johnson, R. First report of Colombian datura virus in Brugmansia in Canada. *Plant Dis.* **2009**, *93*, 196-196, doi: 10.1094/PDIS-93-2-0196B.<br>Pacifico, D.; Crucitti, D.; Stigliano, E.; Ciuffo, M.; Vallino, M.; Carimi, F. First report of Colombian datura virus in *Mandragora autumnalis* in Sicily, Italy. *Plant Dis.* **2016**, *100*, 2338, doi: 10.1094/PDIS-04-16-0445-PDN.	*Myzus persicae*	Kahn, R.P.; Bartels, R. The Colombian datura virus – a new virus in the Potato virus Y group. *Phytopathology* **1968**, *58*, 587-592
Commelina mosaic virus		USA, Dominican Republic	Morales, F.J.; Zettler, F.W. Characterization and eletron microscopy of a potyvirus infecting *Commelina diffusa*. Phytopathology **1977**, *67*, 839-843, doi: 10.1094/Phyto-67-839.<br>Baker, C.A.; Zettler, F.W. Viruses infecting wild and cultivated species of the *Commelinaceae*. *Plant Dis.* **1988**, *72*, 513-518, doi: 10.1094/PD-72-0513.	*Myzus persicae*, *Aphis gossypii*	Morales, F.J.; Zettler, F.W. Characterization and eletron microscopy of a potyvirus infecting Commelina diffusa. *Phytopathology* **1977**, *67*, 839-843.
Cowpea aphid-borne mosaic virus	AF348210	Afghanistan, Bangladesh, China, India, Indonesia, Iran, Iraq, Japan, Malaysia, Nepal, Pakistan, Philippines, Sri Lanka, Singapore, Taiwan, Thailand, Turkey, Botswana, Burkina Faso, Cameroon,	Bashir, M.; Ahmad, Z.; Ghafoor, A. Cowpea aphid-borne mosaic potyvirus: A review. *Internat. J. Pest Manag.* **2002**, *48*, 155-168, doi: 10.1080/09670870110118722.	*Acyrthosiphon pisum*, *Aphis craccivora*, *Aphis fabae*, *Aphis gossypii*, *Aphis medicaginis*, *Aphis spiraecola*, *Macrosiphum euphorbiae*, *Myzus persicae*, *Rhopalosiphum maidis*,	Cabi datasheet on cowpea aphid-borne mosaic virus (CABMV). Available online: https://www.cabi.org/isc/datasheet/15649 (accessed on 24 March 2020).

		Egypt, Ghana, Guinea, Kenya, Morocco, Mozambique, Nigeria, Senegal, South Africa, Tanzania, Togo, Uganda, Zambia, Zimbabwe, Germany, Hungary, Italy, Netherlands, Australia, Papua New Guinea, Brazil, USA		*Aphis craccivora, Aphis fabae, Aphis gossypii, Aphis medicaginis, Aphis spiraecola, Macrosiphum euphorbiae, Myzus persicae, Rhopalosiphum maidis*	
Cucurbit vein banding virus	KY657266	Argentina	Perotto, M.C.; Pozzi, E.A.; Celli, M.G.; Luciani, C.E.; Mitidieri, M.S.; Conci, V.C. Identification and characterization of a new potyvirus infecting cucurbits. *Arch. Virol*. **2018**, *163*, 719-724, doi: 10.1007/s00705-017-3660-2.	Unknown	
Cypripedium virus Y	AF185954	United Kingdom	UK (Unpublished, Genbank Acc. No. AF185954)	Unknown	
Cyrtanthus elatus virus A	JQ723475	India, Japan, Australia, UK	Kumar, S.; Raj, R.; Kaur, C.; Raj, S.K. First report of Cyrtanthus elatus virus A in *Narcissus tazetta* in India. *Plant Dis*. **2015**, *99*, 1658, doi: 10.1094/PDIS-04-15-0492-PDN. Ohshima, K.; Nomiyama, R.; Mitoma, S.; Honda, Y.; Yasaka, R.; Tomimura, K. Evolutionary rates and genetic diversities of mixed potyviruses in Narcissus. *Infect Genet Evol.* **2016**, *45*, 213-223, doi: 10.1016/j.meegid.2016.08.036. Wylie, S.J.; Nouri, S.; Coutts, B.A.; Jones, M.G.K. Narcissus late season yellows virus and vallota speciosa virus found infecting domestic and wild populations of *Narcissus* species in Australia. *Arch. Virol*. **2010**, *155*, 1171-1174, doi: 10.1007/s00705-010-0682-4. Wylie, S.J.; Jones, M.G.K. Complete genome sequences of seven carlavirus and potyvirus isolates from *Narcissus* and *Hippeastrum* plants in Australia, and proposals to clarify their naming. *Arch. Virol*. **2012**, *157*, 1471-1480, doi: 10.1007/s00705-012-1319-6.	Unknown	
Daphne mosaic virus	DQ299908	Czech Republic	Fránová, J.; Petrzik, K.; Lesemann, D.E.; Navrátil, M. Daphne mosaic virus (DapMV), a new potyvirus from *Daphne mezereum* in the Czech Republic. *Arch. Virol*. **2006**, *151*, 793-801, doi: 10.1007/s00705-005-0668-9.	Unknown	
Daphne virus Y	KU556609	New Zealand, Germany, Czech Republic	Forster, R.L.S.; Milne, K.S. Survey of viruses infecting Daphne in New Zealand. *N. Z. J. Agric. Res*. **1975**, *18*, 391-398, doi: 10.1080/00288233.1975.10421066. Petrzik, K.; Fránová, J. Complete genome sequence of daphne mosaic virus—a potyvirus from an ornamental shrub related to papaya leaf distortion mosaic virus. *Arch. Virol*. **2006**, *151*, 1461-1465, doi: 10.1007/s00705-006-0756-5. Park, C.Y.; Park, J.; Lee, B.J.; Bak, S.; Lee, H.K.; Kim, J.S.; Yoon, Y.; Suh, S.J.; Lee, S.H. Identification of daphne mottle virus isolated from *Daphne odora*, a new member of the genus Potyvirus. *Res. Plant Dis*. **2016**, *22*, 59-63, doi: 10.5423/RPD.2016.22.1.59.	*Myzus persicae*	Forster, R.L.S.; Milne, K.S. Survey of viruses infecting Daphne in New Zealand. *N. Z. J. Agric. Res*. **1975**, *18*, 391-398, doi: 10.1080/00288233.1975.10421066.

Dasheen mosaic virus	AJ298033	Ethiopia, USA, India, China, Japan, Venezuela, Puerto Rico, Egypt, Denmark, Netherlands, UK, Nigeria, Solomon Islands, Australia, New Zealand, Bangladesh	Kidanemariam, D.B.; Macharia, M.W.; Harvey, J.; Holton, T.; Sukal, A.; James, A.P.; Harding, R.M.; Abraham, A.D. First report of Dasheen mosaic virus infecting taro (*Colocasia esculenta* L.) from Ethiopia. *Plant Dis*. **2018**, *102*, 1470, doi: 10.1094/PDIS-12-17-1991-PDN.<br>Zettler, F.W.; Foxe, M.J.; Hartman, R.D.; Edwardson, J.R.; Christie, R.G. Filamentous viruses infecting dasheen and other araceous plants. *Phytopathology* **1970**, *60*, 983–987, doi: 10.1094/Phyto-60-983.<br>Ram, R.; Joshi, A.; Verma, N.; Kulshrestha, S.; Raikhy, G.; Hallan, V.; Zaidi, A.A. First report of dasheen mosaic virus infecting four ornamental aroids in India. *Plant Pathol.* **2003,** *52*, 411, doi: 10.1046/j.1365-3059.2003.00836.x.<br>Chen, J.; Chen, J.P.; Chen, J.S.; Adams, M.J. Molecular characterization of an isolate of dasheen mosaic virus from *Zantedeschia aethiopica* in China and comparisons in the genus Potyvirus. *Arch. Virol.* **2001**, *146*, 1821-1829, doi: 10.1007/s007050170068.<br>Debrot, E.A.; Ordosgoitti, A. Dasheen mosaic virus infection in *colocasia* and *Xanthosoma* in Venenzulla. *Pl. Dis. Reptr.* **1974,** *58*, 1032<br>Alconero, R.; Zettler, F.W. Virus infection in Colocasia and Xanthosoma in Puerto Rico. *Pl. Dis. Reptr.* **1971**, *55*: 506<br>El Nil, M.M.A.; Zettler, F.W. Natural accurance of dasheen mosaic virus in Egyptian taro (*Colocasia Antiquorum*). *Pl. Dis. Reptr.* **1976**, *60*, 281,<br>Hakkaart, F.A.; Waterreus, H.A.J.I. A virus disease of Dieffenbachia 'Exotica'. *Acta Horticult*. **1976**, *59*, 175-180, doi: 10.17660/ActaHortic.1976.59.24.<br>Van Hoof. Jversl. Inst. plziektenk. Onderz. Wageningen, 1971; 87.<br>Zettler, F.W.; Abo EL Nil, M.M.; Hartman, R.D. *Dasheen mosaic virus*. *Description of plant viruses.* Commonwealth Mycological Institute/Association Bio. Kew, Survey, UK, 1978**,** No.191, pp.4.<br>Vollin, B.R.; Thottappilly, G.; Rossel, H.W.; Terry, E.R. Isolation of dasheen mosaic virus in Nigeria and screening cocayam seedling for resistance (Abts). *Phytopathology* **1981**, *71*, 263.<br>Gollifer, D.E.; Jackson, G.V.H.; Dabek, A.J.; Plumb, R.T.; May, Y.Y. The occurrence and transmission of viruses of edible aroids in the Solomon Islands and the Southwest Pacific. *Intj. J. Pest. Manage***. 2009,** *23*, 171-177, doi: 10.1080/09670877709412425.<br>Greber, R.S.; Shaw, D.E. Dasheen mosaic virus in Queensland. *Austral. Plant Pathol.* **1986,** *15*, 29-33, doi: 10.1071/APP9860029.<br>Pearson, M.N.; Bussell, W.T.; Scheffer, J. J. C. New plant disease record in New Zealand: Dasheen mosaic potyvirus infecting taro (*Colocasia esculenta* Schott). *N. Z. J. Crop. Hortic. Sci.* **1998**, *26*, 69-70, doi: 10.1080/01140671.1998.9514042.<br>Wang, D.; Hamim, I.; Borth, W.B.; Melzer, M.J.; Sun, G.F.; Hu, J.S. First report of Dasheen mosaic virus infecting taro (*Colocasia esculenta* L.) in Bangladesh. *Plant Dis*. **2018**, *102*, 2668, doi: 10.1094/PDIS-03-18-0442-PDN.	*Myzus persicae, Aphis craccivora*	Zettler, F.W.; Foxe, M.J.; Hartman, R.D.; Edwardson, J.R.; Christie, R.G. Filamentous viruses infecting dasheen and other araceous plants. *Phytopathology* **1970**, *60*, 983–987, doi: 10.1094/Phyto-60-983.

Datura shoestring virus		India	Giri, L.; Agrawal, H.O. Datura shoestring virus, a new virus isolated from *Solanum jasminoides*. *Phytopathology* **1971**, *70*, 80-83, doi: 10.1111/j.1439-0434.1971.tb03465.x.	Unknown	
Diuris virus Y	AF203527	Australia	Gibbs, A.; Mackenzie, A.; Blanchfield, A.; Cross, P.; Wilson, C.; Kitajima, E.; Nightingale, M.; Clements, M. Viruses of orchids in Australia; their identification, biology and control. *Aust. Orchid Rev.* **2000,** *65*, 10-21.	Unknown	
Donkey orchid virus A	JX156422	Australia	Wylie, S. J.; Li, H.; Dixon, K.W.; Richards, H.; Jones, M.G.K. Exotic and indigenous viruses infect wild populations and captive collections of temperate terrestrial orchids (*Diuris species*) in Australia. *Virus Res*., **2013**, *171*, 22-32, doi: 10.1016/j.virusres.2012.10.003.	Unknown	
East Asian Passiflora virus	AB246773	Japan, Malaysia, Uganda, Taiwan	Iwai, H.; Yamashita, Y.; Nishi, N.; Nakamura, M. The potyvirus associated with the dappled fruit of *Passiflora edulis* in Kagoshima prefecture, Japan is the third strain of the proposed new species East Asian passiflora virus (EAPV) phylogenetically distinguished from strains of passion fruit woodiness virus. *Arch. Virol.* **2006**, *151*, 811-818, doi: 10.1007/s00705-005-0659-x. Abdullah, N.; Ismail, I.; Pillai, V.; Abdullah, R.; Sharifudin, A.S. Nucleotide sequence of the coat protein gene of the Malaysian passiflora virus and its 3′ non-coding region. *Am. J. Appl. Sci.* **2009**, *6*, 1633-1636, doi: 10.3844/ajassp.2009.1633.1636. Ochwo-Ssemakula, M.; Sengooba, T.; Hakiza, J.J.; Adipala, E.; Edema, R.; Redinbaugh, M.G.; Aritua, V.; Winter, S. Characterization and distribution of a potyvirus associated with passion fruit woodiness disease in Uganda. *Plant Dis.* **2012**, *96*, 659-665, doi: 10.1094/PDIS-03-11-0263. Chong, Y.H.; Cheng, Y.H.; Cheng, H.W.; Huang, Y,C.; Yeh, S.D. The virus causing passionfruit woodiness disease in Taiwan is reclassified as East Asian passiflora virus. *J. Gen. Pl. Path*, **2018,** *84*, 208-220, doi: 10.1007/s10327-018-0777-4.	*Aphis gossypii, Hyperomyzus lactucae, Myzus persicae*	Omatsu, N.; Iwai, H.; Setokuchi, O.; Arai, K. Immigrating aphid species and their importance as vectors of passionfruit woodiness virus in the fields of Amami Oshima Island, Japan. Memoirs of the Faculty of Agriculture, Kagoshima University, **2004**, *39*, 1-5. Iwai, H.; Ohmori, T.; Kurokawa, Y.; Muta, T.; Arai, K. New record of passionfruit woodiness virus in Japan. *Ann. Phytopathol. Soc. Jpn.* **1996**, *62*, 459-465, doi: 10.3186/jjphytopath.62.459.
Endive necrotic mosaic virus	AJ223827	France, Germany, South Africa	Tepfer, M.; Verdin, E.; Wipf-Scheibel, C.; Moury, B. Molecular and biological characterization of two potyviruses infecting lettuce in southeastern France. *Plant Pathol.* **2016,** *66*, 970-979, doi: 10.1111/ppa.12651. Blancard, D.; Lot, H.; Maisonneuve, B. *A Color Atlas of Diseases of Lettuce and Related Salad Crops. Observation, Biology and Control,* 1st ed.; Academic Press: Boston, MA, USA, 2006; ISBN-10:0123725577. Jooste, A.E.C.; Kasdorf, G.G.F.; Van der Merwe, M. First report of endive necrotic mosaic virus on lettuce in South Africa. **2011** (Unpublished, GenBank Acc. No. JQ437577).	*Myzus persicae*	Tepfer, M.; Verdin, E.; Wipf-Scheibel, C.; Moury, B. Molecular and biological characterization of two potyviruses infecting lettuce in southeastern France. *Plant Pathol.* **2016**, *66*, 970-979 doi : 10.1111/ppa.1265.
Euphorbia ringspot virus	AY697300	Germany, Australia, USA, Venezuela	Bode. O.; Lesemann, D.E. Euphorbia ringspot virus, a new virus in *Euphorbia milii x lophogna. Acta. Hort.* **1976**, *59*,161-166, doi: 10.17660/ActaHortic.1976.59.22. Gibbs, A.J.; Mackenzie, A.M.; Wei, K.J.; Gibbs, M.J. The potyviruses of Australia. *Arch. Virol.* **2008**, *153*, 1411-1420, doi: 10.1007/s00705-008-0134-6. Guaragna, M.A.; Ambrose, J.; Jordan, R.L. Detection and characterization of euphorbia ringspot virus. *Phytopathology* **2004**, *94*, S36.	*Myzus persicae*	Bode, O.; Lesemann, D.E. Euphorbia ringspot virus, a new virus in *Euphorbia milii x lophogna*. *Acta Hort.* **1976,** *59*, 161-166, doi: 10.17660/ActaHortic.1976.59.22.

			Marys, E.; Romano, M. Occurrence of euphorbia ringspot virus in *Euphorbia milii cv. Splendens* in Venezuela. *J Phytopathol*. **2011**, *159*, 66-68, doi: 10.1111/j.1439-0434.2010.01709.x.		
Freesia mosaic virus	FM206346	Netherlands, Australia Ireland, Netherlands, UK, India, Korea	Van Koot, Y.; van Slogteren, D.H.M.; Cremer, M.C.; Camfferman, J. Virus verschijn-selen in Freesia's. *Tijdschr Plziekt* **1954,** 60, 157. Brunt, A.A. Major Genera of Plant Viruses. In *virus and virus-like diseases of bulb and flower crops,* 1st ed.; Loebenstein G., Lawson, R.H., Brunt, A.A. Eds.; Wiley: Chichester, UK, 1996; pp 274–280, ISBN: 978-0-471-95293-0. Kumar, Y.; Hallan, V.; Zaidi, A.A. First finding of freesia mosaic virus infecting freesia in India. *Plant Pathol.* **2009**, *58*, 404, doi: 10.1111/j.1365-3059.2008.01995.x. Jeong, M.I.; Choi, Y.J.; Joa, J.H.; Choi, K.S.; Chung, B.N. First report of Freesia sneak virus in commercial Freesia hybrida cultivars in Korea. *Plant Dis.* **2014**, *95*,162, doi: 10.1094/PDIS-05-13-0484-PDN.	*Macrosiphum euphorbiae, Myzus persicae*	Available online:https://www.pacificbulbsociety.org/pbswiki/index.php/Virus (accessed on 30 March 2020).
Fritillary virus Y	AM039800	China	Chen, J.; Zheng, H.Y.; Shi, Y.H.; Adams, M.J.; Wei, C.B.; Lin, L.; Chen, J.P. Detection and characterization of a second potyvirus from *Thunberg fritillary* in China. *Arch. Virol.* **2006**, *151*, 439-447, doi: 10.1007/s00705-005-0678-7.	Unknown	
Gloriosa stripe mosaic virus	EF427894	Taiwan, USA, Brazil, Japan, Netherlands	Chen, C.C.; Chiang, F.L.; Huang, C.H. Molecular and serological identification of Gloriosa stripe mosaic virus on Christmas bells (*Sandersonia aurantiaca* Hook). *J. Taiwan Agric. Res.* **2018**, *67*, 229-246. Mollov, D.; Grinstead, S.; Tahir, M.N.; Reinsel, M.; Hammond. J. First report of multiple isolates of gloriosa stripe mosaic virus in *Gloriosa superba* in the United States. Plant *Dis.* **2017**, *101*,1070, doi: 10.1094/PDIS-12-16-1795-PDN. Duarte, L.M.L.; Alexandre, M.A.V.; Chaves, A.L.R.; Harakava, R.; Alvim, L.G.B.; Kitajima, E.W.; Bianchini, L.; Silva, S.C.M.; Neves, V.B.S. Widespread incidence of Gloriosa stripe mosaic virus infecting *Gloriosa superba* in São Paulo state, Brazil. *Trop. plant pathol.* **2019,** *44*, 297, doi: 10.1007/s40858-018-0265-6. Araki, M.; Yamashita, S.; Doi, Y.; Yora, K. Three viruses from gloriosa (*Gloriosa rothschildiana O'brien*): gloriosa fleck virus, gloriosa stripe mosaic virus and cucumber mosaic virus. *Ann. Phytopathol. Soc. Jpn.* **1985**, *51*, 632–636. Pham, K.T.K.; Kock, M.J.D.; Lemmers, M.E.C.; Derks, A.F.L.M. Molecular identification of potyviruses infecting ornamentals bulbs by the analysis of coat protein (CP) sequences. *Acta Hort.* **2011**, *901*, 167-172, doi: 10.17660/ActaHortic.2011.901.21.	Unknown	
Habenaria mosaic virus	AB818538	South Korea, Japan	Igori, D.; Lim, S.; Zhao, F.; Baek, D.; Moon, J.S. Complete genome sequence of a South Korean isolate of habenaria mosaic virus. *Genome Announc.* **2016**, *4*, e00958, doi: 10.1128/genomeA.00958-16. Gara, I.W.; Kondo, H.; Maeda, T.; Inouye, N.; Tamada, T. Stunt disease of *Habenaria radiata* caused by a strain of watermelon mosaic virus. *Ann. Phytopathl. Soc. Jpn.* **1997**, *63,* 113–117.	*Myzus persicae*	Gara, I.W.; Kondo, H.; Maeda, T.; Inouye, N.; Tamada, T. Stunt disease of *Habenaria radiata*caused by a strain of *Watermelon mosaic virus. Ann. Phytopathl. Soc. Jpn.* **1997**, *63*, 113-117
Hardenbergia mosaic virus	HQ161081	Australia	Webster, C.G. Characterization of Hardenbergia Mosaic Virus and development of microarrays for detecting viruses in plants. PhD thesis, Murduch University, Perth, WA, Australia, 2008.	*Myzus persicae*	Webster, C.G. Characterization of Hardenbergia Mosaic Virus and development of microarrays for detecting viruses in plants.

					PhD thesis, Murduch University, Perth, WA, Australia, 2008.
Henbane mosaic virus	AM184113	United Kingdom, Italy, Hungary, Germany	Hamilton, A.M. On three new virus diseases of *Hyoscyamus niger*. *Ann. Appl. Biol.* **1932**, *19*, 550-567. doi: 10.1111/j.1744-7348.1932.tb04341.x. Lovisolo, O.; Bartels, R. On a new strain of henbane mosaic virus from physalis alkekengi. *J. Phytopathol.* **1970**, *69*, 189–201. doi: 10.1111/j.1439-0434.1970.tb03102.x. Horváth, J.; Salamon, P.; Wolf, I.; Kölber, M. Henbane mosaic potyvirus pathogenic to wild and cultivated potato. *Potato Res.* **1988**, *31*, 311–320. doi: 10.1007/BF02365540. Lovisolo, O. Ecology and taxonomy of some European potyviruses. *Arch. Virol. Suppl.* **1992**, *5*, 311-316. doi: 10.1007/978-3-7091-6920-9_32.	*Myzus persicae, Myzus ascalonicus, Aulacorthum circumflexum, Macrosiphum euphorbiae*	Watson, M.A.; Roberts, F.M. A comparative study of the transmission of Hyocyamus virus 3, potato virus Y and cucumber virus 1 by the vectors *Myzus persicae* (Sulz.), *M. circumflexus* (Buckton), and *Macrosiphum euphorbiae* (Koch). *Proc. R. Soc. London Ser. B*. **1939**, *127*, 543–76. Doncaster, I.P.; Kassanis, B. The shallot aphis, *Myzus ascalonicus* Doncaster, and its behaviour as a vector of plant viruses. *Ann. Appl. Bioi.* **1946**, *33*, 66-68.
Hibbertia virus Y	AF228516	Australia	Gibbs, A.; Mackenzie, A. A primer pair for amplifying part of the genome of all potyvirids by RT-PCR. *J. Virol. Meth.* **1997**, *63*, 9-16, doi: 10.1016/s0166-0934(96)02103-9.	Unknown	
Hippeastrum mosaic virus	IQ395040	Netherlands, Brazil, India, New Zealand, China, Bulgaria, Italy, Thailand, USA, Japan	Hendrina Brants, D.; van den Heuvel, J. Investigation of hippeastrum mosaic virus in *Hippeastrum hybridum* . *Neth. J. Plant Pathol.* **1965**, *71*, 145-151. Alexandre, M.A.V.; Duarte, L.M.L.; Rivas, E.B.; Cilli, A.; Harakava, R.; Galleti, S.R.; Kitajima, E.W. Hippeastrum mosaic virus diagnosed in hippeastrum and eucharis in Brazil. *J. Plant Pathol.* **2011**, *93*, 643-649, doi: 10.4454/jpp.v93i3.1231. Raj, S.K.; Snehi, S.K.; Kumar, S.; Khan, M.S. First molecular detection and identification of a potyvirus associated with severe mosaic disease of amaryllis (*Hippeastrum hybridum* Hort.) in India. *Australas. Plant Dis. Notes.* **2009**, *4*, 50-53, doi: 10.1071/DN09021. Pearson, M.N; Cohen, D.; Cowell, S.J.; Jones, D.; Blouin, A.; Lebas, B.S.M.; Shiller, J.B.; Clover, G.R.C. A survey of viruses of flower bulbs in New Zealand. *Australas. Plant Pathol.* **2009**, *38*, 305-309, doi: doi.org/10.1071/AP09006. Xu, X. H.; Tang, W.; Gao, R.; Yang, S.K.; Li, F.; Sun, H.W.; Lu, X.B. First Report of hippeastrum mosaic virus in *Hippeastrum spp.* in Mainland China. *Plant Dis.* **2017**, *101*, 1064-1064, doi: 10.1094/PDIS-08-16-1109-PDN. Bakardjieva, N.; Denkova, S. 2014. Hippeastrum Mosaic Virus Isolated in Bulgaria. *Biotechnol. Biotechnol. Equip*. **2014**, *10*, 41-43, doi.org/10.1080/13102818.1996.10818879. Castellano, M.A.; Rana, G.L.; Bari. Occurrence of hippeastrum mosaic virus in Apulia (Italy). *Agris. fao. org*. **1983**, *32*, 37-39. Korpraditskul, P.S.P. Detection of virus diseases (hippeastrum mosaic virus) in *Amaryllis sp.* by using IEM in Thailand. **1980**. Conference paper. Holmes, F.O. Cytological study of the intracellular body characteristic of hippeastrum mosaic. *Bot. Gaz.* **1928**, *86*, 50-58, doi: 10.1086/333871. Iwaki, M. Viruses causing mosaic diseases of Amaryllis in Japan. *Ann. phytopath. Soc. Japan* **1967**, *33*, 237–243.	*Aphis gossypii, Myzus persica*	Brunt, A.A. Iris severe mosaic virus. *A. Rep. Glasshouse Crops Res. Inst.* **1972**, 103-104.

Hyacinth mosaic virus	KY828925	Bulgaria, USA, Australia, Lithuania, Netherlands, UK, Brazil	Brunt, A.A.; Crabtree, K.; Dallwitz, M.J.; Gibbs, A.J.; Watson, L. *Viruses of plants-descriptions and lists from the VIDE database*. CAB International: Wallingford, UK, **1997**; pp. 675-676, ISBN 0851 98794X.<br>Alexandre, M.A.V.; Duarte, M.L.; Rodrigues, L.K.; Ramos, A.F.; Harakava, R. Hyacinth mosaic virus infecting *Hyacinthus sp.* plants in Brazil. *Trop. plant pathol.* **2017**, *42*, 51-54, doi: 10.1007/s40858-016-0121-5.	Unknown	
Impatiens flower break virus	KU981084	South Korea, USA	Cho, S.Y.; Kim, H.; Yi, S.I.; Lim, S.; Park, J.M.; Cho, H.S.; Kim, H.; Kwon, S.Y.; Moon, J.S. First Report of impatiens flower break virus infecting impatiens walleriana in South Korea. *Plant Dis.* **2016**, *2*, 394-394, doi: 10.1094/PDIS-08-16-1192-PDN.<br>Jordan, R.L.; Guaragna, M.A.; Putan, M. Detection and molecular characterization of new and emerging potyviruses of ornamental plants. *Acta horti.* **2011**, *901*, 159-166, doi: 10.17660/ActaHortic.2011.901.20.	Unknown	
Iris fulva mosaic virus		USA	Barnett, O.W.; Alper, M. Characterization of Iris fulva mosaic virus. *Phytopathology* **1977**, *67*, 448-454, doi: 10.1094/Phyto-67-448.	*Myzus persicae,*	Barnett, O.W.; Alper, M. Characterization of Iris fulva mosaic virus. *Phytopathology* **1977**, *67*, 448-454, doi: 10.1094/Phyto-67-448.
Iris mild mosaic virus	JF320812	Japan, Israel, Britain, Netherlands, India	Inouye, N.; Miyaji, K.; Mitsuhata, K. Host range and some properties of iris severe mosaic virus found in Japan. *Japan J. Phytopath.* **1981,** *47*, 182-188, (In Japanese with English summary) doi: 10.3186/jjphytopath.<br>Loebenstein, G.; Alper, M. Two viruses diseases of irises in Israel. *Phytopathology* **1963**, *53*, 349-350.<br>Derks, A.F.L.M.; Hollinger, T.H.C.; Vink-van den Abeele, J.L. Identification and symptom expression of four elongated viruses infecting bulbous irises. *Acta Hort.* **1985**, *164*, 309-318, doi: 10.17660/ActaHortic.1985.164.34.<br>Asjes, N. Viruses and diseases in the Dutch bulbous irises (*Iris hollandico*) in the Netherlands. *J. Pl. Path.* **1979**, *85*: 269. doi: 10.1007/BF01977599.<br>Kulshrestha, S.; Hallan, V.; Raikhy, G.; Ram R.; Garg, I.; Haq, Q.M.R.; Zaidi, A.A. Occurrence of iris mild mosaic potyvirus in cultivated iris in India. *Ind. J. Biotechnol.* **2006**, *5*, 94-98.	*Aphis gossypii, Macrosiphum euphorbiae, Myzus persicae*	Loebenstein, G.; Alper, M. Two viruses diseases of irises in Israel. *Phytopathology* **1963**, *53*, 349-350.<br>Inouye, N.; Miyaji, K.; Mitsuhata, K. Iris Severe mosaic virus. *Ann. Phytopath. Soc. Jpn.* **1981**, *47*, 182-188.
Iris severe mosaic virus	KT692938	Netherlands, China, Iran, India, South Korea, Japan	Brunt, A.A.; Derks, A.F.L.M.; Barnett, O.W. *Iris severe mosaic virus*. Descriptions of plant viruses / prepared by the Commonwealth Mycological Institute and Association of Applied Biology, Commonwealth Mycological institute/Association of Applied Biologists.; Kew (Surrey), UK, **1970;** *338*, pp. 5.<br>Li, Y.; Deng, C.; Shang, Q.; Zhao, X.; Liu, X.; Zhou, Q. The first complete genome sequence of iris severe mosaic virus. *Arch. Virol.* **2016**, *161*, 1069-1072, doi: 10.1007/s00705-015-2743-1.<br>Nateqi, M.; Habibi, M.K.; Dizadji, A.; Parizad, S. Detection and molecular characterization of the iris severe mosaic virus-isolate from Iran. *J. Plant Prot. Res.* **2015,** *55*, 235-240, doi: 10.1515/jppr-2015-0032.<br>Kulshrestha, S.; Hallan, V.; Raikhy, G.; Kumar, A.; Ram, R.; Garg, I. D.; Zaidi, A.A. Molecular characterization of an iris severe mosaic virus isolate from India. *Acta Virol.* **2004**, *48*, 65–67	*Macrosiphum euphorbiae, Myzus persicae*	Asjes, C.J. Viruses and virus diseases in Dutch bulbous irises (*Iris hollandica*) in the Netherlands. *Neth. J. Plant Pathol.* **1979**, *85*, 269-279, doi: 10.1007/BF01977599<br>Brierley, P.; McWhorter, F.P. A mosaic disease of Iris. *J. Agric. Res.* **1936**, *53*, 621-635.

			Park, W.M.; Lee, S.S.; Park, S.H.; Ryu, K.H. Sequence analysis of the coat protein gene of a Korean isolate of iris severe mosaic potyvirus from iris plant. *Plant Pathol. J.* **2000**, *16*, 36–42, doi: dx.doi.org.<br>Nishikawa, M.; Tokuda, R.; Yoshida, T.; Nijo, T.; Maruyama, N.; Katsu, K.; Maejima, K.; Yamaji, Y.; Namba, S. Complete genome sequence of iris severe mosaic virus isolated in Japan. *Microbiol Resour. Announc.* **2019**, *8*, e00093-19, doi: 10.1128/MRA.00093-19.		
Japanese yam mosaic virus	AB027007	Japan, China	Fuji, S.; Nakamae, H. Complete nucleotide sequence of the genomic RNA of a Japanese yam mosaic virus, a new potyvirus in Japan. *Arch Virol.* **1999**, *144*, 231-240, doi: 10.1007/s007050050500.<br>Lan, P.; Li, F.; Wang, M.; Li, R. Complete genome sequence of a divergent strain of Japanese yam mosaic virus from China. *Arch Virol.* **2015**, *160*, 573-576, doi: 10.1007/s00705-014-2231-z.	*Aphis gossypii, Myzus persicae*	Okuyama, S.; Saka, H. Yam mosaic virus. *Sci. Rep. Fac. Agr. Ibaraki. Univ.* **1978**, *26*, 29–34.
Jasmine virus T	KT222674	China, Taiwan	Tang, Y.; Gao, F.; Yang, Z.; Wu, Z.; Yang, L. Complete genome analysis of jasmine virus T from *Jasminum sambac* in China. *Arch Virol.* **2016**, *161*, 2033-2036, 10.1007/s00705-016-2811-1.<br>Chang, C.A.; Lin, Y.C.; Lin, Y.Y.; Wang, S.M. Etiological study of viruses associated with yellow mosaic disease in jasmine. *Acta horticul.* **2018**, *1193*, 47-54, doi: 10.17660/ActaHortic.2018.1193.7.	Unknown	
Johnsongrass mosaic virus	Z26920	Australia, USA, Venezuela, Brazil, Kenya, Uganda, Rwanda, Tanzania	Taylor, R.H.; Pares, R.D. The relationship between sugar-cane mosaic virus and mosaic viruses of maize and Johnson grass in Australia. *Aust. J. Agric. Res.* **1968,** *19*, 767-773, doi: 10.1071/AR9680767.<br>McDaniel, L.L.; Gordon, D.T. Identification of a new strain of maize dwarf mosaic virus. *Plant Dis.* **1985**, *69*, 602-607, doi: 10.1094/PD-69-602.<br>Garrido, M.J.; Trujillo, G.E. Occurrence of Johsongrass mosaic virus on sorghum in Venezuela. *Plant Dis.* **1993**, 77, 847, doi: 10.1094/PD-77-0847A.<br>Camelo-Garcıa, V.M.; Andrade, S.C.D.; Geering, A.D.W.; Kitajima, E.W.; Rezende, J.A.M. Genome organization and host range of a Brazilian isolate of Johnsongrass mosaic virus. *Arch. Virol.* **2016**, *161*, 1335-1341, doi: 10.1007/s00705-016-2772-4.<br>Stewart, L.R.; Willie, K.; Wijeratne, S.; Redinbaugh, M.G.; Massawe, D.; Niblett, C.L.; Kiggundu, A.; Asiimwe, T. Johnsongrass mosaic virus contributes to maize lethal necrosis in East Africa. *Plant Dis.* **2017**, *101*, 1455-62, doi: 10.1094/PDIS-01-17-0136-RE.	*Aphis craccivora, Aphis gossypii, Myzus persicae, Rhopalosiphum maidis*	Teakle, D.S.; Grylls, N.E. Four strains of sugarcane mosaic virus infecting cereals and other grasses in Australia. *Austral. J. Agri. Res.* **1973**, *24*, 465-477, doi: 10.1071/AR9730465.
Kalanchoe mosaic virus	GQ497731	Italy, Netherlands, USA, Denmark, Norway	Sorrentino, R.; Marais, A.; Faure, C.; Theil, S.; Alioto, D.; Candresse, T. First Report of kalanchoe mosaic virus and kalanchoe latent virus infecting ghost plant (*Graptopetalum paraguayense*) in Italy. *Plant Dis.* **2017**, *101*, 1560-1560, 0.1094/PDIS-03-17-0338-PDN.<br>Netherlands, (Unpublished, Genbank Acc. No. GQ497732).<br>Husted, K.; Bech, K.; Albrechtsen, M.; Borkhardt, B. Identification, partial sequencing, and detection of a potyvirus from *Kalanchoe blossfeldiana*. *Phytopathology* **1994**, *84*,161-166, doi: 10.1094/phyto-84-161.	*Myzus persicae*	Husted, K.; Bech, K.; Albrechtsen, M.; Borkhardt, B. Identification, partial sequencing, and detection of a potyvirus from *Kalanchoe blossfeldiana*. *Phytopathology* **1994**, *84*, 161-166, doi: 10.1094/phyto-84-161.

			Bjørke, L.; Willumsen, K.; Skjeseth, G.; Haugslien, S.; Blystad, D.R. Differences in yield of cuttings and cultivation performance between virus-free and virus-infected plants of *kalanchoe blossfeldiana*. *Acta Hortic.* **2015**, *1072*, 91-96, doi: 10.17660/ActaHortic.2015.1072.9.		
Keunjorong mosaic virus	JF838187	South Korea	Nam, M.; Lee, J.; Choi, H.S.; Lim, H.S.; Moon, J.S.; Lee, S.H.Complete genome sequence of keunjorong mosaic virus, a potyvirus from *Cynanchum wilfordii*. *Arch Virol.* **2013**, *158*, 1817-1820, doi: 10.1007/s00705-013-1652-4.	Unknown	
Konjac mosaic virus	AB219545	Japan, Hungary, India, Brazil, China, Germany, Netherlands	Shimoyama, J.; Kameya-Iwaki, M.; Hanada. K.; Gunji, T. Konjac mosaic virus, a new potyvirus infecting konjac, *Amorphophallus konjac*. *Ann. Phytopathol. Soc. Jpn.* **1992**, *58*, 706-712, doi: 10.3186/jjphytopath.58.706. Padmavathi, M.; Srinivas, K. P.; Hema, M.; Sreenivasulu, P. First report of Konjac mosaic virus in elephant foot yam (*Amorphophallus paeoniifolius*) from India. *Australas. Plant Dis. Notes*, **2013**, *8*, 27–29, doi: 10.1007/s13314-012-0088-0. Ágoston, J.; Almási, A.; Salánki, K.; Palkovics, L. First report of konjac mosaic virus in Zantedeschia from Hungary. *J. Plant Pathol.* **2019**, *101*, 1217, doi: 10.1007/s42161-019-00279-6. Alexandre, M.A.V.; Duarte, L.M.L.; Rivas, E.B.; Kitajima, E.W.; Harakava, R. First report of konjac mosaic virus in *Zamioculcas zamiifolia*. *Plant Dis.* **2013**, *97*, 1517, doi: 10.1094/PDIS-05-13-0537-PDN. Shi, Y.; Hong, X.; Chen, J.; Adams, M.J.; Zheng, H.Y.; Lin, L.; Qin, B.X.; Chen, J.P. Further molecular characterization of potyviruses infecting aroid plants for medicinal use in China. *Arch Virol.* **2005**, *150*, 125–135, doi: 10.1007/s00705-004-0390-z. Lesemann, D.E.; Winter, S. Konjac mosaic virus, dasheen mosaic virus and unknown potyviruses infecting *Zantedeschia spp.* and other cultivated *Araceae*. *Acta Hortic.* **2002**, *568*, 135-141, doi: 10.17660/ActaHortic.2002.568.19. Pham, K.; Langeveld, S.A.; Lemmers, M.E.C.; Derks, A.F.L.M. Detection and identification of potyviruses in *Zantedeschia*. *Acta Hortic.* 2002, *568*, 143-148, doi: 10.17660/ActaHortic.2002.568.20.	*Aphis gossypi*	Shimoyama, J.; Kameya-Iwaki, M.; Hanada, K.; Gunji, T. Konjak mosaic virus, a new potyvirus infecting konjak, *Amorphophallus konjac*. *Ann. Phytopathol. Soc. Jpn.* **1992**, *58*, 706-712, doi: 10.3186/jjphytopath.58.706.
Leek yellow stripe virus	AJ307057	USA, Ecuador, Croatia, Serbia, Turkey, India, Poland, Egypt, Netherlands, Argentina, USA, China, Vietnam, Brazil, Morocco, Egypt, France, Slovenia, Israel, Greece, Syria, Australia, New Zealand, Bangladesh, China, Japan, Indonesia, Iran,	Oleas, A.; Arahana, V. First report of leek yellow stripe virus, shallot latent virus, and onion yellow dwarf virus in garlic from Ecuador. *Plant Dis.* **2016**, *100*, 232, doi: 10.1094/PDIS-03-15-0318-PDN. Vončina, D.; Ćurić, K.; Fabek, S.; Toth, N. First report of onion yellow dwarf virus, leek yellow stripe virus, and garlic common latent virus on garlic in Croatia. *Plant Dis.* **2016**, *100*, 656, doi: 10.1094/PDIS-08-15-0952-PDN. Vučurović, I.; Vučurović, A.; Nikolić, D.; Bulajić, A.; Milošević, D.; Krstić, B.; Stanković, I. First report of leek yellow stripe virus in leek in Serbia. *Plant Dis.* **2016,** *100*, 230, doi: 10.1094/PDIS-04-15-0432-PDN. Fidan, H.; Baloglu, S. First report of onion yellow dwarf virus and leek yellow stripe virus in garlic in Turkey. *Plant Dis.* **2009**, *93*, 672, doi: 10.1094/PDIS-93-6-0672C.	*Aphis fabae*, *Myzus persicae*, *Rhopalosiphum padi*, *Rhopalosiphum maidis*, *Schizaphis graminum*, *Aphis gossypii*, *Aphis nerii*, *Uroleucon sonchi*, *Hyperomyzus carduellinus*, *Aphis craccivora*, *Acyrthosiphon pisum*, *Dactynotus sonchi*	Verhoyen, M.; Horvat, F. La striurechlorotique du porreau. 1. Identificatin de l'agent causal. *Parasitica*, **1973**, *29*, 16-28. Bos, L.; Huijberts, N.; Huttinga, H.; Maat, D.Z. Leek yellow stripe virus and its relationships to onion yellow dwarf virus; characterization, ecology and possible control. *Neth. J. Plant Pathol.* **1978**, *84*, 185-204, doi:10.1007/BF02650386. Lunello, P.; Ducasse, D.A.; Helguera, M.; Nome, S.F.; Conci, V.C. An Argentinean isolate of leek yellow stripe virus from leek can be transmitted to garlic. *J. Plant Pathol.* **2002**, 84, 11-17, doi: 10.4454/jpp.v84i1.1082.

	Thailand, Turkey, Vietnam, Yemen. Belgium, Czech Republic, Denmark, Finland, France, Germany, Greece, Italy, Netherlands, Sweden, Australia, New Zealand, Mexico, United States, Argentina, Brazil, Chile, Colombia, Uruguay, Venezuela	Gupta, N.; Prabha, K.; Islam, S.; Baranwal, V.K. First report of leek yellow stripe virus in garlic from India. *J. Plant Pathol.* **2013**, *95*, 69-77, doi: 10.4454/JPP.V95I4.017.<br>Chodorska, M.; Paduch-Cichal, E.; Kalinowska, E.; Gaczkowska, O.; Lis, M.; Sierant, B.; Szyndel, M.S. First report of leek yellow stripe virus in foreign and polish garlic plants in central Poland. *J. Plant Pathol.* **2014**, *96*, 4-120, doi: 10.4454/JPP.V96I4.015.<br>Abd El-Wahab, A.S. Aphid-transmission efficiency of two main viruses on garlic in Egypt, onion yellow dwarf virus (OYDV-G) and leek yellow stripe virus (LYSV-G). *Aca. J Entomol.* **2009**, *2*, 40-42.<br>Bos, L.; Huijberts, N.; Huttinga, H.; Maat, D.Z. Leek yellow stripe virus and its relationships to onion yellow dwarf virus; characterization, ecology and possible control. *Neth. J. Plant Pathol.* **1978**, *84*, 185-204. doi:10.1007/BF02650386.<br>Conci, V.C.; Lunello, P.; Buraschi, D.; Italia, R.R.; Nome, S.F. Variations of leek yellow stripe virus concentration in garlic and its incidence in Argentina. *Plant Dis.* **2002**, *86*, 1085-1088, doi: 10.1094/PDIS.2002.86.10.1085.<br>Lot, H.; Chovelon, V.; Souche, S.; Delecolle, B. Effects of onion yellow dwarf and leek yellow stripe viruses on symptomatology and yield loss of three French garlic cultivars. *Plant Dis.* **1998**, *82*, 1381-1385, doi: 10.1094/PDIS.1998.82.12.1381.<br>Pappu, H.R.; Hellier, B.C.; Dugan, F.M. First report of onion yellow dwarf virus, leek yellow stripe virus, and garlic common latent virus in garlic in Washington State. *Plant Dis.* **2005**, *89*, 205, doi: 10.1094/PD-89-0205C.<br>Chen, J.; Chen, J.P.; Adams, M.J. Characterization of some carla- and potyviruses from bulb crops in China. (Brief report). *Arch. Virol.* **2002**, *147*, 419-428, doi: 10.1007/s705-002-8330-y.<br>Ha, C.; Revill, P.; Harding, R.M.; Vu, M.; Dale, J.L. Identification and sequence analysis of potyviruses infecting crops in Vietnam. *Arch. Virol.* **2008**, *153*, 45-60. pmid:17906829. doi:10.1007/s00705-007-1067-1.<br>Fajardo, T.V.; Nishijima, M.; Buso, J.A.; Torres, A.C.; Ávila, A.C.; Resende, R.O. Garlic viral complex: Identification of potyviruses and carlavirus in central Brazil. *Fitopatologia Brasileira* **2001**, *26*, 619-626, doi: 10.1590/S0100-41582001000300007.<br>van Dijk, P. Survey and characterization of potyviruses and their strains of Allium species. *Neth. J. Plant Pathol.* **1993**, *99*, 1-48, doi: 10.1007/BF02017734.<br>Salomon, R.; Koch, M.; Levy, S.; Gal-On, A.; Marshall, G. Detection and identification of the viruses forming mixed infection in garlic. In Proceedings Diagnostics in Crop Production, British Crop Protection Council, Farnham, UK, 1996; no. 65, pp. 193-198.<br>Dovas, C.I.; Hatziloukas, E.; Salomon, R.; Barg, E.; Shiboleth, Y.; Katis, N.I. Incidence of viruses infecting Allium spp. in Greece. *Eur. J. Plant Pathol.* **2001**, *107*, 677-684. doi:10.1023/A:1011958914573.<br>Mohammad, G.; Kawas, H.; Al-Safadi, B. Survey of garlic viruses in southern Syria. Damascus University. *J. Agri. Sci.* **2007**, *23*, 255-265,	Abd El-Wahab, A.S. Aphid-transmission efficiency of two main viruses on garlic in Egypt, onion yellow dwarf virus (OYDV-G) and leek yellow stripe virus (LYSV-G). *Acad. J. Entomol.* **2009**, *2*, 40-42<br>CABI/EPPO. Leek yellow stripe virus. Distribution Maps of Plant Diseases. Wallingford, CAB International, 2007, 999.

			Sward, R.J. Leek yellow stripe virus recorded in leek in Australia. *Australasian Plant Pathol*. **1991**, *20*, 14-15, doi: 10.1071/APP9910014.		
Lettuce Italian necrotic virus	KP769852	Italy, France	Ciuffo, M.; Mammella, M.; Vallino, M.; Caciagli, P.; Turina, M. Molecular identification and biological characterization of a new potyvirus in lettuce. *Arch. Virol.* **2016**, *161*, 2549–54, doi: 10.1007/s00705-016-2920-x. Desbiez, C.; Schoeny, A.; Maisonneuve, B.; Berthier, K.; Bornard, I.; Chandeysson, C.; Fabre, F.; Girardot, P.G.; Lecoq, H.; Lot, H. et al. Molecular and biological characterization of two potyviruses infecting lettuce in southeastern France. *Plant Pathol.* **2017**, *66*, 970–979, doi: 10.1111/ppa.12651.	*Myzus persicae*	Ciuffo, M.; Mammella, M.; Vallino, M.; Caciagli, P.; Turina, M. Molecular identification and biological characterization of a new potyvirus in lettuce. *Arch. Virol.* **2016**, *161*, 2549–54, doi: 10.1007/s00705-016-2920-x.
Lettuce mosaic virus	X97705	Egypt, Ghana, Kenya, Malawi, Mauritius, Morocco, Sierra Leone, South Africa, Tanzania, Tunisia, Zambia, Zimbabwe, China, India, Iran, Iraq, Israel, Japan, Jordan, Lebanon, Malaysia, Saudi Arabia, Syria, Taiwan, Turkey, Yemen, Europe, Austria, Belgium, Croatia, Denmark, Finland, France, Germany, Greece, Hungary, Ireland, Italy, Netherlands, Norway, Poland, Portugal, Romania, Slovakia, Spain, Sweden, Switzerland, United Kingdom, Canada, Jamaica, Mexico, Trinidad and Tobago, United States, Australia New Zealand , Argentina, Brazil, Chile, Ecuador, Uruguay	Cabi Datasheet on Lettuce mosaic virus (lettuce mosaic). Available online: https://www.cabi.org/ISC/datasheet/30269 (Accessed on 2 April 2020).	*Myzus persicae, Myzus euphorbiae, Myzus persicae, Aphis gossypii, Myzus euphorbiae, Aphis fabae, Hyperomyzus lactucae, Macrosiphum barri*	Broadbent, L.; Tinsley, T.W.; Buddin, W.; Roberts, E.T. The spread of lettuce mosaic in the field. *Ann. Appl. Biol.* **1951**, *38*, 689–706 Moreno, A.; Fereres, A. Virus diseases in lettuce in the Mediterranean Basin. *Adv. Virus Res.* **2012**, *84*, 249-278, doi: 10.1016/B978-0-12-394314-9.00007-5. Dinant, S.; Lot, H. Lettuce mosaic virus. *Plant Pathol*. **1992**, *41*, 528-542, doi: 10.1111/j.1365-3059.1992.tb02451.x.
Lily mottle virus	AJ564636	India, Italy, Netherlands, USA, Japan, Israel,	Aravintharaj, R.; Balaji, C.G.; Nagendran, K.; Priyanka, R.; Karthikeyan, G. First report of lily mottle virus on lily (*Lilium sp.)* in southern India. *VirusDis*. **2017**, *28*, 222–223, doi: 10.1007/s13337-017-0381-9.	*Aphis gossypii, Myzus persicae, Macrosiphum solanifolii*	Mowat, W.P.; Stefanac, Z. Aphid-transmitted viruses from lilies in Britain. *Ann. Appl. Biol.* **1974**, *76*, 281-287.

		Lithuania, China, Italy, Argentina.;	Rizzo, D.; Stefani, L.; Paoli, M.; Lazzereschi, S.; Nesi, B.; Pecchioli, S.; Della Bartola, M.; Materazzi, A.; Grassotti, A. Occurrence of lily mottle virus on Lilium in Italy. *Plant Dis.* **2012**, *96*, 771, doi: 10.1094/PDIS-12-11-1019-PDN.<br>Asjes, C.J. Control of aphid-borne Lily symptomless virus and lily mottle virus in Lilium in the Netherlands. *Virus Res.* **2000**, *71*, 23–32, doi: 10.1016/s0168-1702(00)00185-4.<br>Fisher, J.R.; Rutherford, S.C.; Fleming, N.K. Identification of lily mottle virus and lily symptomless virus co-infecting Asiatic lily in Ohio. *Plant Health Prog.* **2013**, doi:10.1094/PHP-2013-1017-01-BR.<br>Maeda, T.; Inoue, N.; Mitsuhata, K. A distinctive strain of tulip breaking virus isolated from lilies in Japan. *Nogaku Kenkyu* **1984**, *60*, 135–146.<br>Cohen, J.; Gera, A.; Loebenstein, G. Virus diseases of lilies in Israel. *Acta Hortic.* **1996,** *432*, 84–87, doi: 10.17660/ActaHortic.1996.432.9.<br>Navalinskiene, M.; Samuitiene, M. Viruses affecting some bulb and corm flower crops. *Biologija* **2001**, *4*, 40–42<br>Wang, R.; Wang, G.; Zhao, Q.; Zhu, Y.; Zhan, J.; Xie, Z.; An, L.; Wang, Y. Molecular and cytopathologic evidences for a mixed infection of multiple viruses on Lanzhou lily (*Lilium davidii var. unicolor*) in Northwestern China. *J. Plant Dis. Protect.* **2010**, *117*, 145-149, doi: 10.1007/BF03356351.<br>Rizzo, D.; Stefani, L.; Paoli, M.; Lazzereschi, S.; Nesi, B.; Pecchioli, S.; Della Bartola, M.; Materazzi, A.; Grassotti, A. Occurrence of lily mottle virus on Lilium in Italy. *Plant Dis.* **2012**, *96*, 771, doi: 10.1094/PDIS-12-11-1019-PDN.<br>Chinestra, S. C.; Facchinetti, C.; Curvetto, N.R.; Marinangeli, P.A. Detection and frequency of lily viruses in Argentina. *Plant Dis.* **2010**, *94*, 1188-1194, doi: 10.1094/PDIS-07-09-0419.		
Lupinus mosaic virus	EU847625	Czech Republic	Sarkisova, T.; Petrzik, K. Determination of the complete nucleotide sequence of a lupine potyvirus isolate from the Czech Republic reveals that it belongs to a new member of the genus *Potyvirus*. *Arch Virol.* **2011**, *156*, 167–169, doi: 10.1007/s00705-010-0843-5.	Unknown	
Lycoris mild mottle virus	AF399672	Taiwan	Chang, C.A.; Chen, C.C.; Hsu, H.T. Partial characterization of two potyviruses associated with golden spider lily severe mosaic disease. *Acta Hort.* **2002**, *568*, 127-134, doi: 10.17660/ActaHortic.2002.568.18.	Unknown	
Maize dwarf mosaic virus	AJ001691	Burkina Faso, Cameroon, Côte d'Ivoire, Egypt, Ethiopia, Kenya, Mauritius, Morocco, Niger, Nigeria, South Africa, Zambia, Zimbabwe, China, Georgia, India, Iran,	Cabi Datasheet on Maize dwarf mosaic virus (dwarf mosaic of maize). Available online: https://www.cabi.org/isc/datasheet/8157 (Accessed on 30 March 2020).	*Acyrthosiphon pisum*, *Aphis craccivora*, *Aphis decepta*, *Aphis fabae*, *Aphis gossypii*, *Aphis maidiradicis*, *Aphis spiraecola*, *Brevicoryne brassicae*, *Hysteroneura setariae*, *Macrosiphum euphorbiae*,	Ford, R.E.; Tosic, M.; Shukla, D.D. *Maize dwarf mosaic virus.* AAB Descriptions of Plant Viruses, Association of Applied Biology, *No. 341*, Wellesbourne, UK, 1989; pp. 5<br>Mijavec, A. Study of resistance of *Sorghum bicolor* (L.) Moench genotypes to maize dwarf mosaic virus. *Zbornik Radova Poljoprivrednog Fakulteta, Universitet u Beogradu.* **1991**, *36*, 9-37.

		Iraq, Israel, Kazakhstan, Pakistan, Philippines, South Korea, Taiwan, Turkey, Uzbekistan, Yemen, Bosnia, Herzegovina, Bulgaria, Croatia, Czechia, Czechoslovakia, Federal Republic of Yugoslavia, France, Germany, Greece, Hungary, Italy, Poland, Romania, Russia, Serbia and Montenegro, Spain, Ukraine, Canada, Cuba, Haiti, Mexico, United States, Argentina, Brazil, Chile, Colombia, Peru Venezuela, Australia,		*Metopolophium festucae, Myzus persicae, Rhopalomyzus poae, Rhopalosiphum maidis, Rhopalosiphum padi, Schizaphis graminum, Sitobion avenae, Therioaphis trifolii, Uroleucon ambrosiae*	
Malva vein clearing virus	FM212972	Germany, Iran, Hungary, Yugoslavia USA Brazil, Italy, Spain	Menzel, W.; Winter, S.; Richert-Pöggeler, K.R. First report of Malva vein clearing virus naturally occurring in hollyhock in Germany. *Plant Dis.* **2010**, *94*, 276, doi: 10.1094/PDIS-94-2-0276B. Alouzi, H.; Golnaraghi, A.; Rakhshandehroo, F. Natural occurrence of Malva vein clearing virus in Malva in Iran. *New Dis. Reports* **2017**, *35*, 15. doi: 10.5197/j.2044-0588.2017.035.015. Horváth, J.; Mamula, D.; Besada, W.H.; Juretic, N. Some properties of Malva vein vlearing virus isolated in Hungary and Yugoslavia. *Phytopath. Z.* **1979**, *95*, 51-58, doi: 10.1111/j.1439-0434.1979.tb01577.x. Costa, A.S.; Duffus, J.E. Occurrence of Malva yellow vein mosaic m California. *Plant Dis. Reptr.* **1957**, *41*, 1006-1008. Majorana, G.; Silberschmidt, K.S. Identificazione dell'agente eziologlco di una malattia della malva presente in Italia. *Ann. Fac. Agric. Univ. Bari*. **1967**, *21*, 5-14. Marco, S. Occurrence of alfalfa mosaic virus and Malva vein clearing virus on weed members of *Malvaceae* in Israel. *Plant Dis. Reptr.* **1975**, *59*, 34-36. Lunello, P.; Tourino, A.; Nunez, Y.; Ponz, F.; Sanchez, F. Genomic heterogeneity and host recovery of isolates of Malva vein clearing virus. *Virus Res.* **2009**, *140*, 91-97, doi: 10.1016/j.virusres.2008.11.006.	*Myzus persicae*	Horváth, J.; Mamula, D.; Besada, W.H.; Juretic, N. Some properties of Malva vein vlearing virus isolated in Hungary and Yugoslavia. *Phytopath. Z.* **1979**, *95*, 51-58, doi: 10.1111/j.1439-0434.1979.tb01577.x.
Meadow saffron breaking virus	AY388995	France	Poutaraud, A.; Desbiez, C.; Lemaire, O.; Lecoq, H.; Herrrbach, E.. characterization of a new potyvirus species infecting meadow saffron *Colchicum autumnale*. *Arch Virol.* **2004**, *149*, 1267-1277, doi: 10.1007/s00705-004-0307-x.	Unknown	

Mediterranean ruda virus	MF953305	Spain	Rodríguez-Nevado, C.; Montes, N.; Pagán, I. Ecological factors affecting the infection risk and population genetic diversity of a novel potyvirus in its native wild ecosystem. *Front. Plant Sci.* **2017,** *8*,1958, doi: 10.3389/fpls.2017.01958.	Unknown	
Moroccan watermelon mosaic virus	EF579955	Morocco, the Canary Islands, Italy, Spain, France, Tunisia, Zimbabwe, Niger, Cameroon, Sudan, South Africa, Greece.	Fischer, H.U.; Lockhart, B.E.L. Serious losses in cucurbits caused by watermelon mosaic virus in Morocco. *Plant Dis. Rep.* **1974**, *58*, 143-146.<br>Lecoq, H.; Justafre, I.; Wipf-Scheibel, C.; Desbiez, C. Moroccan watermelon mosaic virus newly reported on zucchini squash in France. *Plant Pathol.* **2008**, *57*, 766, doi: 10.1111/j.1365-3059.2008.01848.x.<br>Lecoq, H.; Desbiez, C. Viruses of cucurbit crops in the Mediterranean region: An ever-changing picture. *Adv. Virus Res.* **2012**, *84*, 67-126, doi: 10.1016/B978-0-12-394314-9.00003-8.<br>Lecoq, H.; Dafalla, G.; Desbiez, C.; Wipf-Scheibel, C.; Delecolle, B.; Lanina, T.; Ullah, Z.; Grumet, R. Biological and molecular characterization of Moroccan watermelon mosaic virus and a potyvirus isolate from Eastern Sudan. *Plant Dis.* **2001**, *85*, 547-552, doi: 10.1094/PDIS.2001.85.5.547.<br>Van der Meer, F.W.; Garnett, H.M. Purification and identification of a South African isolate of watermelon mosaic virus-Morocco. *J. Phytopathol.* **1987**, *120*, 255-270, doi: 10.1111/j.1439-0434.1987.tb04440.x.<br>Malandraki, I.; Vassilakos, N.; Xanthis, C.; Kontosfiris, G.; Katis, N.I.; Varveri, C. First report of Moroccan watermelon mosaic virus in zucchini crops in Greece. *Plant Dis.* **2014**, *98*, 702, doi: 10.1094/PDIS-10-13-1100-PDN.<br>Owolabi, A.T.; Rabenstein, F.; Ehrig, F.; Edgar, M.M.; Vetten, H.J. Strains of Moroccan watermelon mosaic virus isolated from *Lagenaria breviflorus* and *Coccinia barteri* in Calabar, Southeastern Nigeria. *Int. J. Virol.* **2012**, *8*, 258-270, doi: 10.3923/ijv.2012.258.270.<br>Roggero, P.; Dellavalle, G.; Lisa, V.; Stravato, V.M. First report of Moroccan watermelon mosaic potyvirus in zucchini in Italy. *Plant Dis.* **1998**, *82*, 351, doi: 10.1094/PDIS.1998.82.3.351B.	*Aphis spiraecola, Myzus persicae, Aphis fabae, Aphis gossypii, Aphis neriiy, Aphis spiraecolay, Brevicoryne brassicae, Myzocallis castanicola, Myzus persicae, Macrosiphum rosae, Macrosiphoniella sanborni, Uroleucon sonchi*	Owolabi, A.T.; Ekpiken, E.E. Transmission efficiency of two strains of Moroccan watermelon mosaic virus by two clones of *Aphis spiraecola* (Patch). *Int. J. Virol.* **2014**, *10*, 253-262<br>Owolabi, A.T.; Rabenstein, F.; Ehrig, F.; Edgar, M.M.; Vetten, H.J. Strains of Moroccan watermelon mosaic virus isolated from *Lagenaria breviflorus* and *Coccinia barteri* in Calabar, Southeastern Nigeria. *Int. J. Virol.* **2012**, *8*, 258-270<br>Chatzivassiliou, E.K.; Papapanagiotou, A.P.; Mpenardis, P.D.; Perdikis, D.C.; Menexes, G. Transmission of Moroccan watermelon mosaic virus (MWMV) by aphids in Greece. *Plant Dis.* **2016**, *100*, 601-606
Narcissus degeneration virus	AM182028	New Zealand, Australia, China, UK	Ward, L.I.; Veerakone, S.; Tang, J.; Clover, G.R.G. First report of narcissus degeneration virus, narcissus late season yellows virus, and narcissus symptomless virus on Narcissus in New Zealand. *Plant Dis.* **2009**, *93*, 964, doi: 10.1094/PDIS-93-9-0964A.<br>Wylie, S.J.; Jones, M.G.K. Complete genome sequences of seven carlavirus and potyvirus isolates from *Narcissus* and *Hippeastrum* plants in Australia, and proposals to clarify their naming. *Arch Virol.* **2012,** *157*, 1471-1480, doi: 10.1007/s00705-012-1319-6.<br>Chen, J.; Shi, Y.; Adams, M.; Zheng, H.Y.; Qin, B.X.; Chen, J.P. Characterisation of an isolate of Narcissus degeneration virus from Chinese narcissus (*Narcissus tazetta var. chinensis*). *Arch Virol.* **2007**, 152, 441-448, doi: 10.1007/s00705-006-0841-9.<br>Brunt A.A. A review of problems and progress in research on viruses and virus diseases of narcissus in Britain. *Acta Hortic.* **1980**, *110*, 23-30, doi: 10.17660/ActaHortic.1980.110.1.	*Myzus persicae*	Brunt A.A. A review of problems and progress in research on viruses and virus diseases of narcissus in Britain. *Acta Hortic.* **1980**, *110*, 23-30, doi: 10.17660/ActaHortic.1980.110.1

Narcissus late season yellows virus	KC691259	New Zealand, UK, Netherlands, Australia, China	Ward, L.I.; Veerakone, S.; Tang, J.; Clover, G.R.G. First report of Narcissus degeneration virus, Narcissus late season yellows virus, and narcissus symptomless virus on Narcissus in New Zealand. *Plant Dis*. **2009**, *93*, 964, doi: 10.1094/PDIS-93-9-0964A. Mowat, W.P.; Duncan, G.H.; Dawson, S. Narcissus late season yellows potyvirus: symptoms, properties and serological detection. *Ann. Appl. Biol*. **1988**, *113*, 531-544, doi: 10.1111/j.1744-7348.1988.tb03330.x. Brunt A.A. Narcissus. In *Viruses and virus-like diseases of bulbs and flower crops 1st ed*.; Loebenstein, G., Lawson, R.H., Brunt A.A. Eds.; Wiley: Chichester, UK, 1995; pp. 322–334, ISBN: 978-0-4719-5293-0. Chen, J.; Chen, J.P.; Langeveld, S.A.; Derks, A.F.L.M.; Adams, M.J. Molecular characterization of carla- and potyviruses from narcissus in China. *J. Phytopathol.* **2003**, *151*, 1-4, doi: 10.1046/j.1439-0434.2003.00674.x. Wylie, S.J.; Nouri, S.; Coutts, B.A.; Jones, M.G.K. Narcissus late season yellows virus and vallota speciosa virus found infecting domestic and wild populations of Narcissus species in Australia. *Arch Virol.* **2010**, *155*, 1171-1174, doi: 10.1007/s00705-010-0682-4.	*Myzus persicae*	Mowat, W.P.; Duncan, G.H.; Dawson, S. Narcissus late season yellows potyvirus: symptoms, properties and serological detection. *Ann. Appl. Biol*. **1988**, *113*, 531-544, doi: 10.1111/j.1744-7348.1988.tb03330.x.
Narcissus yellow stripe virus	AM158908	UK, USA, Poland, Japan, Australia, China	Brunt, B.A.A. Narcissus mosaic virus. *Ann. Appl.Biol.* **1966**, *58*, 13-23. Bampi, D.; Reinsel, M.D.; Hammond, J. Identification of narcissus yellow stripe virus and a closely-related potyvirus isolate in plants of *Allium carinatum*. *Acta Hortic.* **2018**, *1193*, 39-46, doi: 10.17660/ActaHortic.2018.1193.6. Sochacki, D.; Kucharska, D.; Orlikowska, T. The occurrence of potyviruses narcissus yellow stripe virus (NYSV) and narcissus late season yellows virus (NLSYV) in narcissus crop in Poland. *Phytopathologia Polonica* **2003**, *28*, 75-79, doi: 10.1515/johr-2016-0016. Ohshima, K.; Mitoma, S.; Gibbs A. The genetic diversity of narcissus viruses related to turnip mosaic virus blur arbitrary boundaries used to discriminate potyvirus species. *PLoS ONE* **2018**, *13*, e0190511, doi: 10.1371/journal.pone.0190511. Wylie, S.J; Li, H.; Sivasithamparam, K.; Jones, M.G.K. Complete genome analysis of three isolates of narcissus late season yellows virus and two of narcissus yellow stripe virus: three species or one. *Arch. Virol.* **2014,** *159*, 1521-1525, doi: 10.1007/s00705-013-1969-z. Chen, J.; Lu, Y.W.; Shi, Y.H.; Adams, M.J.; Chen, J.P. Complete nucleotide sequence of the genomic RNA of narcissus yellow stripe virus from Chinese narcissus in Zhangzhou city, China. *Arch Virol.* **2006**, *151*,1673-1677, doi: 10.1007/s00705-006-0788-x.	*Acyrthosiphon solani, Aphis fabae, Macrosiphum euphorbiae, Acyrthosiphon pisum, Dysaphis plantaginea, Macrosiphum rosea, Myzus cerasi, Neomyzus circumflexus, Rhopalosiphoninus staphyleae*	Blanton, F.S.; Haasis, F.A. Insect transmission of the virus causing narcissus mosaic. *J. agric. Res.* **1942**, *65*, 13-9 Brunt, A.A. *Virus diseases of narcissus*. Glasshouse Crops Research Institute, Littlehanipton, Sussex, UK, 1971; pp. 18-37. Blanton, F.S., Haasis, F.A. Transmission of the narcissus mosaic virus by Aphids. *Jour. Econ. Ent.* **1939**, *32*, 469. Blanton, F. S.; Haasis, F.A. Three additional species of aphids transmitting narcissus mosaic. (Scientific Note) *Jour. Econ. Ent.* **1940**, *33*, 942.
Nerine yellow stripe virus	EF362621	India, USA, Netherlands	Kumar, S.; Raj, R.; Kaur, C.; Raj, S.K. Association of nerine yellow stripe virus with mosaic disease of *Crinum asiaticum* ornamental plant in India. *Dis. notes* **2015,** *99*, 1655-1655, doi: 10.1094/PDIS-02-15-0211-PDN. Guaragna, M.A.; Lamborn, J.; Groth-Helms, D.; Juszczak, S.; Mollov, D.; Lockhart, B.; van Schadewijk, T.; Hammond, J.; Jordan, R. First report of nerine yellow	Unknown	

			stripe virus in Amaryllis in the United States. *Plant Dis.* **2013**, *97*, 1389-1389, doi: 10.1094/PDIS-11-12-1042-PDN.<br>Pham, K.T.K.; de Kock, M.J.D.; Lemmers, M.E.C.; Derks, A.F.L.M. Molecular identification of potyviruses infecting bulbous ornamentals by the analysis of coat protein (CP) sequences. *Acta Hortic.* **2011**, *901*, 167-172, doi: 10.17660/ActaHortic.2011.901.21.		
Nothoscordum mosaic virus		Australia, USA	Pares, R.D.; Gillings, M.R. Two new records of diseases caused by potyviruses in Australia. *Australas. Plant Path.* **1990**, *19*, 36–37 doi: 10.1071/APP9900036.<br>McKinney, H.H. Mosaic diseases in Canary Islands, West Africa and Gibralter. *J. Agric. Res*. **1929**, *39*, 557–578.	Unknown	
Onion yellow dwarf virus	AJ510223	Netherlands, Turkey, Egypt, Poland, Greece, France, Croatia, Ukraine, Argentina, USA, Israel, Ecuador, Iran, Spain, Nigeria, Serbia, China, Egypt, Morocco, South Africa, Indonesia, Italy, Iraq, Japan, Taiwan, Thailand, Turkey, Vietnam, Austria, Czechia, Czechoslovakia, Denmark, Estonia, Finland, Germany, Hungary, Lithuania, Moldova, Poland, Romania, Serbia, Slovenia, Spain, Switzerland, Ukraine, United Kingdom, Canada Cuba, Mexico, United States, Australia, New Zealand, Brazil, Chile, Uruguay	Van Dijk, P. Survey and characterization of potyviruses and their strains of Allium species. *Neth. J. Plant Pathol.* **1993**, *99*, 1-48, doi: 10.1007/BF02017734.<br>Fidan, H.; Baloglu, S. First report of onion yellow dwarf virus and leek yellow stripe virus in Garlic in Turkey. *Plant Dis*. **2009**, *93*, 672, doi: 10.1094/PDIS-93-6-0672C.<br>Mahmoud, S.Y.M; Maaty, S.A.A.E.; El-Borollosy, A.M.; Abdel-Ghaffar, M.H. Identification of onion yellow dwarf potyvirus as one of the major viruses infecting garlic in Egypt. *Am. Eurasian J. Agric. Environ. Sci*. **2007**, *2*, 746-755, doi: 10.3923/ijv.2008.1.13.<br>Krystyna, W.; Ewa, S.; Wojciech, S. Prevalence of infections with onion yellow dwarf virus, leek yellow stripe virus and garlic common latent virus in plants from the genus Allium. *Acta Scientiarum Polonorum* **2014**, *13*, 123-133.<br>Dovas, C.I.; Hatziloukas, E.; Salomon, R.; Barg, E.; Shiboleth, Y.; Katis, N.I. Incidence of viruses infecting *Allium spp.* in Greece. *Eu. J. Plant Pathol*. **2001,** *107*, 677-684 doi: 10.1023/A:1011958914573.<br>Lot, H.; Chovelon, V.; Souche, S.; Delecolle, B. Effects of onion yellow dwarf and leek yellow stripe viruses on symptomatology and yield loss of three French garlic cultivars. *Plant Dis.* **1998,** *82*, 1381-1385 doi: 10.1094/PDIS.1998.82.12.1381.<br>Vončina, D.; Ćurić, K.; Fabek, S.; Toth, N. First report of onion yellow dwarf virus, leek yellow stripe virus and garlic common latent virus on garlic in Croatia. *Plant Dis.* **2016**, *100*, 656-656, doi: 10.1094/PDIS-08-15-0952-PDN.<br>Snihur, H.; Shevchenko, T.; Sherevera, K.; Budzanivska, I.; Shevchenko, O. First report of onion yellow dwarf virus in Ukraine. *J. Plant Pathol.* **2019,** *65*, 47-53, doi: 10.1007/s42161-019-00350-2.<br>Conci, V.C.; Nome, S.F.; Milne, R.G. Filamentous viruses of garlic in Argentina. *Plant Dis.* **1992**, *76*, 594-596, doi: 10.1094/PD-76-0594<br>Melhus, I.E.; Reddy, C.S.; Henderson, W.J.; Vestal, E. A new virus disease epidemic on onions. *Phytopathology* **1929**, *19*, 73–77.<br>Shiboleth, Y.M.; Gal-on, A.; Koch, M.; Rabinowitch, H.D.; Salomon, R. Molecular characterization of onion yellow dwarf virus (OYDV) infecting garlic (*Allium sativum* L.) in Israel: thermotherapy inhibits virus elimination by meristem tip culture. *Ann Appl Biol.* **2001**, *138*, 187-195, doi: 10.1111/j.17447348.2001.tb00101.x.	*Myzus persicae, Aphis craccivora, Aphis gossypii, Myzus ascalonicus*	Abd El-Wahab, A.S. Aphid-transmission efficiency of two main viruses on garlic in Egypt, onion yellow dwarf virus (OYDV-G) and leek yellow stripe virus (LYSV-G). *Acad. J. Entomol.* **2009**, 2, 40-42<br><br>Kumar, P.; Dhawan, P.; Mehra, R. Characterization, transmission and host range of onion yellow dwarf virus. *Plant Dis. Res.* **2011**, *26*, 176.<br>Szirmai, J. Virusveszely a makoi hagy-matermesztesben. *Novenytermeles* **1958**, *7*, 63.

			Sivaprasad, Y.; Garrido, P.; Mendez, K.; Pachacama, S.; Garrido, A.; Ramos, L. First report of onion yellow dwarf virus infecting onion in Ecuador. *J. Plant Pathol.* **2017**, *99*, 296, doi: 10.4454/jpp.v99i1.3820.<br>Saffar, Z.N.; Torabi, S.; Naghavi, M.; Golnaraghi, A.R.; Aryakia, E. Onion yellow dwarf virus on leek, onion, shallot and welsh onion in Iran. *Plant Pathol.* **2013**, *95*, 73, doi: 10.4454/JPP.V95I4.013.<br>Fernández-Tabanera, E.; Fraile, A.; Lunello, P.; García-Arenal, F.; Ayllón, M.A. First report of onion yellow dwarf virus in leek (*Allium ampeloprasum* var. porrum) in Spain. *Plant Dis.* **2018**, *102*, 256, doi: 10.1094/PDIS-06-17-0892-PDN.<br>Majumder, S.; Yadav, V.; Yakasai, M.A.; Muhammad, J.Y. First report of onion yellow dwarf virus in garlic from Nigeria. *J. Plant Pathol.* **2017**, *99*, 299, doi: 10.4454/jpp.v99i1.3821.<br>Milošević, D.; Gvozdanović-Varga, J.; Ignjatov, M.; Nikolić, Z.; Vučurović, I.; Vučurović, A.; Stanković, I. First report of onion yellow dwarf virus infecting shallot in Serbia. *Plant Dis.* **2015**, *99*, 1450, doi: 10.1094/PDIS-04-15-0370-PDN.<br>Su, Y.; Liu, L.X.; Liu, B.; Su, Y.; Wang,Y.Z.; Li, X.Y.; Zhang, C.Y. First report of onion yellow dwarf virus on shallot (*Allium cepa var. aggregatum*) in China. *Plant Dis.* **2019**, *103*, 778, doi: 10.1094/PDIS-08-18-1440-PDN.<br>Plantwise Knowledge Bank on onion yellow dwarf. Available online: https://www.plantwise.org/knowledgebank/datasheet/37485 (Accessed on 28 March 2020).		
Ornithogalum mosaic virus	JQ807997	Japan, South Korea, New Zealand, USA, Netherlands, France, South Africa, Israel, India, South Korea, Japan, New Zealand, Australia, China	Matsumoto, T.; Yamamoto, H.; Fuji, S.; Inoue, M. Characterization of a novel potyvirus tentatively named Ornithogalum virus 3, successfully isolated from *O. thyrsoides* co-infected with two other potyviruses by single-aphid inoculation. *J. Gen. Plant Pathol*. **2008**, *74*, 76-80, doi: 10.1007/s10327-007-0057-1.<br>Cho, S.Y.; Lim, S.; Kim, H.; Yi, S.I.; Moon, J.S. Complete genome sequence of ornithogalum mosaic virus infecting *Gladiolus spp.* in South Korea. *Genome Announc.* **2016**, *4*, e00816, doi: 10.1128/genomeA.00816-16.<br>Smith, F.F.; Brierley P. Ornithogalum mosaic. *Phytopathology* **1944**, *34*, 497-503.<br>Bouwen, I.; Von der Vlugt, R.A.A. Natural infection of *Alstroemeria caryophyllea* with ornithogalum mosaic virus. *Plant Dis.* **1989**, *84*, 202, doi: 10.1094/PDIS.2000.84.2.202C.<br>Grisoni, M.; Moles, M.; Farreyrol, K.; Rassaby, L.; Davis, R.; Pearson, M. Identification of potyviruses infecting vanilla by direct sequencing of a short RT-PCR amplicon. *Plant Pathol.* **2006**, *55*, 523-529, doi: 10.1111/j.1365-3059.2006.01397.x.<br>Burger, J.T.; Von Wechmar, M.B. Purification and some properties of South African isolates of ornithogalum mosaic virus. *Phytopathology* **1989**, *79*, 385-391, doi: 10.1094/Phyto-79-385.<br>Zeidan, M.; Cohen, J.; Watad, A.; Gera, A. Improved purification and molecular properties of omithogalum mosaic virus in Israel. *Ann. App. Biol.* **1998**, *133*, 167-176, doi: 10.1111/j.1744-7348.1998.tb05817.x.	*Myzus persicae*	Matsumoto, T.; Yamamoto, H.; Fuji, S. et al. Characterization of a novel potyvirus tentatively named Ornithogalum virus 3, successfully isolated from O. thyrsoides co-infected with two other potyviruses by single-aphid inoculation. *J. Gen. Plant Pathol.* **2008**, *74*, 76–80 https://doi.org/10.1007/s10327-007-0057-1

			Kaur, C.; Raj, S.K.; Snehi, S.K.; Goel, A.K.; Roy, R.K. Natural occurrence of ornithogalum mosaic virus newly reported on gladiolus in India. *New Dis. Rep.* **2011**, *24*, 2, doi: 10.5197/j.2044-0588.2011.024.002. Fuji, S.; Inoue, M.; Yamamoto, H.; Furuya, H.; Naito, H.; Matsumoto, T. Nucleotide sequences of the coat protein gene of potyviruses infecting *Ornithogalum thyrsoides*. *Arch. Virol.* **2003**, *148*, 613-621, doi: 10.1007/s00705-002-0961-9. Wei, T.; Pearson, M.N.; Cohen, D. First report of ornithogalum mosaic virus and Ornithogalum virus 2 in New Zealand. *New Dis. Rep.* **2006**, *55*, 820, doi: 10.1111/j.1365-3059.2006.01375.x. Wylie, S.J.; Li, H.; Dixon, K.W.; Richards, H.; Jones, M.G. Exotic and indigenous viruses infect wild populations and captive collections of temperate terrestrial orchids (*Diuris species*) in Australia. *Virus Res.* **2013,** *171*, 22-32, doi: 10.1016/j.virusres.2012.10.003. Chen, C.C.; Chang, Y.L.; Chang, C.A.; Tsai, H.T. Molecular evidences for the dual infection of ornithogalum mosaic virus and pterostylis virus Y in an imported *Ornithogalum thyrsoides* plant with severe mosaic symptom. *Plant Pathol. Bull.* **2009**, *9*, 184-185.		
Ornithogalum virus 2	AB271783	New Zealand, Japan	Wei, T.; Pearson, M.N.; Cohen, D. First report of ornithogalum mosaic virus and ornithogalum virus 2 in New Zealand. *New Dis. Rep.* **2006**, *55*, 820, doi: 10.1111/j.1365-3059.2006.01375.x. Matsumoto, T.; Yamamoto, H.; Fuji, S.; Inoue, M. Isolation and characterization of a novel potyvirus tentatively named ornithogalum virus 2. *J. Gen. Plant Pathol.* **2007**, *73*, 222–224, doi: 10.1007/s10327-007-0010-3.	*Myzus persicae*	Matsumoto, T.; Yamamoto, H.; Fuji, S.; Inoue, M. Isolation and characterization of a novel potyvirus tentatively named ornithogalum virus 2. *J. Gen. Plant Pathol.* **2007**, *73*, 222–224, doi: 10.1007/s10327-007-0010-3.
Ornithogalum virus 3	AB282754	Japan	Matsumoto, T.; Yamamoto, H.; Fuji, S.; Inoue, M. Isolation and characterization of a novel potyvirus tentatively named ornithogalum virus 2. *J. Gen. Plant Pathol.* **2007**, *73*, 222–224, doi: 10.1007/s10327-007-0010-3.	*Myzus persicae*	Matsumoto, T.; Yamamoto, H.; Fuji, S.; Inoue, M. Isolation and characterization of a novel potyvirus tentatively named ornithogalum virus 2. *J. Gen. Plant Pathol.* **2007**, *73*, 222–224, doi: 10.1007/s10327-007-0010-3.
Panax virus Y	GQ916624	China	Yan, Z.L.; Song, L.M.; Zhou, T.; Zhang, Y.J.; Li, M.F.; Li, H.F.; Fan, Z.F. Identification and molecular characterization of a new potyvirus from *Panax notoginseng*. *Arch Virol*. **2010**, *155*, 949–957, doi: 10.1007/s00705-010-0672-6.	Unknown	
Papaya leaf distortion mosaic virus	BD171712	China, Japan, Taiwan	Kawano, S.; Yonaha, T. The occurrence of papaya leaf- distortion mosaic virus in Okinawa. *Tech. Bull. FFTC* **1992**, *132*, 13-23. Tuo, D.C.; Shen, W.T.; Yan, P.; Li, C.Q.; Gao, L.; Li, X.Y.; Li, H.P.; Zhou, P. Complete genome sequence of an isolate of papaya leaf distortion mosaic virus from commercialized PRSV-resistant transgenic papaya in China. *Acta Virol.* **2013**, 57, 452-455, doi: 10.4149/av_2013_04_452. Bau, H.J.; Kung, Y.J.; Raja, J.A.; Chan, S.J.; Chen, K.C.; Chen, Y.K.; Wu, H.W.; Yeh, S.D. Potential threat of a new pathotype of papaya leaf distortion mosaic virus infecting transgenic papaya resistant to papaya ringspot virus. *Phytopathology* **2008**, *98*, 848-856, doi: 10.1094 / PHYTO-98-7-0848.	Unknown	

Papaya ringspot virus-P	X67673	Côte d'Ivoire, Egypt, Mauritius, Nigeria, Tanzania, Tunisia, Uganda, Bangladesh, China, India, Indonesia, Iran, Israel, Japan, Lebanon, Malaysia, Nepal, Pakistan, Philippines, Singapore, Sri Lanka, Syria, Taiwan, Thailand, Turkey, Vietnam, Yemen, Cyprus, Finland, France, Germany, Italy, Poland, Spain, Bahamas, British Virgin Islands, Costa Rica, Cuba, Dominican Republic, El Salvador, Guadeloupe, Honduras, Jamaica, Mexico, Puerto Rico, Saint Kitts and Nevis, Trinidad and Tobago, U.S. Virgin Islands, United States, Australia, French Polynesia, Papua New Guinea, Samoa, Solomon Islands, Tonga, Brazil Colombia, Ecuador, Venezuela	Cabi datasheet on Papaya ringspot virus. Available online: https://www.cabi.org/isc/datasheet/45962#REF-DDB--27 (Accessed online on 2 April 2020).	*Aphis craccivora, Aphis gossypii, Aphis spiraecola, Aulacorthum solani, Macrosiphum euphorbiae, Myzus persicae, Toxoptera citricida*	Purcifull, D.F.; Edwardson, J.R.; Hiebert, E.; Gonsalves, D. *Papaya ringspot virus.* CMI/AAB. Description of Plant Viruses, No. 292, Association of Applied Biologists: Wellesbourne, UK, **1984**.
Papaya ringspot virus-W	AY027810	Cuba, Brazil, USA, India, Sudan, Nepal, Brazil, Costa Rica, Honduras, Guadeloupe, Martinique, Mexico, Panama, Venezuela, Bangladesh, Australia, Thailand, East Timor,	Rodríguez-Martínez, D.; dos Reis, F.A.; Duarte, P.D.S.G.; Galvino-Costa, S.B.F.; Olmedo, J.G. First report and molecular characterization of an isolate of papaya ringspot virus (PRSV-W) detected in pumpkin in Cuba. *Biosci. J.* **2015**, 31, 1133-1142, doi: 10.14393/BJ-v31n4a2015-26181. Inoue-Nagata, A.K.C.; Franco, M.; Martin, D.P.; Rezende, J.A.M.; Ferreira, G.B.; Dutra, L.S.; Nagata, T. Genome analysis of a severe and a mild isolate of papaya ringspot virus-type W found in Brazil. *Virus Gen.* **2007,** *35*, 119-127, doi: 10.1007/s11262-006-0032-5.	*Aphis gossypii, Aphis craccivora, Myzus persicae, Semiaphis heracleid, Macrosiphum solidaginis, Cavariella salicicola, Toxoptera aurantia*	Kumar, N.K.K.; Singh, H.S.; Kalleshwaraswamy, C.M. Aphid (Aphididae: Homoptera) vectors of papaya ringspot virus (PRSV), bionomics, transmission efficiency and factors contributing to epidemiology. *Acta Hortic.* **2010**, *851*, 431-443, doi: 10.17660/ActaHortic.2010.851.67. Chao, C. H.; Chen, C. C. Transmission of papaya ringspot virus type-W by aphids.

	Taiwan, Israel, Iran, South Africa, Zimbabwe, Botswana, Nigeria, Chad, Ethiopia, Afghanistan, Turkmenistan, Pakistan, Egypt, Mali	Webb, R.E.; Scott, H.A. Isolation and identification of watermelon mosaic viruses 1 and 2. *Phytopathology* **1965**, *55*, 895–900.<br>Verma, R.; Baranwal, V.K.; Prakash, S.; Tomer, S.P.S.; Pant, R.P.; Ahlawat, Y.S. First Report of papaya ringspot virus W in sponge gourd from India. *Plant Dis.* **2007**, *90*, 974-974, doi: 10.1094/PD-90-0974B.<br>Mohammed, H.; Manglli, A.; Zicca, S.; El Hussein, A.; Mohamed, M.; Tomassoli, L. First report of papaya ringspot virus in pumpkin in Sudan. *New Dis. Rep.* **2012**, *26*, doi: 10.5197/j.2044-0588.2012.026.026.<br>Dahal, G.; Lecoq, H.; Albrechtsen, S.E. Occurrence of papaya ringspot potyvirus and cucurbit viruses in Nepal. *Ann. Appl. Biol.* **1997**, *130*, 491-502, doi: 10.1111/j.1744-7348.1997.tb07677.x.<br>Lastra, R. Occurrence of cucurbit viruses in Venezuela. *Plant Dis. Rep*. **1968,** *52*, 171-174.<br>Quiot, J.B.; Kaan, F.; Beramis, M. Identification d'une souche de la mosaïque de la pastèque (watermelon mosaic virus 1) aux Antilles françaises. *Annales de Phytopathologie* **1971**, *3*, 125-130.<br>Delgadillo-Sánchez, F.; Garzón-Tiznado, J.A.; Vega-Pina, A. Cucurbit viruses in Mexico: a survey. *Revista Mexicana de Fitopatología* **1989**, *7*, 136-139.<br>Rivera, C.; Ramírez, P.; Rodríguez, C.M. Papaya ringspot virus associated with a severe mosaic in melon. *Turrialba* **1991**, *41*, 437-443.<br>Espinoza, H.R.; McLeod, P.J. Use of row cover in cantaloupe (*Cucumis melo* L) to delay infection of aphid-transmitted viruses in Honduras. *Turrialba* **1994**, *44*, 179-183.<br>Yuki, V.A.; Rezende, J.A.M.; Kitajima, E.W.; Barroso, P.A.V.; Kuniyuki, H.; Groppo, G.A.; Pavan, M.A. Occurrence, distribution, and relative incidence of five viruses infecting cucurbits in the state of São Paulo, Brazil. *Plant Dis.* **2000**, *84*, 516-520, doi: 10.1094/PDIS.2000.84.5.516.<br>Herrera, J.A.; Osorio, J.M.; Salazar, L.C.; Fernández, O. Hospedantes de los Potyvirus del melón en la provincia de Los Santos, Panamá. *Boletín de Sanidad Vegetal-Plagas* **2006**, 32, 95-107.<br>Greber, R.S. Watermelon mosaic virus 1 and 2 in Queensland cucurbit crops. *Aust. J. Agric. Res.* **1978**, *29*, 1235-1245, doi: 10.1071/AR9781235.<br>Attasart, P.; Charoensilp, G.; Kerbundit, S.; Panyim, S.; Juricek, M. Nucleotide sequence of a Thai isolate of papaya ringspot virus type W. *Acta Virol*. **2002**, *l46*, 241-246.<br>Maina, S.; Coutts, B.A.; Edwards, O.R.; de Almeida, L.; Ximenes, A.; Jones, R.A.C. Papaya ringspot virus populations from East Timorese and Northern Australian cucurbit crops: biological and molecular properties, and absence of genetic connectivity. *Plant Dis*. **2017**, *101*, 985-993, doi: 10.1094/PDIS-10-16-1499-RE.<br>Wang, J.J.; Yeh, S.D. Analyses of the coat protein genes of papaya ringspot virus W type isolates from different areas of Taiwan. (Unpublished, Genbank Acc. no. AAK17011)	Bulletin of Taichung District Agricultural Improvement Station. **1991**, *31*, 55-61.

			Naeimifar, M.; Pourrahim, R.; Zadehdabagh, G. Natural infection of *Citrullus colocynthis* by papaya ringspot virus-W in Iran. *Plant Dis.* **2014**, *98*, 1748, doi: 10.1094/PDIS-06-14-0600-PDN.<br>Gonsalves, D.; Tripathi S.; Carr, J.B.; Suzuki, J.Y. Papaya ringspot virus. The plant health instructor. **2010** doi:10.1094/PHI-I-2010-1004-01.<br>Ali, A.; Natsuaki, T.; Okuda, S. Identification and molecular characterization of viruses infecting cucurbits in Pakistan. *J. Phytopathol.* **2004**, *152*, 677-682, doi: 10.1111/j.1439-0434.2004.00915.x.<br>Israel (Unpublished, Genbank Acc.no. JF737858)		
Paris mosaic necrosis virus	MF509898	China	Lan, P.; Zhao, J.; Zhou, Y.; Li, Y.; Shen, D.; Liaq, Q.; Li, R.; Li, F. Complete genome sequence of Paris mosaic necrosis virus, a distinct member of the genus Potyvirus. *Arch Virol.* **2018**, *163*, 787–790, doi: 10.1007/s00705-017-3649-x.	Unknown	
Parsnip mosaic virus		UK	Murant, A.F.; Munthe, T.; Goold, R.A. Parsnip mosaic virus, a new member of the potato virus Y group. *Ann. Appl. Biol.* **1970**, 65: 127–135, doi: 10.1111/j.1744-7348.1970.tb04570.x.	*Cavariella aegopodii, Cavariella theobaldi, Myzus persicae*	Murant, A.F.; Munthe, T.; Goold, R.A. Parsnip mosaic virus, a new member of the potato virus Y group. *Ann. Appl. Biol*. **1970**, *65*, 127–135
Passiflora chlorosis virus	DQ860147	France, Italy, Spain, USA, Uganda	Cardin, L.; Moury, B. First report of passiflora chlorosis virus in *Bituminaria bituminosa* in Europe. *Plant Dis*. **2009**, *93,* 196, doi: 10.1094/PDIS-93-2-0196A.<br>Baker, C.A.; Jones, L. A new Potyvirus found in *Passiflora incence* in Florida. *Plant Dis.* **2007**, *91*, 227.<br>Ochwo-Ssemakula, M.; Sengooba, T.; Adipala, E.; Edema, R.; Redinbaugh, M.G.; Aritua, V.; Winter, S. Characterization and distribution of a Potyvirus associated with passion fruit woodiness disease in Uganda. *Plant Dis*. **2012**, *96*, 659-665, doi: 10.1094/PDIS-03-11-0263.	Unknown	
Passion fruit woodiness virus	HQ122652	Nigeria, Argentina, Australia, Taiwan, Brazil, USA, Japan	Arogundade, O.; Oyekanmi, J.; Oresanya, A.; Ogunsanya, P.; Akinyemi, S.O.S.; Lava Kumar, P. First report of passion fruit woodiness virus associated with passion fruit woodiness disease of passion fruit in Nigeria. *Plant Dis.* **2018,** *102*, 1181-1181, doi: 10.1094/PDIS-10-17-1614-PDN.<br>Di Piero, R.M.; Rezende, J.A.M.; Yuki, V.A.; Pascholati, S.F.; Delfino, M.A . Transmissão do passion fruit woodiness virus por *Aphis gossypii* (Glover) (Hemiptera: Aphididae) colonização do maracujazeiro pelo vetor. *Neotrop. Entomol.* **2006**, *35*, 139-140, doi: 10.1590/S1519-566X2006000100019.<br>Greber, R.S. Passion-fruit woodiness virus as the cause of passion vine tip blight disease. *Queensland J. Agric. Anim. Sci.* **1966**, *23*, 533-538.<br>Chang, C.A. Characterization and comparison of passion fruit mottle virus, a newly recognized potyvirus, with passion fruit woodiness virus. *Phytopathology* **1991**, *82*, 1358-1363, doi: 10.1094/Phyto-82-1358.<br>Lima, J.A.A.; Oliveira, V.B.; Torres Filho, J. Avaliação dos graus de incidência de vírus em pomares de maracujazeiro, na Serra da Ibiapaba, Ceará. *Caatinga,* **1996**, *9*, 61-66.<br>Provvidenti, R. Passionfruit woodiness virus infectivity in *Phaseolus vulgaris* and sources of resistance. *Bean Improv. Coop. Ann. Rep.* **1992,** *35*, 144-145.	*Aphis gossypii, Myzus persicae*	Di Piero, R.M.; Rezende, J.A.M.; Yuki, V.A.; Pascholati, S.F.; Delfino, M.A. Transmission do passion fruit woodiness virus por *Aphis gossypiie* (Glover) (Hemiptera: Aphididae) colonização do maracujazeiro pelo vetor. *Neotrop Entomol.* **2006**, *35*, 139-140.<br>Chang, C.A. Characterization and comparison of passionfruit mottle virus, a newly recognized potyvirus, with passionfruit woodiness virus. *Phytopathology* **1991**, *82*, 1358-1363.

			Iwai, H.; Ohmori, T.; Kurokawa, Y.; Muta, T.; Arai, K. New report of passionfruit woodiness virus in Japan. *Ann. Phytopathol. Soci. Jpn*. **1996**, *62*, 459-465, doi: 10.3186/jjphytopath.62.459.		
Pea seed-borne mosaic virus	D10930	Algeria, Egypt, Ethiopia, Libya, Morocco, South Africa, Sudan, Tanzania, Tunisia, Zambia, Zimbabwe, Asia, India, Iran, Israel, Japan, Jordan, Lebanon, Nepal, Pakistan, Syria, Taiwan, Turkey, Yemen, Belgium, Bulgaria, Czechia, Czechoslovakia, Denmark, Finland, France, Germany, Netherlands, Poland, Romania, Russia, Serbia, Montenegro, Slovakia, Sweden, Switzerland, United Kingdom, Canada, United States, Australia, New Zealand, South America, Brazil, Peru	Cabi datasheet on Pea seed-borne mosaic virus. Available online: https://www.cabi.org/isc/datasheet/39776. (Accessed on 28 March 2020).	*Acyrthosiphon pisum*, *Aphis craccivora*, *Aphis fabae*, *Ovatus crataegaria*, *Rhopalosiphum padi*, *Uroleucon escalantii*	Cabi datasheet on pea seed-borne mosaic virus. Available online: https://www.cabi.org/isc/datasheet/39776
Peanut mottle virus	AF023848	USA, Burkina, Faso, Egypt, Kenya, Niger, Sudan, South Africa, Tanzania, Uganda and Zambia, India, Indonesia, Israel, Japan, Malaysia, Philippines, Taiwan, Australasia, Argentina, Colombia, Canada, USA, Caribbean, Cuba, China, South Korea, Israel, Iran, Cote d'Ivoire, Sudan	Demski, J.W.; Smith, D.H.; Kuhn, C.W. Incidence and distribution of peanut mottle virus in peanut in the United States. *Peanut Sci.* **1975**, *2*, 91-93, doi: 10.3146/i0095-3679-2-2-14. Plant Wise KnowledgeBank on Peanut mottle virus. Available online: https://www.plantwise.org/KnowledgeBank/datasheet/45569 (Accessed on 28 March 2020). An, M.N.; Li, R.; Gao, W.D.; Bi, X.Y.; Liang, Y.; Xia, Z.H.; Wu. Y.H. First Report of peanut mottle virus infecting peanut in Northeast China. *Plant Dis.* **2019**, *103*, 378, doi: 10.1094/PDIS-06-18-1037-PDN. Lim, S.; Lee, Y.H.; Igori, D.; Zhao, F.; Yoo, R.H.; Lee, S.H.; Baek, I.Y.; Moon, J.S. First report of peanut mottle virus infecting soybean in South Korea. *Plant Dis.* **2014,** *98*, 1285, doi: 10.1094/PDIS-04-14-0356-PDN. Spiegel, S.; Sobolev, I.; Dombrovsky, A.; Gera, A.; Raccah, B.; Tam, Y.; Beckelman, L.; Feigelson, V.; Holdengreber, Antignus, Y. Note: Characterization of a peanut	*Aphis craccivora*, *Rhopalosiphum padi*, *Myzus persicae*, *Aphis gossypii*, *Hyperomyzus lactucae*, *Aphis odinae*	Behncken, G.M. The occurrence of peanut mottle virus in Queensland. *Austrial. Jour. of Agric. Res.* **1970**, *21*, 465-472, doi: 10.1071/AR9700465. Bock, K.R. Peanut mottle virus in East Africa. *Ann. Appl. Biol.* **1973**, *74*, 171-179, doi:10.1111/j.1744-7348.1973.tb07736.x. Paguio, 0.R., Kuhn, C.W. Aphid transmission of peanut mottle virus. *Phytopathology* **1976**, *66*, 473-476, doi: 10.1094/Phyto-66-473.

			mottle virus isolate infecting peanut in Israel. *Phytoparasitica* **2008**, *36*, 168-174, doi: 10.1007/BF02981329.<br>Beikzadeh, N.; Hassani-Mehraban, A.; Peters, D. Molecular Identification of an isolate of peanut mottle virus (PeMoV) in Iran. *J. Agr. Sci. Tech.* **2015**, *17*, 765-776.<br>Samad, D.; Thowenel, J.C.; Dubern, J. Characterization of peanut mottle virus in Cote d'Ivoire. *J. Phytopathol*. **1993**, *139*, 10-16, doi: 10.1111/j.1439-0434.1993.tb01396.x.<br>Ahmed, A.H.; Idris, M.O. Peanut mottle virus in the Sudan. *Plant Dis.* **1981**, *65*, 692-693.		
Pecan mosaic-associated virus	KT633868	China	Su, X.; Fu, S.; Qian, Y.; Zhang, L.; Xu, Y.; Zhou, X. Discovery and small RNA profile of pecan mosaic-associated virus, a novel potyvirus of pecan trees. *Sci Rep.* **2016**, *6*, 26741, doi: 10.1038/srep26741.	Unknown	
Pennisetum mosaic virus	AY642590	China	Fan, Z.F.; Chen, H.Y.; Cai, S.S.; Deng, C.L.; Wang, W.J.; Liang, X.M.; Li, H.F. Molecular characterization of a distinct potyvirus from whitegrass in China. *Arch Virol.* **2003**, *148*,1219-1224, doi: 10.1007/s00705-003-0010-3.	*Myzus persicae, Macrosiphum avenae, Rhopalosiphum padi, Schizaphis graminum*	Deng, C.L.; Wang, W.J.; Wang, Z.Y.; Jiang, X.; Cao, Y.Y.; Zhou, T.; Wang, F.R.; Li, H.F.; Fan, Z.F. The genomic sequence and biological properties of pennisetum mosaic virus, a novel monocot-infecting potyvirus. *Arch. Virol.* **2008**, *153*, 921-927, doi: 10.1007/s00705-008-0068-z.
Pepper mottle virus	M96425	USA, Taiwan, Japan, Korea, Cuba, India.	Andrianifahanana, M.; Lovins, K.; Dute, R.; Sikora, E.J.; Murphy, J.F. Pathway for phloem-dependent movement of pepper mottle potyvirus in the stem of *Capsicum annuum*. *Phytopathology* **1997**, *87*, 892-898, doi: 10.1094/PHYTO.1997.87.9.892.<br>Cheng, Y.H.; Deng, T.C.; Chen, C.C.; Liao, J.Y.; Chang, C.A.; Chiang, C.H. First report of pepper mottle virus in bell pepper in Taiwan. *Plant Dis.* **2011**, *95*, 617, doi: 10.1094/PDIS-10-10-0721.<br>Ogawa, Y.; Hagiwara, K.; Iwai, H.; Izumi, S.; Arai, K. First report of pepper mottle virus on *Capsicum annuum* in Japan. *J. Gen. Plant Pathol.* **2003**, *69*, 348–350, doi: 10.1007/s10327-003-0059-6.<br>Han, J.H.; Choi, H.S.; Kim, D.H.; Lee, H.R.; Kim, B.D. Biological, physical and cytological properties of pepper mottle virus-SNU1 and its RT-PCR detection. *Plant Pathol. J.* **2006**, *22*, 155-160, doi: 10.5423/PPJ.2006.22.2.155.<br>Quiñones, M.; Arana, F.; Alfenas-Zerbini, P.; Soto, M.; Ribeiro, D.; Diaz, A.; González, D.; Carbonell, J.; Depestre, T.; Zerbini, F.M. First report of pepper mottle virus in sweet pepper in Cuba. *New Dis. Rep.* **2011**, *24*, 16, doi: 10.5197/j.2044-0588.2011.024.016.<br>Kaur, S.; Kang, S.S.; Sharma, A.; Sharma, S. First report of pepper mottle virus infecting chilli pepper in India. *New Dis. Rep.* **2014**, *30*, 14, doi:10.5197/j.2044-0588.2014.030.014.	*Myzus persicae*	Zitter, T.A. Transmission of pepper mottle virus from susceptible to resistent pepper cultivars. *Phytopathology* **1974**, *65*, 110-114.
Pepper severe mosaic virus	AM181350	Argentina, South Korea	Feldman, J.M.; Garcia, O. Pepper severe mosaic virus: a new potyvirus from pepper in Argentina. *Phytopathol. J.* **1977**, *73*, 115-122, doi: 10.1111/j.1439-0434.1977.tb02851.x.	Unknown	

			Ahn, H.I.; Yoon, J.Y.; Hong, J.S.; Yoon, H.I.; Kim, M.J.; Ha, J.H.; Rhie, M.J.; Choi, J.K.; Park, W.M.; Ryu, K.H. The complete genome sequence of pepper severe mosaic virus and comparison with other Potyviruses. *Arch. Virol.* **2006**, *151*, 2037-2045, doi: 10.1007/s00705-006-0776-1.		
Pepper veinal mottle virus	DQ645484	Afghanistan, India, Nigeria, Ghana, Kenya, Ivory coast, South Africa, Ethiopia, Burkina Faso, Senegal, Togo, China, Taiwan, Japan, Tunisia, Mali, Malaysia	Lal, S.B.; Singh, S. Identification of some virus diseases of vegetable crops in Afghanistan. *Plant Prot. Bull.* **1988**, 36, 83-89.<br>Nagaraju, R.; Reddy, H.R. Occurrence and distribution of bell pepper viruses around Bangalore. *Current research, University of Agricultural Sciences, Bangalore* **1980**, *155*, 6.<br>Alegbejo, M.D.; Uvah, I.I. Effect of intercropping pepper with tall companion crops on the incidence of pepper veinal mottle virus on pepper. *Nigerian J. Entomol.* **1987**, *7*, 82-87, doi: 10.3923/aj.2010.205.210.<br>Brunt, A.A.; Kenten, R.H.; Phillips, S. Symptomatologically distinct strains of pepper veinal mottle virus from four (4) West African solanaceous crops. *Ann App Biology*. **1978**, *88*, 115-119, doi: 10.1111/j.1744-7348.1978.tb00685.x.<br>Agranovsky, A.A. Virus diseases of pepper (*Capsicum annuum* L.) in Ethiopia. *J. Phytopathol*. **1993**, *138*, 89-97, doi: 10.1111/j.1439-0434.1993.tb01364.x.<br>Huguenot, C.; Furneaux, M.T.; Clare, J.; Hamilton, R.I. Sero-diagnosis of pepper veinal mottle virus in West Africa using specific monoclonal antibodies in DAS-ELISA. *J. Phytopathol.* **1996,** *144*, 29–32, doi:10.1111/j.1439-0434.1996.tb01484.x.<br>Zhang, S.B.; Zhao, Z.B.; Zheng, L.M.; Zhang, D.Y.; Liu, Y.; Liu, J.; Du, J.; Peng, J.; Yan, F.; Li, F.; Xie, Y.; Cheng, Z.B. First report of pepper veinal mottle virus infecting pepper in mainland China. *Plant Dis*. **2016**, *100*, 1025, doi: 10.1094/PDIS-08-15-0870-PDN.<br>Cheng, Y.H.; Wang, R.Y.; Chen, C.C.; Chang, C.A.; Jan, F.J. First report of pepper veinal mottle virus in tomato and pepper in Taiwan. *Plant Dis*. **2009**, *93*, 107, doi: 10.1094/PDIS-93-1-0107A.<br>De Wijs, J.J. Pepper veinal mottle virus in Ivory Coast. *Neth. J. Plant Pathol.* **1973**, *79*,189–193.<br>Matsumoto, K.; Yasaka, R.; Setoyama, T.; Kawano, S.; Ohshima, K. Chilli pepper rugose mosaic disease caused by pepper veinal mottle virus occurs on Ishigaki Island, Japan. *J. Gen. Plant Pathol.* **2016**, *82*, 57-60, doi: 10.1007/s10327-015-0634-7.<br>Gorsane, F.; Fakhfakh, H.; Tourneur, C.; Makni, M.; Marrakchi, M. Some biological and molecular properties of pepper veinal mottle virus isolates occurring in Tunisia. *Plant Mol. Biol. Report.* **1999**, *17*,149–158, doi: 10.1023/A:1007560222288.<br>Tsai, W.S.; Abdourhamane, I.K.; Kenyon, L. First report of pepper veinal mottle virus associated with mosaic and mottle diseases of tomato and pepper in Mali. *Plant Dis.* **2010**, *94*, 378, doi: 10.1094/PDIS-94-3-0378B.<br>Alegbejo, M.D.; Abo, M.E. Ecology, epidemiology and control of pepper veinal mottle virus (PVMV), Genus *Potyvirus*, in West Africa. *J. Sustain. Agr.* 2002, 20, 5-16, doi: 10.1300/J064v20n02_03.	*Myzus persicae, Aphis gossipii, Aphis craccivora, Aphis spiraecola, Aphis fabae, Hysteroneura setaria, Rhodalosiphum maidis, Toxopera citricidus*	Brunt, A.A.; Kenten, R.H.; Phillips, S. Symptomatologically distinct strains of pepper veinal mottle virus from four (4) West African solanaceous crops. *Ann App Biology.* **1978**, *88*, 115-119, doi: 10.1111/j.1744-7348.1978.tb00685.x<br>Fajinmi, A.A.; Odebode, C.A.; Fajinmi, O.B. The effect of agro-ecological zones on the incidence and distribution of aphid vectors of pepper veinal mottle virus on cultivated pepper (*Capsicum annuum* L.) in Nigeria. *Cent. Eur. Agric.* **2011**, *12*, 528-542, doi: 10.5513/JCEA01/12.3.951.

Pepper yellow mosaic virus	AB541985	Brazil	Inoue-Nagata, A.K.; Fonseca, M.E.; Resende, R.O.; Boiteux, L.S.; Monte, D.C.; Dusi, A.N.; de Avila, A.C.; an der Vlugt, A.A. Pepper yellow mosaic virus, a new potyvirus in sweetpepper, *Capsicum annuum*. *Arch. Virol.* **2002**, *147*, 849-55, doi: 10.1007/s007050200032.	Unknown	
Peru tomato mosaic virus	AJ437280	Ecuador, Peru	Insuasti, M.; Ochoa, J.; Martin, R.; Alvarez, R.; Quito, D. First report of potato virus V and Peru tomato mosaic virus on tamarillo (*Solanum betaceum*) orchards of Ecuador. *Plant Dis.* **2016**, *100*, 868, doi: 10.1094/PDIS-09-15-1063-PDN. Spetz, C.; Taboada, A.M.; Darwich, S.; Ramsell, J.; Salazar, L.F.; Valkonen, J.P. Molecular resolution of a complex of potyviruses infecting solanaceous crops at the centre of origin in Peru. *J. Gen. Virol.* **2003**, 84, 2565-2578, doi: 10.1099/vir.0.19208-0.	Unknown	
Pfaffia mosaic virus	AY485276	Brazil	Mota, L.D.C.; Della Vecchia, M.G.S.; Gioria, R.; Kitajima, E.W.; Rezende, J.A.M.; Camargo, L.E.A.; Amorim, L. Pfaffia mosaic virus: a new potyvirus found infecting *Pfaffia glomerata* in Brazil. *Plant Pathol.* **2004**, *53*, 368-373, doi: 10.1111/j.0032-0862.2004.01001.x.	*Aphis gossypii, Myzus persicae*	Mota, L.D.C.; Della Vecchia, M.G.S.; Gioria, R.; Kitajima, E.W.; Rezende, J.A.M.; Camargo, L.E.A.; Amorim, L. Pfaffia mosaic virus: a new potyvirus found infecting *Pfaffia glomerata* in Brazil. *Plant Pathol.* **2004**, *53*, 368-373, doi: 10.1111/j.0032-0862.2004.01001.x.
Pleione virus Y	AF185958	Australia	Gibbs, A.; Mackenzie, A.; Blanchfield, A.; Cross, P.; Wilson, C.; Kitajima, E.; Nightingale, M.; Clements, M. Viruses of orchids in Australia: their identification, biology and control. *Aust. Orchid Rev.* **2000**, *65*, 10-2.	Unknown	
Plum pox virus	AJ243957	Egypt, Tunisia, Azerbaijan, China, Georgia, India, Iran, Israel, Japan, Jordan, Kazakhstan, Lebanon, Pakistan, South Korea, Syria, Turkey, Albania, Austria, Belarus, Belgium, Bosnia and Herzegovina, Bulgaria, Croatia, Cyprus, Czechia, Denmark, Estonia, Finland, France, Germany, Greece, Hungary, Italy, Latvia, Lithuania, Luxembourg, Moldova, Montenegro, Netherlands, Norway, Poland, Portugal, Romania, Russia, Serbia, Slovakia,	Cabi datasheet on Plum pox virus. Available online: https://www.cabi.org/isc/datasheet/42203 (Accessed on 28 March 2020).	*Aphis craccivora, Aphis fabae, Aphis gossypii, Aphis hederae, Aphis spiraecola, Brachycaudus cardui, Brachycaudus helichrysi, Brachycaudus persicae, Hyalopterus pruni, Metopolophium dirhodum, Myzus persicae, Myzus varians, Phorodon humuli, Rhopalosiphum padi, Aphis arbuti, Dysaphis plantaginea, Sitobion fragariae, Uroleucon sonchi, Macrosiphum rosae, Dysaphis pyri,*	Glasa, M.; Candresse, T. Plum pox virus. In CMI/AAB Description of plant viruses no. 410; Jones A.T., Robinson, D.J., Boonham, N., Mumford, R., Eds.; Association of Applied Biologists: Wellesbourne, UK, 2005. Gildow, F.; Damsteegt, V.; Stone, A.; Schneider, W.; Luster, D.; Levy, L. Plum pox in North America: identification of aphid vectors and a potential role for fruit in virus spread. *Phytopathology* **2004**, *94*, 868-874, doi: 10.1094/PHYTO.2004.94.8.868. Levy, L.; Damsteegt, V.; Scorza, R.; Kolber, M. Plum Pox Potyvirus Disease of Stone Fruits. *APSnet Features* **2000,** doi: 10.1094/APSnetFeature-2000-0300.

		Slovenia, Spain, Sweden, Switzerland, Ukraine, United Kingdom, Canada, United States, New Zealand, Argentina Chile		*Megoura rosae,*	
Pokeweed mosaic virus	JX291161	Canada, USA	Klenov, A.; Hudak, K.A. Complete coding sequence and infectious clone of pokeweed mosaic virus Arkansas isolate. *Eur. J. Plant Pathol.* **2018**, *152*, 541–547, doi: 10.1007/s10658-018-1477-9. Di, R. Complete genome sequence of the pokeweed mosaic virus (PkMV)-New Jersey isolate and its comparison to PkMV-MD and PkMV-PA. *Genome Announc.* **2016**, *4*, e00929-16, doi: 10.1128/genomeA.00929-16.	*Myzus persicae*	Shepherd, R.J. Pokeweed mosaic virus. In CMI/AAB Description of plant viruses; Association of Applied Biologists: Wellesbourne, UK, 1972.
Potato virus A	AJ296311	Egypt, Tanzania, Tunisia, China, Pakistan, Taiwan, Turkey, Finland, France, Germany, Hungary, Poland, United States, New Zealand	Cabi datasheet on potato virus A. Available online: https://www.cabi.org/isc/datasheet/42524 (Accessed on 28 March 2020).	*Aulacorthum solani Aphis fabae, Acyrthosiphon pisum, Cavariella aegopodii*	Culjak, T.G.; Grubisic, D.; Kristic, I. Importance and control of aphids in potato production. (Vaznost i suzbijanje lisnih usi u proizvodnji krumpira.) *Glasilo Biljne Zastite* **2013,** *13*, 306-312.
Potato virus V	AJ243766	Peru, Uk, Netherlands, Finland, Scotland, Norway, Sweden, Ecuador	Fribourg, C.E.; Nakashima, J. Characterization of a new potyvirus from potato. *Phytopathology* **1984**, *74*, 1363-1369, doi: 10.1094/Phyto-74-1363. Rozendaal, A.; Van Binsbergen, J.; Anema, B.; Van Slogteren, D.H.M.; Bunt, M.H. Serology of a deviating potato virus YC strain in the potato variety Gladblaadje. *Potato Res.* **1971,** *14*, 24. Oruetxebarria, I.; Kekarainen, T.; Spetz, C.; Valkonen, J.P.T. Molecular characterization of potato virus V genomes from Europe indicates limited spatiotemporal strain differentiation. *Phytopathology*, **2000**, *90*, 437-444, doi:10.1094/PHYTO.2000.90.4.437. Oruetxebarria, I.; Valkonen, J.P.T. Analysis of the P1 gene sequences and the 3′-terminal sequences and secondary structures of the single-stranded RNA genome of potato virus V. *Virus Gen*. **2001**, *22*, 335–343, doi:10.1023/A:1011174509453. Shiel, P.J.; Miller, L.; Slack, S.A.; Berger, P.H. Isolation and partial nucleic acid characterization of a new isolate of potato virus V with distinct biological and serological properties. *Plant Dis.* **2004,** 88, 368-372, doi:10.1094/PDIS.2004.88.4.368. Oruetxebarria, I.; Guo, D.Y.; Merits, A.; Makinen, K.; Saarma, M.; Valkonen, J.P.T. Identification of the genome-linked protein in virions of potato virus A, with comparison to other members in genus Potyvirus. *Virus Res*. **2001,** *73*, 103–112. doi:10.1016/S0168-1702(00)00216-1. Insuasti, M.; Ochoa, J.; Martin, R.; Alvarez, R.; Quito, D. First Report of potato virus V and Peru tomato mosaic virus on Tamarillo (*Solanum betaceum*) orchards of Ecuador. *Plant Dis.* **2016**, *100*, 868, doi: 10.1094/PDIS-09-15-1063-PDN.	*Myzus persicae, Brachycaudus helichrysi, Macrosiphum euphorbiae, Rhopalosiphoninus latysiphon, Aphis fabae, Metopolophium dirhodum, Sitobion avenae, Acyrthosiphon pisum, Cavariella aegopodii*	Fribourg, C.E.; Nakashima, J. Characterization of a new potyvirus from potato. *Phytopathology* **1984**, *74*, 1363-1369, doi: 10.1094/Phyto-74-1363. Calvert, E.L.; Cooper, P.; McClure, J. An aphid transmitted strain of PVY-C recorded in potatoes in Northern Ireland. *Rec. Agric. Res.* **1980**, *28*, 63-74. Bell, A.C. The life-history of the leaf-curling plum aphid *Brachycaudus helichrysi* in Northern Ireland and its ability to transmit potato virus YC (AB). *Ann. appl. Biol.* **1983**, *102*, 1-6. Fox, A., Collins, L.E.; Macarthur, R.; Blackburn, L.F.; Northing, P. New aphid vectors and efficiency of transmission of potato virus A and strains of potato virus Y in the UK. *Plant Pathol.* **2017**, *66*, 325–335.

			Álvarez, N.; Jaramillo, H.; Gallo, G.; Gutiérrez, P.A.; Marín, M. Molecular characterization of potato virus Y (PVY) and potato virus V (PVV) isolates naturally infecting cape gooseberry (*Physalis peruviana*) in Antioquia, Colombia. *Agronomía Colombiana* **2018**, *36*, 13-23, doi: 10.15446/agron.colomb.v36n1.65051.		
Potato virus Y	U09509	Algeria, Benin, Egypt, Ethiopia, Libya, Mauritius, Morocco, Nigeria, South Africa, Sudan, Tanzania, Tunisia, Zambia, Zimbabwe, Afghanistan, Bangladesh, Bhutan, China, India, Indonesia, Iran, Iraq, Israel, Japan, Jordan, Kazakhstan, Lebanon, Nepal, Pakistan, Philippines, Saudi Arabia, South Korea, Syria, Taiwan, Tajikistan, Turkey, Vietnam, Yemen, Austria, Belarus, Belgium, Bosnia and Herzegovina, Bulgaria, Cyprus, Czechia, Denmark, Finland, France, Germany, Greece, Hungary, Ireland, Italy, Latvia, Lithuania, Montenegro, Netherlands, Poland, Portugal, Romania, Russia, Serbia, Serbia and Montenegro, Slovenia, Spain, Sweden, Switzerland, Ukraine, United Kingdom, Canada, Costa Rica, Cuba, Dominican	Cabi datasheet on Potato virus Y. Available online:https://www.cabi.org/ISC/datasheet/43762(Accessed online on 28 March 2020).	*Acyrthosiphon pisum, Aphis fabae, Aphis gossypii, Aphis nasturtii, Aphis spiraecola, Aulacorthum circumflexum, Aulacorthum solani, Lipaphis erysimi, Macrosiphum euphorbiae, Metopolophium festucae, Myzus cerasi, Myzus certus, Myzus ornatus, Myzus persicae, Phorodon humuli, Rhopalosiphoninus latysiphon, Rhopalosiphum insertum, Rhopalosiphum padi, Sitobion fragariae, Cavariella aegopodii, Aphis sambuci, Capitophorus elaeagni, Cryptomyzus galeopsidis, Hyperomyzus lactucae, Schizaphis graminum, Cryptomyzus ballotae, Diuraphis noxia, Hayhurtia atriplicis, Capitophorus hippophaes, Metopolophium dirhodum*	Kennedy J.S.; Day, M.F.; Eastop, V.F. *A Conspectus of Aphids as Vectors of Plant Viruses*.; Cab International: Wallingford, UK, 1962, doi: 10.4039/Ent951119-10. Culjak, T.G.; Grubisic, D.; Kristic, I. Importance and control of aphids in potato production. (Vaznost i suzbijanje lisnih usi u proizvodnji krumpira.) *Glasilo Biljne Zastite*, **2013**, *13*, 306-312. Blackman, R.L.; Eastop, V.F. *Aphids on the world's crops: an identification and information guide*, 2nd ed.; Wiley: London, UK, 2000; pp. 39 ISBN: 978-0-471-85191-2. Schröder, M.L.; Krüger, K. Preference of aphids (Hemiptera: Aphididae) for lucerne, maize, soybean and wheat and their potential as prospective border crops for potato virus Y management in seed potatoes. *Afr. Entomol*. 2014, 22, 144-155, doi: 10.4001/003.022.0131. Harrington, R.; Katis, N.; Gibson, R.W. Field assessment of the relative importance of different aphid species in the transmission of potato virus Y. *Potato Res.* **1986**, *29*, 67-75, doi: 10.1007/BF02361982. Piron, P.G.M. New aphid vectors of potato virus Y. *Neth. J. Plant Pathol.* **1986**, *92*, 223-229. Harrington, R.; Katis, N.; Gibson, R.W. Field assessment of the relative importance of different aphid species in the transmission of potato virus Y. *Potato Res.* **1986,** *29*, 67-76. Collar, J.L.; Fereres, A. Nonpersistent virus trans- mission efficiency determined by aphid probing behaviour during intracellular punctures. *Environ. Entomol.* **1998,** 27, 583-591. de Bokx, J.A., Pirone, P.G.M. Relative efficiency of a number of aphid species in the transmission of potato virus Y^N in The

		Republic El Salvador, Greenland, Guadeloupe, Jamaica, Mexico, Montserrat, Puerto Rico, United States, Australia, Cook Islands, New Zealand, Papua New Guinea, Samoa, Argentina, Brazil, Chile, Colombia, Peru, Venezuela			Netherlands. *Eur. J. Plant Pathol.* **1990,** *96*, 237-246. Fox, A.; Collins, L.E.R.; Macarthur, R.; Blackburn, L.F.; Northing, P. New aphid vectors and efficiency of transmission of potato virus A and strains of potato virus Y in the UK. *Plant Pathol.* **2017**, *66*, 325–335. Avila, A.L.; Vera, M.A.; Ortego, J.; Willink, E.; Ploper, L.D.; Conci, V.C. Aphid Species (Hemiptera: Aphididae) Reported for the First Time in Tucumán, Argentina. *Fla. Entomol.* 2014, *97*, doi: 10.1653/024.097.0402. Halbert, S.E.; Corsini, D.L.; Wiebe, M. A. *Potato virus Y* transmission efficiency for some common aphids in Idaho. *Am. J. Potato Res.* **2003**, *80*, 87-91, doi: 10.1007/BF02870207. Management of potato tuber necrotic viruses. Available online: Am. J. https://blogs.cornell.edu/potatovirus/pvy/aphid-vectors-of-pvy/Potato Res. 80:87–91 (Accessed on 9th June 2020)
Ranunculus leaf distortion virus	DQ152190	Italy, France	Turina, M.; Ciuffo, M.; Lenzi, R.; Rostagno, L.; Mela, L.; Derin, E.; Palmano, S. Characterization of four viral species belonging to the family *Potyviridae* isolated from *Ranunculus asiaticus*. *Phytopathology* **2006**, *96*, 560-566, doi: 10.1094/PHYTO-96-0560. Verdin, E.; Wipf-Scheibel, C.; Gognalons, P.; Aller, F.; Jacquemond, M.; Tepfer, M. Sequencing viral siRNAs to identify previously undescribed viruses and viroids in a panel of ornamental plant samples structured as a matrix of pools. *Virus Res.* **2017**, *241*, 19-28, doi: 10.1016/j.virusres.2017.05.019.	*Myzus persicae*	Turina, M.; Ciuffo, M.; Lenzi, R.; Rostagno, L.; Mela, L.; Derin, E.; Palmano, S. Characterization of four viral species belonging to the family Potyviridae isolated from *Ranunculus asiaticus*. *Phytopathology* **2006**, *96*, 560-566
Ranunculus mild mosaic virus	DQ152191	Japan, Italy, China	Hayahi, S.; Matsushita, Y.; Kanno, Y.; Kushima, Y.; Teramoto, S.; Takeshita, M. Field survey of ranunculus mild mosaic virus, tomato spotted wilt virus and cucumber mosaic virus infections in *Ranunculus asiaticus* L. in Japan by newly developed multiplex RT-PCR. *Eur. J. Plant Pathol.* **2018**, *150*, 205-12, doi: 10.1007/s10658-017-1268-8. Sacco, E.; Borghi, C.; Rabaglio M.; Lenzi, R.; Ciuffo, M.; Ruffoni, B.; Vaira, A.M. RT-PCR tests for sensitive detection of the major ranunculus-infecting viruses: field and in vitro applications. *Plant Pathol.* **2018,** *67*, 1967-1976, doi: 10.1111/ppa.12912. Wang, J. H.; Zhao, S.; Yang, X.M. First Report of ranunculus mild mosaic virus on *Ranunculus asiaticus* in Yunnan Province, China. *Plant Dis.* **2008**, *92*, 1585-1585, doi: 10.1094/PDIS-92-11-1585A.	*Myzus persicae*	Turina, M.; Ciuffo, M.; Lenzi, R.; Rostagno, L.; Mela, L.; Derin, E.; Palmano, S. Characterization of four viral species belonging to the family Potyviridae isolated from *Ranunculus asiaticus*. *Phytopathology* **2006**, *96*, 560-566

Ranunculus mosaic virus	DQ152192	Italy	Turina, M.; Ciuffo, M.; Lenzi, R.; Rostagno, L.; Mela, L.; Derin, E.; Palmano, S. Characterization of four viral species belonging to the family *Potyviridae* isolated from *Ranunculus asiaticus*. *Phytopathology* **2006**, *96*, 560-566, doi: 10.1094/PHYTO-96-0560.	*Myzus persicae*	Turina, M.; Ciuffo, M.; Lenzi, R.; Rostagno, L.; Mela, L.; Derin, E.; Palmano, S. Characterization of four viral species belonging to the family *Potyviridae* isolated from *Ranunculus asiaticus*. *Phytopathology* **2006**, *96*, 560-566, doi: 10.1094/PHYTO-96-0560.
Rhopalanthe virus Y	AF185956	Australia	Wilson, C.; Kitajima, E.; Nightingale, M.; Clements, M. Viruses of orchids in Australia; their identification, biology and control. *Aust. Orchid Rev.* **2000**, *65*, 10-21.		
Saffron latent virus	KY562565	Iran	Parizad, S.; Dizadji, A.; Koohi-Habibi, M.; Winter, S.; Kalantari, S.; Garcia-Arenal, F.; Ayllón, M.A. Prevalence of saffron latent virus (SaLV), a new potyvirus species, in saffron fields of Iran. *J. Plant Pathol.* **2017**, *99*, 802, doi: 10.4454/jpp.v99i3.3963.	Unknown	
Sarcochilus virus Y	AF185957	Australia	Wilson, C.; Kitajima, E.; Nightingale, M.; Clements, M. Viruses of orchids in Australia; their identification, biology and control. *Aust. Orchid Rev.* **2000**, *65*, 10–21	Unknown	
Scallion mosaic virus	AJ316084	Japan, China	Ohshima, K.; Muraoka, S.; Yasaka, R.; Adachi, S.; Tokuda, M. First report of scallion mosaic virus on wild Japanese garlic (*Allium macrostemon*) in Japan. *J. Gen. Plant Pathol.* **2016**, *82*, 61–64, doi: 10.1007/s10327-015-0636-5. Chen, J.; Zheng, H.; Chen, J.; Adams, M.J. Characterization of a potyvirus and a potexvirus from Chinese scallion. *Arch. Virol.* **2002**, *147*, 683–693, doi: 10.1007/s007050200018.		
Shallot yellow stripe virus	AJ865076	China, Indonesia, Thailand, Taiwan	Chen, J.; Wei, C.; Zheng, H.; Shi, Y.H.; Adams, M.J.; Lin, L.; Zhang, Q.Y.; Wang, S.J.; Chen, J.P. Characterisation of the welsh onion isolate of shallot yellow stripe virus from China. *Arch. Virol.* **2005,** 150, 2091-2099, doi: 10.1007/s00705-005-0580-3. van Dijk, P.; Sutarya, R. Virus diseases of shallot, garlic and welsh onion in Java, Indonesia and prospects for their control. *Onion Newsletter for the Tropics* **1992**, *4*, 57-61. van der Vlugt, R.A.A.; Steffens, P.; Cuperus, C.; Barg, E.; Lesemann, D.E.; Bos, L.; Vetten, H.J. Further evidence that shallot yellow stripe virus (SYSV) is a distinct potyvirus and reidentification of welsh onion yellow stripe virus as a SYSV strain. *Phytopathology* **1999,** *89*,148-155, doi: 10.1094/PHYTO.1999.89.2.148.		
Sorghum mosaic virus	AJ310197	China, USA, India, Philippines, Brazil, Nigeria	Zhang, Y.; Pennerman, K.; Wang, H.; Yin, G. Characterization of a sorghum mosaic virus (SrMV) isolate in China. *Saudi J. of Biol. Sci.* **2015**, 23, 237–242, doi:10.1016/j.sjbs.2015.02.013. Grisham, M.P.; Maroon-Lango, C.J.; Hale, A.L. First report of sorghum mosaic virus causing mosaic in *Miscanthus sinensis*. *Plant Dis.* **2012**, *96*, 150, doi: 10.1094/PDIS-07-11-0617. Kondaiah, E.; Nayudu, M.V. Sorgum mosaic virus strain H- a new record from India. *Curr. Sci.* **1984**, *53*, 273. Dosayla, R.D.; Benigno. D.A. A strain of sugarcane mosaic virus in Philippines. Proc. *Int. Soc. Sugarcane Technol.* **1980**,*17*,1510.	*Dactynotus ambrosiae, Rhopalosiphum maidis*	Koike, H.; Gillaspie Jr, A.G. Strain M, a new strain of sugarcane mosaic virus. *Plant Dis. Rep*. **1976**, *60*, 50-54. Another sorghum mosaic virus is identified in Brazil. Available online (https://www.embrapa.br/en/busca-de-noticias/-/noticia/34621747/another-sorghum-mosaic-virus-is-identified-in-brazil)

			Another sorghum mosaic virus is identified in Brazil. Available online: https://www.embrapa.br/en/busca-de-noticias/-/noticia/34621747/another-sorghum-mosaic-virus-is-identified-in-brazil. (Accessed on 28 March 2020). Muhammad, B.; Alegbejo, M.D.; Kashina, B.D.; Banwo, O.O. Occurrence and distribution of potyiruses infecting sorghum in Kaduna and Kano States, Nigeria. *Arch Phytopathol. PFL*. **2016,** *49*, 281-292, doi: 10.1080/03235408.2016.1180922.		
Soybean mosaic virus	D00507	Ethiopia, Morocco, South Africa, Tanzania, Uganda, Zambia, Zimbabwe, China, India, Iran, Iraq, Japan, Kazakhstan, Malaysia, Pakistan, Philippines, South Korea, Sri Lanka, Taiwan, Thailand, Turkey, Bulgaria, Croatia, Federal Republic of Yugoslavia, Germany, Italy, Moldova, Poland, Portugal, Romania, Russia, Serbia and Montenegro, Sweden, Ukraine, Canada, Jamaica, United States, Australia, New Zealand, Argentina, Brazil, Chile, Colombia, Ecuador, Venezuela	Plantwise KnowledgeBank on soybean mosaic virus. Available online:https://www.plantwise.org/knowledgebank/datasheet/48750.(Accessed on 28 March 2020).	*Dactynotus ambrosiae, Macrosiphum euphorbiae, Myzus persicae, Rhopalosiphum maidis, Aphis glycines, Aphis craccivora, Macrosiphum euphorbiae, Myzus persicae, Rhopalosiphum maidis, Rhopalosiphum padi, Aphis citricola, Aphis gossypii*	Abney, T.S.; Sillings, J.O.; Richards, T.L.; Broersma, D.B. Aphids and other insects as vectors of soybean mosaic virus. *J. Econ. Entomol.* **1976**, *69*, 254-256. Clark, A.J.; Perry, K.L. Transmissibility of field isolates of soybean viruses by *Aphis glycines*. *Plant Dis.* **2002**, *86*, 1219-1222, doi: 10.1094/PDIS.2002.86.11.1219. Halbert, S.E.; Irwin, M.E.; Goodman, R.M. Alate aphids (Homoptera: Aphididae) species and their relative importance as field vectors of soybean mosaic virus. *Ann. Appl. Biol.* **1981**, 97, 1-9, doi: 10.1111/j.1744-7348.1981.tb02988.x. Balgude, Y.; Sawant, D.M. Relationship of soybean mosaic virus with its aphid vectors. *Bioinfolet*, **2012**, 9, 61-65.
Spiranthes mosaic virus 3	AY685218	New Zealand, USA, UK	Tang, J.; Khan, S.; Quinn, B.; Veerakone, S.; Milleza, E.; Ward, L.I. First Report of spiranthes mosaic virus 3 in *Phlox spp.* in New Zealand. *Plant Dis.* **2017**, 101, 849-849, doi: 10.1094/PDIS-11-16-1681-PDN. Baker, C.A.; Webster, C.G.; Adkins, S. Spiranthes mosaic virus 3 and bidens mottle virus, two potyviruses detected in *Phlox divaricata*. *Plant Pathol. Circular No. 414*, **2014** First report of spiranthes mosaic virus 3 in the United Kingdom. Available online: https://gd.eppo.int/reporting/article-2567 (Accessed on 28 March 2020).	Unknown	
Sudan watermelon mosaic virus	KY623505	Sudan	Desbiez, C.; Verdin, E.; Tepfer, M., Scheibel, C.W.; Millot, P.; Dafalla, G.; Lecoq, H. Characterization of a new cucurbit-infecting ipomovirus from Sudan. *Arch. Virol.* **2016**, *161*, 2913–5, doi: 10.1007/s00705-016-2981-x.	Unknown	

Sugarcane mosaic virus	AJ297628	Angola, Cabo Verde, Cameroon, Congo, Democratic Republic of the Côte d'Ivoire, Egypt, Ethiopia, Ghana, Kenya, Madagascar, Malawi, Morocco, Nigeria, Réunion, Sierra Leone, South Africa, Tanzania, Uganda, Zambia, Zimbabwe, Bangladesh, Cambodia, China, India, Indonesia, Iran, Israel, Japan, Laos, Malaysia, Myanmar, Nepal, Pakistan, Philippines, Sri Lanka, Taiwan, Thailand, Turkey, Vietnam, Bulgaria, Czechia, Czechoslovakia, Germany, Greece, Hungary, Italy, Poland, Portugal, Romania, Serbia, Spain, Antigua and Barbuda, Barbados, Belize, Costa Rica, Cuba, Dominican Republic, El Salvador, Guadeloupe, Guatemala, Haiti, Honduras, Jamaica, Martinique, Mexico, Nicaragua, Panama, Puerto Rico, Saint Kitts and Nevis, Saint Vincent and the Grenadines, Trinidad and Tobago, U.S. Virgin Islands, United States,	Cabi datasheet on sugarcane mosaic virus. Available online: https://www.cabi.org/isc/datasheet/49801 (Accessed on 28 March 2020).	*Acyrthosiphon pisum, Aphis glycines, Aphis gossypii, Hysteroneura setariae, Macrosiphum euphorbiae, Metopolophium dirhodum, Myzus persicae, Rhopalosiphum maidis, Rhopalosiphum padi, Schizaphis graminum, Sitobion avenae, Uroleucon ambrosiae, Longiunguis sacchari, Melanaphis sacchari, Rhopalosiphum rufiabdominale*	Cabi datasheet on sugarcane mosaic virus. Available online: https://www.cabi.org/isc/datasheet/49801 (Accessed on 28 March 2020). Ghosh, A.; Chakrabarti, S.; Mandal, B.; Krishna Kumar, N.K. Aphids as vectors of the plant viruses in India. In *century of plant virology in India,* 1st.; Mandal, B., Pratap Rao, G., Baranwal, V.K., Jain, R.K., Eds.; Springer: Singapore, 2017; pp. 515- 536, ISBN 978-981-10-5671-0. Plant Wise Knowledge Bank on *Rhopalosiphum rufiabdominalis*. Available online: https://www.plantwise.org/knowledgebank/datasheet/47322. (Accessed on 9th June2020).

		Australia, Fiji, Papua New Guinea, Argentina, Bolivia, Brazil, Colombia, Ecuador, French Guiana, Paraguay, Peru, Suriname, Uruguay, Venezuela			
Sunflower chlorotic mottle virus	GU181199	Argentina	Rodríguez, M.; Taleisnik, E.; Lenardon, S.; Lascano, R. Are sunflower chlorotic mottle virus infection symptoms modulated by early increases in leaf sugar concentration? *J. Plant Physiol.* **2010**, *167*, 1137- 1144, doi: 10.1016/j.jplph.2010.03.004.	Unknown	
Sunflower mild mosaic virus	JQ350738	Argentina	Giolitti, F.; de Breuil, S.; Bejerman, N.; Lenardon, S. Complete nucleotide sequence and genetic organization of sunflower mild mosaic virus (SuMMoV). In Proceedings of the 18th international sunflower conference, Mar Del Plata & Balcarce, Argentina, 2012; pp. 997-1002.	Unknown	
Sunflower mosaic virus	AF465545	USA, India	Gulya, T.J.; Shiel, P.J.; Freeman, T.; Jordan, R.L.; Isakeit, T.; Berger, P.H. Host range and characterization of sunflower mosaic virus. *Phytopathology* **2002**, *92*, 694-702, doi: 10.1094/PHYTO.2002.92.7.694. Jindal, S.; Cheema, S.S.; Kang, S.S. Effect of sunflower mosaic virus on vigour and productivity in sunflower hybrids. *Plant Dis. Res.* **2001,** *16*, 79–83.	*Myzus persicae, Aphis gossypii, Aphis craccivora, Myzus persicae*	Verma, K.P.; Thakur, M.P.; Dantre, R.K. Occurrence of sunflower mosaic virus and its effect on yield contributing characters of sunflower. *J. Interacademicia* **2009**, *13*, 245–246. Bhat, B.N.; Reddy, D.R.R. Status of viruses infecting sunflower and strategies for their management. *In plant viruses: evolution and management*; Gaur R., Petrov N., Patil B., Stoyanova M. Eds.; Springer: Singapore, 2016 ISBN 978-981-10-1406-2.
Sunflower ring blotch virus	KX856009	Argentina	Cabrera Mederos, D.; Bejerman, N..; Trucco, V.; de Breuil, S.; Lenardon, S.; Giolitti, F. Complete genome sequence of sunflower ring blotch virus, a new potyvirus infecting sunflower in Argentina. *Arch Virol.* **2017,** 162, 1787-1790, doi: 10.1007/s00705-017-3275-7.	Unknown	
Sweet potato feathery mottle virus	D86371	Argentina, Brazil, Chile, Peru, Venezuela, Australia, Fiji, French Polynesia, New Zealand, Solomon Islands, Tonga, Canada, Costa Rica, United States, China, India, Israel, Japan, South Korea, Syria, Taiwan, Vietnam, Italy,	Cabi datasheet on Sweet potato feathery mottle virus (SPFMV). Available online: https://www.cabi.org/isc/datasheet/50963 (Accessed on 28 March 2020).	*Aphis gossypii, Myzus persicae*	Wosula, E.N.; Clark, C.A.; Davis, J.A. Effect of host plant, aphid species, and virus infection status on transmission of sweet-potato feathery mottle virus. *Plant Dis*. **2012**, *96*, 1331-1336, doi: 10.1094/PDIS-11-11-0934-RE.

		Spain, Cameroon, Congo, Egypt, Ethiopia, Kenya, Madagascar, Niger, Nigeria, Rwanda, South Africa, Tanzania, Togo, Uganda, Zambia, Zimbabwe			
Sweet potato latent virus	KC443039	Kenya, Rwanda, Uganda, China, Japan, South Korea, Taiwan, Brazil	Cabi, Plantwise KnowledgeBank on sweet potato latent virus. Available online: https://www.plantwise.org/knowledgebank/datasheet/18604 (Accessed on 28 March 2020).	Unknown	
Sweet potato mild speckling virus	U61228	Brazil, Argentina, Peru, Indonesia, South Africa, Tanzania, Uganda, Ethiopia, China	Alvarez, V.; Ducasse, D.A.; Biderbost, E.; Nome, S.F. Sequencing and characterization of the coat protein and non- coding region of a new sweet potato potyvirus. *Arch. Virol.* **1997**, *142*, 1635-1644, doi: 10.1007/s007050050185. Nome, C.F.; Laguna, I.G.; Nome, S.F. Cytological alterations produced by sweet potato mild speckling virus. *J. Phytopathology* **2006**, *154*, 504-507, doi: 10.1111/j.1439-0434.2006.01131.x. Salazar, L. Identification and characterization of sweetpotato viruses and search for resistance. Annual Subproject Progress Report (0801) for Peru. CIP, Lima, Peru, 1998. Prain, G.; Machmud, M.; Rusmadi. Evaluation of virus diseases. Annual subproject progress report (080304) for Indonesia. CIP, Lima, Peru, 1998. Domola, M.J.; Thompson, G.J.; Aveling, T.A.S.; Laurie, S.M.; Strydom, H.; Van den Berg, A.A. Sweet potato viruses in South Africa and the effect of viral infection on storage root yield. *Afr. Plant Prot.* **2008**, *14*, 15-23. Ndunguru, J.; Kapinga, R.; Sseruwagi, P.; Sayi, B.; Mwanga, R.; Tumwegamire, S.; Rugutu, C. Assessing the sweet potato virus disease and its associated vectors in northwestern Tanzania and central Uganda. *Afr. J. Agric. Res.* **2009**, *4*, 334-343, doi: 10.1021/jf802872b. Tesfaye, T.; Feyissa, T.; Abraham, A. Survey and serological detection of sweet potato (*Ipomoea batatas* (L.) Lam) viruses in Ethiopia. *J. Appl. Biosciences* **2011**, 41, 2746–2756. Zhang, L.M.; Wang, Q.M.; Ma, D.F.; Wang, Y. The effect of major viruses and virus-free planting materials on sweetpotato root yield in China. *Acta Hortic.* **2006**, 703, 71–78, doi: 10.17660/ActaHortic.2006.703.7.	Unknown	
Sweet potato virus 2	JN613807	Portugal, New Zealand, USA, Taiwan, Australia, Spain, Peru, South Africa, Zambian	Varanda, C.M.; Santos, S.J.; Oliveira, M.D.; Clara, M.I.; Félix, M.R. Detection of sweet potato virus C, sweet potato virus 2 and sweet potato feathery mottle virus in Portugal. *Acta Virol*. **2015,** 59, 185-8, doi: 10.4149/av_2015_02_185. Perez-Egusquiza, Z.; Ward, L.; Clover, G.R.G.; Fletcher, J.D. Detection of Sweet potato virus 2 in sweet potato in New Zealand. *Plant Dis*. **2009,** 93, 427, doi: 10.1094/PDIS-93-4-0427B.	*Aphis gossypii*, *Myzus persicae*	Souto, E.R.; Sim, J.; Chen, J.; Valverde, R.A.; Clark, C.A. Properties of strains of sweet potato feathery mottle virus and two newly recognized potyviruses infecting sweet potato in the United States. *Plant Dis.* **2003**, *87*, 1226-1232, doi: 10.1094/PDIS.2003.87.10.1226.

			Almeyda, C.V.; Abad, J.A.; Pesic-VanEsbroeck, Z. First Report of sweet potato virus G and sweet potato virus 2 infecting sweetpotato in North Carolina. *Plant Dis.* **2013**, 97, 1516-1516, doi: 10.1094/PDIS-04-13-0359-PDN. Ateka, E.M.; Barg, E.; Njeru, R.W.; Lesemann, D.E.; Vetten, H.J. Further characterization of 'sweet potato virus 2': A distinct species of the genus Potyvirus. *Arch. Virol.* **2004**, *149*, 225-239, doi: 10.1007/s00705-003-0233-3. Tairo, F.; Jones, R.A.C.; Valkonen, J.P.T. Potyvirus complexes in sweetpotato: Occurrence in Australia, serological and molecular resolution, and analysis of the sweet potato virus 2 (SPV2) component. *Plant Dis.* **2006**, *90*, 1120-1128, doi: 10.1094/PD-90-1120. Trendo, H.P.; Lozano, G.; Valverde, R.A.; Navas-Castillo, J., First report of sweet potato virus G and sweet potato virus 2 infecting sweet potato in Spain. *Plant Dis.* **2007**, *91*,1687, doi: 10.1094/PDIS-91-12-1687C. Untiveros, M.; Fuentes, S.; Kreuze, J. Molecular variability of sweet potato feathery mottle virus and other potyviruses infecting sweet potato in Peru. *Arch. Virol.* **2008**, *153*, 473-483, doi: 10.1007/s00705-007-0019-0. Ateka, E.; Barg, E.; Njeru, R. et al. Biological and molecular variability among geographically diverse isolates of sweet potato virus 2. *Arch Virol.* **2007**, *152*, 479–488, doi: 10.1007/s00705-006-0879-8.		
Sweet potato virus C	GU207957	Portugal, Australia, USA, Peru, Israel, Kenya, Korea, China, Honduras, Guatemala	Varanda, C.M.; Santos, S.J.; Oliveira, M.D.; Clara, M.I.; Félix, M.R. Detection of sweet potato virus C, sweet potato virus 2 and sweet potato feathery mottle virus in Portugal. *Acta Virol.* **2015**, *59*, 185-8, doi: 10.4149/av_2015_02_185. Maina, S.; Barbetti, M.J.; Edwards, O.R; Almeida, de L.; Ximenes, A.; Jones, R.A.C. Sweet potato feathery mottle virus and sweet potato virus C1 from East Timorese and Australian Sweetpotato: Biological and Molecular properties, and Biosecurity implications. *Plant Dis.* Accepted for publication. Clark, C.A.; Davis, J.A.; Abad, J.A.; Cuellar, W.J.; Fuentes, S.; Kreuze, J.F.; Gibson, R.W.; Mukasa, S.B.; Tugume, A.K.; Tairo, F.; Valkonen, J.P.T. Sweetpotato viruses: 15 years of progress on understanding and managing complex diseases. *Plant Dis.* **2012**, *96*, 168- 185, doi: 10.1094/PDIS-07-11-0550. Untiveros, M.; Quispe, D.; Kreuze, J. Analysis of complete genomic sequences of isolates of the sweet potato feathery mottle virus strains C and EA: Molecular evidence for two distinct potyvirus species and two P1 protein domains. *Arch. Virol.* **2010**, *155*, 2059-2063, doi: 10.1007/s00705-010-0805-y. Prakash, S.; Tam, Y.; Zeidan, M.; Abu-Ras, A.; Gaba, V. First report of sweet potato virus C infecting sweet potato in Israel. *New Dis. Rep*. **2013**, *28*, *4*, doi: 10.5197/j.2044-0588.2013.028.004. Wainaina, J.M.; Ateka, E.; Makori, T.; Kehoe, M.A.; Boykin, L.M. Phylogenomic relationship and evolutionary insights of sweet potato viruses from the western highlands of Kenya. *Peer J.* **2018**, *6*, e5254, doi: 10.7287/peerj.preprints.26909v1. Jo, Y.; Kim, S.; Choi, H.; Yang, J.W.; Lee, B.C.; Cho, W.K. Sweet potato viromes in eight different geographical regions in Korea and two different cultivars. *Sci. Rep*. **2020**,*10*,2588, doi: 10.1038/s41598-020-59518-x.	*Myzus persicae*	Wosula, E.N. Dynamics of the sweetpotato potyvirus aphid pathosystem in Louisiana. PhD dissertation, Louisiana State University, Louisiana, USA, 2012.

			Bao, G.; Zuo, R.; Rao, W.; Li, R.; Li, F. Detection of sweet potato viruses in Yunnan and genetic diversity analysis of the common viruses. *Microbiol. China*. **2013**, *40*, 236-248.<br>Ana Sofia, A. Single and mixed infections of plant RNA and DNA viruses are prevalent in commercial sweet potato in Honduras and Guatemala. Master's thesis, University of Arizona, Arizona, USA, 2015.		
Sweet potato virus G	IQ824374	USA, Peru, South Africa, Spain, China, Honduras, Guatemala, Brazil, USA, Egypt, Ethiopia, Tahiti, Tubuai, South Africa, Hawaii, Indonesia, New Zealand, Australia, Zimbabwe, Ghana, Timor, South Korea, Egypt, Japan, Taiwan	Untiveros, M.; Fuentes, S.; Kreuze, J. Molecular variability of sweet potato feathery mottle virus and other potyviruses infecting sweet- potato in Peru. *Arch. Virol.* **2008**, *153*, 473-483, doi: 10.1007/s00705-007-0019-0.<br>Parsad, B.J.; Gubba, A. Identification and distribution of viruses infecting sweet potato (*Ipomoea batatas*) in KwaZulu-Natal, South Africa. *South Afr. J. Plant and Soil* **2013**, *30*, 179-190, doi: 10.1080/02571862.2013.854415.<br>Trenado, H.P.; Lozano, G.; Valverde, R.A.; Navas-Castillo, J. First report of sweet potato virus G and sweet potato virus 2 infecting sweet potato in Spain. *Plant Dis.* **2007**, *91*, 1687, doi: 10.1094/PDIS-91-12-1687C.<br>Bao, G.; Zuo, R.; Rao, W.; Li, R.; Li, F. Detection of sweet potato viruses in Yunnan and genetic diversity analysis of the common viruses. *Microbiol. China* **2013**, *40*, 236-248.<br>Ana Sofia, A. Single and Mixed Infections of Plant RNA and DNA Viruses are Prevalent in Commercial Sweet Potato in Honduras and Guatemala. Master's thesis, University of Arizona, Arizona, USA, 2015.<br>Fernandes, F.R.; Dusi, A.N. Viroses da batata-doce no Brasil: importância e principais medidas de controle. *Circular Técnica* **2013**, *126*.<br>Souza, C.A.; Rossato, M.; Melo, F.L.; Pereira-Carvalho, R.C. Complete genome sequences of sweet potato feathery mottle virus and sweet potato virus G from Brazil. *Genome Announc.* **2018**, *6*, e01567, doi: 10.1128/genomeA.01567-17.<br>Almeyda, C.V.; Abad, J.A.; Pesic-VanEsbroeck, Z. First Report of sweet potato virus G and sweet potato virus 2 Infecting sweetpotato in North Carolina. *Plant Dis.* **2013,** *97*,1516, doi: 10.1094/PDIS-04-13-0359-PDN.<br>IsHak, J.A.; Kreuze, J.F.; Johansson, A.; Mukasa, S.B.; Tairo, F.; Abo El-Abbas, F.M.; Valkonen, J.P.T. Some molecular characteristics of three viruses from SPVD-affected sweet potato plants in Egypt. *Arch. Virol.* **2003**, *148*, 2449-2460, doi: 10.1007/s00705-003-0187-5.<br>Alemu, T. Characterisation of Viruses of Pepper (Capsicum spp.) and sweet Potato (Ipomoea batatas) from Ethiopia. Ph.D. thesis. Rheinischen Friedrich-Wilhelms-Universität zu Bonn, Göttingen, Germany. (in German), 2004.<br>Rännäli, M.; Czekaj, V.; Jones, R.A.C.; Fletcher, J.D.; Davis, R.I.; Mu, L.; Dwyer, G.I.; Coutts, B.A.; Valkonen, J.P.T. Molecular genetic characterization of sweet potato virus G (SPVG) isolates from areas of the pacific ocean and southern Africa. *Plant Dis.* **2008**, *92*, 1313-1320, 10.1094/PDIS-92-9-1313.<br>Sossah, F.L.; Appiah, A.S.; Oduro, V.; Amoatey, H.M.; Owusu, G.K.; Oppong, A.; Lamptey, J.N.L.; Carey, E.E.; Fuentes, S. Incidence of sweetpotato viruses in the	*Aphis gossypii*, *Myzus persicae*	Souto, E.R.; Sim, J.; Chen, J.; Valverde, R.A.; Clark, C.A. Properties of strains of Sweet potato feathery mottle virus and two newly recognized potyviruses infecting sweet potato in the United States. *Plant Dis.* **2003**, *87*, 1226-1232, doi: 10.1094/PDIS.2003.87.10.1226.

			coastal savannah agro-ecological zone of Ghana. *J. Plant Pathol.* **2015,** *97*, 109-117, doi: 10.4454/JPP.V97I1.030. Maina, S.; Edwards, O.R.; Barbetti, M.J.; de Almeida, L.; Ximenes, A.; Jones, R.A.C. Deep sequencing reveals the complete genome sequence of sweet potato virus G from East Timor. *Genome Announc.* **2016**, 4, e00957-16, doi:10.1128/genomeA.00957-16. Kwak, H.R.; Kim, J.; Kim, M.K.; Seo, J.K.; Jung, M.N.; Kim, J.S.; Lee, S.; Choi, H.S. Molecular characterization of five potyviruses infecting Korean sweet potatoes based on analyses of complete genome sequences. *Plant Pathol. J.* **2015**, *31*, 388-401, doi: 10.5423/PPJ.OA.04.2015.0072. IsHak, J.A.; Kreuze, J.F.; Johansson, A.; Mukasa, S.B.; Tairo, F.; Abo ElAbbas, F.M.; Valkonen, J.P.T. Some molecular characteristics of three viruses from SPVD-affected sweet potato plants in Egypt. *Arch. Virol*. **2003**, *148*, 2449-2460, doi: 10.1007/s00705-003-0187-5. Sakai, J.; Yamasaki, S.; Ogawa, T.; Ohnuki, M.; Hanada, K. Detection of sweetpotato virus G by RT-PCR from sweetpotato in Kyushu. *Ann. Phytopath. Soc. Jpn.* **1997**, *63*, 484. Wang, L.Y.; Cheng, Y.H.; Wang, N.Y.; Chen, K.C.; Yeh, S.D. First report of sweet potato virus G infecting sweet potato in Taiwan. *Plant Dis.* **2013**, *97*,1260, doi: 10.1094/PDIS-01-13-0111-PDN.	
Tamarillo leaf malformation virus	KM523548	Columbia	Villegas, M.D.; Montoya, M.M.; Gutiérrez, P.A. Genome comparison and primer design for detection of tamarillo leaf malformation virus (TaLMV). *Arch Phytopathol. Plant Prot.* **2017,** *50*, 713-726, doi: 10.1080/03235408.2017.1370934.	Unknown
Telfairia mosaic virus		Nigeria	Nwauzo, E.E.; Brown, Jr W.M. Telfairia (Cucurbitaceae) mosaic virus in Nigeria. *Plant Dis. Rep.* **1975**, *59*, 430-432.	Unknown
Telosma mosaic virus	DQ851493	Vietnam, Indonesia, Thailand, China	Ha, C.; Coombs, S.; Revill, P.A.; Harding, R.M.; Vu, M.; Dale, J.L. Design and application of two novel degenerate primer pairs for the detection and complete genomic characterization of potyviruses. *Arch Virol.* **2008**, *153,* 25-36, doi: 10.1007/s00705-007-1053-7. Noveriza, R.; Suastika, G.; Hidayat, S.H.; Kartosuwondo, U. Potyvirus associated with mosaic disease on patchouli (*Pogostemon cablin* (Blanco) Benth.) plants in Indonesia. *J ISSAAS.* **2012,** *18*, 131-46. Chiemsombat, P.; Prammanee, S.; Pipattanawong, N. Occurrence of telosma mosaic virus causing passion fruit severe mosaic disease in Thailand and immunostrip test for rapid virus detection. *Crop Prot.* **2014,** *63*, 41–7, doi: 10.1016/j.cropro.2014.04.023. Lixue, X.; Xiaoyan, Z.; Shan, Z.; Lijie, Z.; Tao L. Molecular identification and specific detection of telosma mosaic virus infecting passion fruit. *Sci. Agric. Sin.* **2017**, 50, 4725-34, doi: 10.3864/j.issn.0578-1752.2017.24.006.	Unknown
Thunberg fritillary mosaic virus	AJ851866	China	Wei, C.B.; Chen, J.; Zhang, Q.Y.; Shi, Y.H.; Lin, L.; Zheng, H.Y.; Adams, M.J.; Chen, J.P. A new potyvirus from thunberg fritillary (*Fritillaria thunbergii* Miq.) in Zhejiang, China. *Arch Virol.* **2005,** 150, 1271-80, doi: 10.1007/s00705-005-0519-8.	Unknown

Tobacco etch virus	M11458	Nigeria, Sudan, Tunisia, China, India, Singapore, Turkey, Cyprus, France, Hungary, Russia, Spain, Canada, Cuba, El Salvador, Guatemala, Jamaica, Mexico, Puerto Rico, Trinidad and Tobago, United States, Venezuela	PlantWise KnowledgeBank on tobacco etch virus. Available online: https://www.plantwise.org/knowledgebank/datasheet/54134 (Accessed on 28 March 2020)	*Myzus persicae, Macrosiphum euphorbiae, Aphis fabae, Aphis gossypii, Aphis craccivora, Aphis spiraecola, Lipaphis pseudobrassicae, Uroleucon ambrosiae, Aphis amaranthi,*	Kassanis. B. Transmission of tobacco etch viruses by aphid. *Ann. appl. Biol.* **1941**, *28*, 238. McDonald, S.; Halbert, S.; Tolin, S.; Nault, B. Seasonal abundance and diversity of aphids (homoptera: aphididae) in a pepper production region in Jamaica. *Environ. Entomol.* **2003**, *32*, 499-509, doi: 10.1603/0046-225X-32.3.499. McDonald, S.A. Epidemiology, aphid vectors, impact and management of tobacco etch potyvirus in hot peppers in Jamaica. PhD thesis, Virginia Tech, 2014.
Tobacco mosqueado virus	KT834407	Brazil	Blawid, R.; Rodrigues, K.B.; de Moraes Rêgo, C.; Inoue-Nagata, A.K.; Nagata, T. Complete genome sequence of tobacco mosqueado virus. *Arch Virol.* **2016,** *16*, 2619-22, doi: 10.1007/s00705-016-2956-y.	Unknown	
Tobacco vein banding mosaic virus	EF219408	USA, Japan, Taiwan, China	Reddick, B.B.; Collins-Shepard, M.H.; Christie, R.G.; Gooding, G.V. A new virus-disease in North America caused by tobacco vein-banding mosaic virus. *Plant Dis.* **1992**, *76*, 856-859, doi: 10.1094/PD-76-0856. Chin, W.T. A survey of tobacco mosaic viruses in central Taiwan. *J. Agric. Ass. Chin.* **1966**, *55*, 85–88. Roggero, P.; Accotto, G.P.; Ciuffo, M.; Lenzi, R.; Desbiez, C.; Lecoq, H.; Bosco, D.; Huang, X.; Gu, Q. First report of tobacco vein banding mosaic virus in China (Xian, Shaanxi Province) in *Datura stramonium* and tobacco. *Plant Dis.* **2000**, *84*, 1152 doi: 10.1094/PDIS.2000.84.10.1152D.	*Myzus persicae, Aphis rumicis, Aulacorthum nipponicum, Aphis craccivora, Brachycaudus helichrysi, Aphis nerii, Aphis glycines, Capitophorus hippophaes, Hyalopterus pruni, Rhopalosiphum maidis, Rhopalosiphum padi, Rhopalosiphum rufiabdominalis, Lipaphis erysimi*	Fang, H.S.; Nee, H.H.; Chou, T.G. Comparative ability of seventeen aphid species to transmit tobacco vein banding mosaic virus. *Bull. Taiwan Tobacco Res. Inst.* **1985**, *22*, 41-46.
Tobacco vein mottling virus	X04083	USA	Pirone, T.P.; Gooding, G.V.; Smiley, J.H. Tobacco vein mottling virus on burley tobacco in Kentucky. *Plant Dis. Reptr.* **1973**, *57*, 841-844.	*Myzus persicae, Macrosiphum euphorbiae, Aphis craccivora, Aphis gossypii, Rhopalosiphum maidis, Rhopalosiphum padi*	Pirone, T.P.; Gooding, Jr., G.V.; Smiley, J.H. Tobacco vein mottling virus on burley tobacco in Kentucky. *Plant Dis. Reptr.* **1973**, *57*, 841-844. Kennedy, G.G.; Wade, D.; Gooding, G.V. Previously unreported aphid vectors of tobacco vein mottling virus. *Phytopathology* **1979**, *69*, 529.

Tomato necrotic stunt virus	JQ314463	Mexico	Li, R.; Gao, S.; Hernandez, A.G.; Wechter, W.P.; Fei, Z.; Ling, K.S. Deep sequencing of small RNAs in tomato for virus and viroid identification and strain differentiation. *PLoS ONE*, **2012**, 7, e37127, doi: 10.1371/journal.pone.0037127.	Unknown	
Tradescantia mild mosaic virus	AY861351	New Zealand, Italy	Tang, J.; Veerakone, S.; Ward, L.I. First report of tradescantia mild mosaic virus in *Tradescantia spp*. in New Zealand. *Plant Dis*. **2016**, *100*, 1032-1032, doi: 10.1094/PDIS-11-15-1287-PDN. Ciuffo, M.; Masenga, V.; Turina, M. Characterization of a potyvirus isolated from *Tradescantia fluminensis* in northern Italy. *Arch Virol*. **2006**, *151*,1235-1241, doi: 10.1007/s00705-005-0691-x.	Unknown	
Tuberose mild mosaic virus	AF062926	Taiwan, China, India	Chen, C. C.; Chang, C. A. Characterization of a potyvirus causing mild mosaic on tuberose. *Plant Dis*. **1998**, *82*, 45-49, doi: 10.1094/PDIS.1998.82.1.45. Lin, L.; Zheng, H.Y.; Chen, J.; Chen, J.P.; Zhang, Q.Y.; Zhao, M.F.; Antoniw, J.F.; Adams, M.J. A new potyvirus from tuberose (*Polianthes tuberosa*) in China. *Arch. Virol*. **2004**, *149*, 1107-1116, doi: 10.1007/s00705-003-0289-0. Raj, S.K.; Snehi, S.K.; Kumar, S.; Ram, T.; Goel, A.K. First report of tuberose mild mosaic potyvirus from tuberose (*Polianthes tuberosa* L.) in India. *Australas. Plant Dis. Notes* **2009**, *4*, 93-95, doi: 10.1071/DN09040.	*Myzus persicae*	Chen, C.C.; Chang, C.A. Characterization of a potyvirus causing mild mosaic on tuberose. *Plant Dis*. **1998**, *82*, 45-49, doi: 10.1094/PDIS.1998.82.1.45.
Tuberose mild mottle virus	AJ581528	China, India, New Zealand, USA	Lin, L.; Zheng, H.Y.; Chen, J.; Chen, J.P.; Zhang, Q.Y.; Zhao, M.F.; Antoniw, J.F.; Adams, M.J. A new potyvirus from tuberose (*Polianthes tuberosa*) in China. *Arch Virol*. **2004**, *149*, 1107-16, doi: 10.1007/s00705-003-0289-0. Kulshrestha, S.; Mehra, A.; Hallan, V.; Raikhy, G.; Ram, R.; Zaidi, A.A. Molecular evidence for occurrence of Tuberose mild mottle virus infecting tuberose (*Polianthes tuberosa*) in India. *Curr. Sci*. **2005**, *89*, 870–872 Pearson, M.N.; Horner, M.B. A potyvirus of *Polyanthes tuberose* in New Zealand. *Australas Plant Pathol*. **1986**, *15*, 39, doi: 10.1071/APP9860039. Dey, K.K.; Melzer, M.J.; Li, C.; Sun, X.; Adkins, S. First report of Tuberose mild mottle virus infecting tuberose (*Polianthes tuberosa*) in the United States. *Plant Dis*. **2018**, *102*, 461. doi: 10.1094/PDIS-07-17-1024-PDN.	*Myzus persicae*	Kulshrestha, S.; Mehra, A.; Hallan, V.; Raikhy, G.; Ram, R.; Zaidi, A.A. Molecular evidence for occurrence of Tuberose mild mottle virus infecting tuberose (*Polianthes tuberosa*) in India. *Cur. Sci*. **2005**, *89*, 870-872
Tulip breaking virus	KF826466	Japan, Czechoslovakia, Denmark, Netherlands, United Kingdom, United States, Australia	Plantwise KnowledgeBank on tulip breaking virus. Available online: https://www.plantwise.org/KnowledgeBank/datasheet/55319 (Accessed online on 28 March 2020).	*Myzus persicae, Aphis gossypii, Aphis fabae, Macrosiphum euphorbiae, Dysaphis tulipae, Aulocorthum circumflexum*	Lesnaw, J.A.; Ghabrial, S.A. Tulip breaking: past, present, and future. *Plant Dis*. **2000**, *84*, 1052-60, doi: 10.1094/PDIS.2000.84.10.1052. Hammond, J.; Chastagner, G.A. Field transmission of tulip breaking virus and serologically related potyviruses in tulip. *Plant Dis*. **1989**, *73*, 331-336, doi: 10.1094/PD-73-0331.
Tulip mosaic virus	X63630			Unknown	
Turnip mosaic virus	AF169561	Kenya, Mauritius, Morocco, South Africa, Zimbabwe, China, India, Iran, Israel,	Cabi datasheet on turnip mosaic virus. Available online: https://www.cabi.org/ISC/datasheet/54306 (Accessed on 28 March 2020).	*Aphis gossypii, Brevicoryne brassicae, Lipaphis erysimi,*	Cabi datasheet on turnip mosaic virus. Available online: https://www.cabi.org/ISC/datasheet/54306 (Accessed on 28 March 2020).

		Japan, Nepal, Philippines, Saudi Arabia, Singapore, South Korea, Taiwan, Thailand, Turkey, Uzbekistan, Vietnam, Yemen, Austria, Belgium, Bulgaria, Czechia, Czechoslovakia, Denmark, Federal Republic of Yugoslavia, France, Germany, Greece, Hungary, Italy, Lithuania, Netherlands, Poland, Portugal, Romania, Russia, Spain, Sweden, United Kingdom, Canada, Trinidad and Tobago, United States, Australia, New Zealand, Solomon Islands, Argentina, Brazil, Venezuela		*Myzus persicae, Rhopalosiphum maidis*
Twisted-stalk chlorotic streak virus	AY954248	USA	Robertson, N.L. A newly described plant disease complex involving two distinct viruses in a native Alaskan lily, *Streptopus amplexifolius*. **2019** (Unpublished, GenBank Acc. No. NC_043169)	Unknown
Vallota mosaic virus	FJ618540	Netherlands, USA, UK, New Zealand.	Nouye, N.; Hakkaart, F.A. Preliminary description of a potyvirus from *Vallota speciosa*. *Neth. J. Plant Path.* **1980**, *68*, 265-75, doi: 10.1007/BF01977302. USA (Unpublished, GenBank Acc. No. EF441726). Monger, W.A.; Mumford, R.A. Vallota mosaic virus infecting nerine in the UK. *New Dis Rep*. **2007,** *16*, 32, doi: 10.1111/j.1365-3059.2008.01850.x. Cohen, D.; Pearson, M.N.; Cowell, S.J.; Jones, D.; Blouin, A.; Lebas, B.S.M.; Shiller, J.B.; Clover, G.R. **2019**. A survey of viruses of flower bulbs in New Zealand (Unpublished, GenBank Acc. No. NC_043170).	Unknown
Vanilla distortion mosaic virus	KF906523	India	Balaji, C.G.; Aravintharaj, R.; Nagendran, K.; Priyanka, R.; Karthikeyan, G. First report of vanilla distortion mosaic virus (vdmv) in ornamental *Zinnia bicolor* in India. *J. Plant Pathol.* **2014**, *96*, 131, doi: 10.4454/JPP.V96I4.009.	Unknown

Verbena virus Y	EU564817	USA	Kraus, J.; Cleveland, S.; Putnam, M.L.; Keller, K.E.; Martin, R.R.; Tzanetakis, I.E. A new potyvirus sp. infects verbena exhibiting leaf mottling symptoms. *Plant Dis.* **2010,** *9*, 1132-1136, doi: 10.1094/PDIS-94-9-1132.	*Myzus persicae*	Kraus, J.; Cleveland, S.; Putnam, M.L.; Keller, K.E.; Martin, R.R.; Tzanetakis, I.E. A new potyvirus sp. infects verbena exhibiting leaf mottling symptoms. *Plant Dis.* **2010,** *9*, 1132-1136, doi: 10.1094/PDIS-94-9-1132.
Watermelon leaf mottle virus	AF028004	USA	De Sa, P.B.; Hiebert, E.; Purcifull, D.E. Molecular characterization and coat protein serology of watermelon leaf mottle virus (Potyvirus). *Arch Virol*. **2000**, *145*, 641-650, doi: 10.1007/s007050050053.	*Myzus persicae*	De Sa, P.B.; Hiebert, E.; Purcifull, D.E. Molecular characterization and coat protein serology of watermelon leaf mottle virus (Potyvirus). *Arch Virol*. **2000**, *145*, 641-650, doi: 10.1007/s007050050053.
Watermelon mosaic virus	AY437609	Egypt, Kenya, Libya, Mauritius, Morocco, South Africa, Sudan, Tunisia, Bangladesh, China, India, Indonesia, Iran, Iraq, Israel, Japan, Jordan, Kazakhstan, Kuwait, Lebanon, Oman, Pakistan, Philippines, Saudi Arabia, South Korea, Syria, Taiwan, Turkey, Yemen, Bosnia and Herzegovina, Bulgaria, Cyprus, Czechia, Czechoslovakia, France, Germany, Greece, Hungary, Italy, Poland, Russia, Serbia, Serbia and Montenegro, Slovenia, Spain, Ukraine, Canada, Costa Rica, Cuba, Guadeloupe, Honduras, Jamaica, Martinique, Mexico, United States, Australia, Cook Islands, Federated States of Micronesia, Fiji, French Polynesia, Kiribati, New Zealand,	Cabi datasheet on watermelon mosaic virus. Available online: https://www.cabi.org/isc/datasheet/56821 (Accessed on 24 March 2020).	*Aphis citricola, Aphis craccivora, Aphis gossypii, Aulacorthum solani, Macrosiphum euphorbiae, Myzus persicae, Toxoptera citricidus, Aphis fabae, Uroleucon pseudambrosiae, Aphis illinoisensis, Rhopalosiphum nymphaeae, Tetraneura hirsute, Rhobodium porosium, Uroleucon formosanus, Uroleucon gobonis, Capitophorus elaeagni, Cryptomyzus ribis*	Yamamoto, T. Infection cycle of watermelon mosaic virus. *J.A.R.Q* **1986**, *19*, 259-265. Al-Musa, A.; Mansour, A. Some properties of a watermelon mosaic virus in Jordan. *Plant Dis.* **1982**, *66*, 330-331. Webb, S.E., Kok-Yokomi, M.L. Transmission of cucurbit potyviruses by *Uroleucon pseudambrosiae* (Homoptera: Aphididae), an aphid trapped during epidemics of watermelon mosaic virus 2 in Florida. *J. Econ. Entomol.* **1993**, *86*, 1786-1792, doi: 10.1093/jee/86.6.1786. Adlerz, W.C. Cucurbit potyvirus transmission by alate aphids (Homoptera: Aphididae) trapped alive. *J. Econ. Entomol.* **1987**, 80, 87-92, doi: 10.1093/jee/80.1.87 Perring,T.M.; Farrar, C.A.; Mayberry, K.; Blua, M.J. Research reveals pattern of cucurbit virus spread. *Calif. Agr.* **1992**, *46*, 35-40. Halbert, S.E.; Corsini, D.L.; Wiebe, M. A. *Potato virus Y* transmission efficiency for some common aphids in Idaho. *Am. J. Potato Res.* **2003**, *80*, 87-91, doi: 10.1007/BF02870207. Awasthi, L.P. *Applied Plant Virology: Advances, Detection, and Antiviral Strategies*, 1st ed.; Academic Press: Cambridge, MA, USA, 2020, ISBN: 978-01-281-8654-1.

		Samoa, Solomon Islands, Tonga, Argentina, Brazil, Chile, Suriname, Venezuela			
Wild melon vein banding virus	KY623506	Sudan	Desbiez, C.; Wipf-Scheibel, C.; Millot, P.; Verdin, E.; Dafalla, G.; Lecoq, H. New species in the papaya ringspot virus cluster: Insights into the evolution of the PRSV lineage. *Virus Res.* **2017**, *241*, 88-94, 10.1016/j.virusres.2017.06.022.	Unknown	
Wild onion symptomless virus	LC159494	Japan	Ohshima, K.; Korkmaz, S.; Mitoma, S.; Nomiyama, R.; Honda, Y. First genome sequence of wild onion symptomless virus, a novel member of potyvirus in the turnip mosaic virus phylogenetic group. *Genome Announc*. 2016, 4, e00851-16, doi: 10.1128/genomeA.00851-16.	Unknown	
Wild potato mosaic virus	AJ437279	Peru	Jones, R.A.C.; Fribourg, C.E. Host plant reactions, some properties, and serology of wild potato mosaic virus. *Phytopathology* **1979**, *69*, 446–449, doi: 10.1094/Phyto-69-446.	Unknown	
Wild tomato mosaic virus	DQ851495	Vietnam, Thailand, China	Ha, C.; Revill, P.; Harding, R.M.; Vu, M.; Dale, J.L. Identification and sequence analysis of potyviruses infecting crops in Vietnam. *Arch. Virol.* **2008,** *153*, 45-60, doi: 10.1007/s00705-007-1067-1.<br>Du, Z.; She, X.; Tang, Y.; He, Z.F.; Yang, J. First report of Wild tomato mosaic virus infecting tobacco (*Nicotiana tabacum*) in China. *Plant Dis.* **2014**, *98*, 856, doi: 10.1094/PDIS-09-13-0927-PDN.	Unknown	
Wisteria vein mosaic virus	AY656816	USA, China, Australia, Iran, UK, Netherlands, New Zealand, Poland	Halliwell, R.S.; Porterfield, M.R. First report of a potyvirus infecting wisteria floribunda in the United States. *Plant Dis.* **1984**, *68*, 450, doi: 10.1094/PHP-2008-0818-01-BR.<br>Liang, W.X.; Song, L.M.; Li, Y.; Tian, G.Z.; Li, H.F.; Fan, Z.F. First report of wisteria vein mosaic virus in China. *Plant Pathol.* **2004,** *53*, 516, doi: 10.1007/s42161-019-00318-2<br>Clover, G.R.G.; Tang, Z.; Smales, T.E.; Pearson, M.N. Taxonomy of wisteria vein mosaic virus and extensions to its host range and geographical distribution. *Plant Pathol.* **2003**, *52*, 92–96, doi: 10.1046/j.13653059.2003.00798.x.<br>Al Jaberi, M.; Zakiaghl, M.; Mehrvar, M. First report of wisteria vein mosaic virus on *Wisteria sinensis* in Iran. *New Dis. Rep.* **2018**, *38*, 18, doi: 10.5197/j.2044-0588.2018.038.018.<br>Clover, G.R.G.; Denton, J.O.; Denton, G.J. First report of wisteria vein mosaic virus on *Wisteria spp.* in the United Kingdom. *New Dis. Rep.* **2015**, *31*, 1, doi: 10.5197/j.2044-0588.2015.031.001.<br>Ward, L.I.; Tang, J.Z.; Clover, G.R.G. First report of wisteria vein mosaic virus on *Wisteria sinensis* in New Zealand. *Plant Dis.* **2008**, *92*, 1134, doi: 10.1094/PDIS-92-7-1134B.<br>Kamińska, M.; Malinowski, T.; Rudzińska-Langwald, A.; Diaz, L.C. The occurrence of wisteria vein mosaic virus in *Wisteria floribunda* DC plants in Poland. *J. Phytopathol.* **2006**, *154*, 414-417, doi: 10.1111/j.1439-0434.2006.01118.x	*Aphis craccivora, Aphis fabae*	Valouzi, H.; Hashemi, S.S.; Wylie, S.J.; Ahadiyat, A.; Golnaraghi, A. Wisteria vein mosaic virus detected for the first time in Iran from an unknown host by analysis of aphid vectors. *Plant Pathol. J.* **2020,** *36*, 87–97, doi:10.5423/PPJ.OA.10.2019.0268

Yam mild mosaic virus	JX470965	Benin, Nigeria, Togo, China, Indonesi, Sri Lanka, Barbados, Martinique, Puerto Rico, Papua New Guinea, Fiji, Vanuatu, Solomon Islands	Plantwise KnowledgeBank on yam mild mosaic virus. Available online: https://www.plantwise.org/KnowledgeBank/datasheet/19292. (Accessed on 29 March 2020). _ Lebas, B.; Canning, E.; Kenyon, L.; Seal, S. Yam Viruses of the south pacific islands. Pest Management Dept., Univ, Greenwich, Natural Resources Institute, UK, 2002.	*Aphis craccivora*	Odu, B.O.; Hughes, J.d'A.; Shoyinka, S.A.; Dongo, L.N. Isolation, charcterisation and identification of a potyvirus from *Dioscorea alata* L. (water yam) in Nigeria. *Ann. appl. Biol.* **1999**, *134*, 65-67. doi: 10.1111/j.1744-7348.1999.tb05236.x
Yam mosaic virus	U42596	Nigeria, Benin, Ghana, Ivory Coast, Benin and Burkina Faso, Guyana, Jamaica, Barbados, Guadeloupe and Puerto Rico	Asala, S.; Alegbejo, M.D.; Olalekan, B.; Banwo, O.O; Asiedu, R.; Lava-Kumar, P. Distribution and incidence of viruses infecting yam (*Dioscorea spp.*) in Nigeria. *Global J. Biosic. Biotech.* **2012**, *1*,163-167 Brunt, A.A.; Jackson, G.V.H.; Frison, E.A.1989. FAO/IBPGR Technical guidelines for the safe movement of yam germplasm. Food and agriculture Organization of the United Nations, Rome/International Board for Plant Genetic Resources: Rome. Eni, A.O.; Hughes, J.dA.; Rey, M.E.C. Survey of the incidence and distribution of five viruses infecting yams in the major yam- producing zones in Benin. *Ann. Appl. Biol.* **2008**, *153*, 223-232, doi: 10.1111/j.1744-7348.2008.00253.x. Legg, J.P.; Gibson, R.W.; Hughes, J.d'A. Virus diseases of root crops in Africa: an overview. Proceedings of the 13th ISTRC Symposium, Rome, Italy, 2007; pp. 318 – 33. Offei, S.K. Virus and viral diseases of sub-Saharan Africa: analysis of responses to questionnaires by scientists in sub-Saharan Africa. In Proceedings of a conference organized by IITA, International Institute of Tropical Agriculture, Ibadan, Nigeria, 4-8 June 2001; pp. 128-137. Séka, K.; Etchian, A.O.; Assiri, P.K.; Toualy, M.N.Y.; Diallo, H.A.; Kouassi, N.K.; Aké, S. Yield loss caused by yam mosaic virus (YMV) and cucumber mosaic virus (CMV) on the varieties of *Dioscorea spp. Internat. J. Agron. Agri. Res*. **2014**, *5*, 64-71. Yam mosaic virus, African soil health consortium. Available online:http://africasoilhealth.cabi.org/wpcms/wp-content/uploads/2015/02/24-tubers-yam-mosaic-disease.pdf (Accessed on 29 March 2020).	*Aphis gossypii, Toxoptera citricidus, Aphis craccivora, Rhopalosiphum maidis, Aphis fabae, Myzu spersicae*	Thouvenel, J.C.; Fauquet, C. Yam mosaic, a new potyvirus infecting *Dioscorea cayenensis* in the Ivory Coast. *Ann. Appl. Biol.* **1979**, 93, 279–283. Africa soil health consortium, yam mosaic virus. Available online:http://africasoilhealth.cabi.org/wpcms/wp-content/uploads/2015/02/24-tubers-yam-mosaic-disease.pdf(Accessed on 29 March 2020).
Yambean mosaic virus	JN190431	Brazil, Peru	Medeiros, L., de Oliveira, I.A.; Pires, L.L.; Kitajime, E.W.; da Silva, G.S.; Beserra Jr, J.E.A. First report of yambean mosaic virus in Brazil. *Australas. Plant Dis. Notes* **2019**, *14*, 22, doi: 10.1007/s13314-019-0349-2. Fuentes, S.; Heider, B.; Tasso, R.C.; Romero, E.; Felde, T.Z.; Kreuze, J.F. Complete genome sequence of a potyvirus infecting yam beans (*Pachyrhizus spp.*) in Peru. *Arch Virol.* **2012**, *157*, 773–776., doi: 10.1007/s00705-011-1214-6.	Unknown	
Zantedeschia mild mosaic virus	AY626825	Taiwan, Italy, New Zealand, Australia	Huang, C.H.; Hu, W.C.; Yang, T.C.; Chang, Y.C. Zantedeschia mild mosaic virus, a new widespread virus in calla lily, detected by elisa, dot-blot hybridization and IC RT PCR. *Plant Pathol.* **2007**, *56*, 183–189, doi: 10.1111/j.1365-3059.2006.01485.x. Rizzo, D.; Panattoni, A.; Stefani, L.; Paoli, M.; Nesi, B.; Lazzereschi, S.; Vanarelli, S.; Farina, P.; Della Bartola, M.; Materazzi, A.; Luvisi, A. First report of zantedeschia mild mosaic virus on *Zantedeschia aethiopica* (L) Spreng in Italy. *J. Plant Pathol.* **2015**, *97*, 1-2, doi: 10.4454/JPP.V97I2.019. New Zealand (Unpublished, GenBank Acc. No. DQ407934).	Unknown	

			Kidanemariam, D.B.; Abraham, A.D.; Sukal, A.C.; Holton, T.A.; Dale, J.L.; James, A.P.; Harding, R.M.Complete genome sequence of a novel zantedeschia mild mosaic virus isolate: the first report from Australia and from *Alocasia sp*. *Arch Virol.* **2016**, *161*, 1079-1082, doi: 10.1007/s00705-015-2745-z.		
Zea mosaic virus	JQ692088	Iran, Israel	New Zealand (Unpublished, Genbank Acc. No. NC_018833) Seifers, D.L.; Salomon, R.; Marie-Jeanne, V.; Alliot, B.; Signoret, P.; Haber, S.; Loboda, A.; Ens, W.; She, Y.M.; Standing, K.G. Characterization of a novel potyvirus isolated from maize in Israel. *Phytopathology* **2000**, *90*, 505-513, doi: 10.1094/PHYTO.2000.90.5.505.	Unknown	
Zucchini shoestring virus	KU355553	South Africa, Zimbabwe	Ibaba, J.D.; Laing, M.D.; Gubba, A. First report of a novel potyvirus from the papaya ringspot virus cluster infecting zucchini (*Cucurbita pepo*) in KwaZulu-Natal, Republic of South Africa. *Plant Dis.* **2015**, *99*, 1289, doi: 10.1094/PDIS-02-15-0143-PDN. Karavina, C.; Ibaba, J.D.; Gubba, A. High-throughput sequencing of virus-infected *Cucurbita pepo* samples revealed the presence of zucchini shoestring virus in Zimbabwe. *BMC Res. Notes* **2020**, *13*, 53, doi: 10.1186/s13104-020-4927-3.	Unknown	
Zucchini tigre mosaic virus	KC345607	Guadeloupe, Martinique, Costa Rica, Venezuela, China, USA, France	Romay, G.; Lecoq, H.; Desbiez, C. Cucurbit crops and their viral diseases in Latin america and the Caribbean Islands: A Reviw. *J. Plant Pathol.* **2014**, *96*, 227-242, doi: 10.4454/JPP.V96I2.027. Xiao, L.; Li, Y.Y.; Tan, G.L.; Lan, P.X.; Zhong, L.; Liu, Y.; Li, R.; Li, F. First Report of zucchini tigre mosaic virus infecting several cucurbit plants in China. *Plant Dis.* **2016**, *100*, 1253, doi: 10.1094/PDIS-11-15-1318-PDN. Wang, D.; Boluk, G.; Quinto, E.A.; Hamim, I.; Borth, W.B.; Melzer, M.J.; Green, J.; Suzuki, J.Y.; Wall, M.M.; Matsumoto, T.; Sun, G. F.; Hu, J. S. First report of zucchini tigre mosaic virus infecting bitter melon (*Momordica charantia*) in Hawaii. *Plant Dis.* **2019**, *103*, 1047-1047, doi: 10.1094/PDIS-08-18-1391-PDN. Romay, G.; Lecoq, H.; Desbiez, C. Zucchini tigré mosaic virus is a distinct potyvirus in the papaya ringspot virus cluster: molecular and biological insights. *Arch Virol.* **2014**, *159*, 277-289, doi: 10.1007/s00705-013-1798-0.	*Myzus persicae*	Romay, G.; Lecoq, H.; Desbiez, C. Zucchini tigré mosaic virus is a distinct potyvirus in the papaya ringspot virus cluster: molecular and biological insights. *Arch. Virol.* **2014**, *159*, 277-289, doi: 10.1007/s00705-013-1798-0.
Zucchini yellow fleck virus	DQ641510	Italy, Israel, France, Greece, Lebanon, Syria, Libya, Iran	Vovlas, C.; Hiebert, E.; Russo, M. Zucchini yellow fleck virus, a new potyvirus of zucchini squash. *Phytopath. Mediterr.* **1981**, *20*, 123–128. Antignus, Y.; Levy, D.; Cohen, S. Characterisation of a variant of zucchini yellow fleck virus (ZYFV), a potyvirus causing a wilt disease of melons in Israel. *Ann. Appl. Biol.* **1995**, *126*, 111-120, doi: 10.1111/j.1744-7348.1995.tb05007.x. Gilbert-Albertini, F.; Lecoq, H. The characterization of a strain of zucchini yellow fleck virus found in southeastern France. *J. Phytopathol.* **1994,** *140*, 375-384, doi: 10.1111/j.1439-0434.1994.tb00621.x. Avgelis, A.D. Epidemiological studies of zucchini yellow fleck virus in Crete. *Phytopath. Mediterr.* **1985**, *24*, 208-210. Katul, L.; Makkouk, K.M. Occurrence and serological relatedness of five cucurbit potyviruses in Lebanon and Syria. *Phytopath. Mediterr.* **1987**, *26*, 36-42.	*Myzus persicae*	Vovlas, C.; Hiebert, E.; Russo, M. Zucchini yellow fleck virus, a new potyvirus of zucchini squash. *Phytopath. medit.* **1981**, *20*, 123-128.

			Svoboda, J.; Grimova, L.; Zouhar, M.; Rysanek, P.; Homa, I.H. First report of cucurbit aphid-borne yellows virus, watermelon mosaic virus, and zucchini yellow mosaic virus infecting zucchini plants in Libya. *Plant Dis.* **2015**, *99*, 558-558, doi: 10.1094/PDIS-10-14-1063-PDN. Rastgou M., Koohi-Habibi, M. Frequency of cucurbit viruses around urmia and first report of zucchini yellow fleck virus. In Proceedings of Iranian Plant Protection Congress, Tehran, Iran **2010;** pp.19.		
Zucchini yellow mosaic virus	AF127929	Algeria,Côte d'Ivoire, Egypt, Eswatini, Libya, Madagasca, Mali, Mauritius, Mayotte, Morocco, Nigeria, Réunion, South Africa, Sudan, Tunisia, Zimbabwe, China, India, Indonesia, Iran, Iraq, Israel, Japan, Jordan, Lebanon, Malaysia, Nepal, Oman, Pakistan, Saudi Arabia, Singapore, South Korea, Syria, Taiwan, Turkey, United Arab Emirates, Vietnam, Yemen, Austria, Belgium, Bosnia and Herzegovina, Bulgaria, Cyprus, Czechia, Czechoslovakia, Finland, France, German, Greece, Hungary, Italy, Netherlands, Norway, Poland, Portugal, Serbia, Slovakia, Slovenia, Spain, United Kingdom, Canada, Costa Rica, Dominican Republic, Guadeloupe, Honduras, Martinique, Mexico, Panama, Puerto Rico, United	Cabi datasheet on Zucchini yellow mosaic virus. Available online:https://www.cabi.org/isc/datasheet/57657 (Accessed on 29 March 2020).	*Acyrthosiphon kondoi, Acyrthosiphon pisum, Aphis craccivora, Aphis gossypii, Aphis middletonii, Aphis spiraecola, Aulacorthum solani, Hysteroneura setariae, Lipaphis erysimi, Macrosiphum euphorbiae, Metopolophium dirhodum, Myzus persicae, Sipha maydis, Toxoptera aurantia, Uroleucon ambrosiae*	Haque, M.N.; Kundu, A.K.; Akanda, A.M.; Bhuiyan, M.K.A. Comparative biological and serological properties of watermelon mosaic virus 2 and zucchini yellow mosaic virus. *Bangladesh J. Plant Pathol*. **2004**, *20*, 71-76, Lisa, V.; Lecoq, H. *Zucchini yellow mosaic virus. CMI/AAB Descriptions of Plant Viruses, No. 282*, Association of Applied Biologists: Wellesbourne, UK, 1984. Katis, N.I.; Tsitsipis, J.A.; Lykouressis, D.P.; Papapanayotou, A.; Margaritopoulos, J.T.; Kokinis, G.M.; Perdikis, D.C.; Manoussopoulos, I.N. Transmission of Zucchini yellow mosaic virus by colonizing and non-colonizing aphids in Greece and new aphid species vectors of the virus. *J. Phytopathol.* **2006**, *154*, 293-302, doi: 10.1111/j.1439-0434.2006.01096.x. Katis, N.I.; Tsitsipis, J.A.; Lykouressis, D.P.; Papapanayotou, A.; Margaritopoulos, J.T.; Kokinis, G.M.; Perdikis, D.C.; Manoussopoulos., I.N. Transmission of zucchini yellow mosaic virus by colonizing and non-colonizing aphids in Greece and new aphid species vectors of the virus. *J. Phytopath.* **2006,** *154*, 293–302, doi: 10.1111/j.1439-0434.2006.01096.x.

	States, Australia, Guam, New Caledonia, New Zealand, Papua New Guinea, Samoa, Solomon Islands, Tonga, Argentina, Brazil, Chile, Venezuela						
